# Metal-Dependent Photodissociation of Hydrazone Photoswitches from Rare-Earth Complexes

**DOI:** 10.1021/jacs.6c03443

**Published:** 2026-04-09

**Authors:** Matteo Melegari, Randall K. Wilharm, Rodolpho Nesta Silva, Flavia Artizzu, Angela Serpe, Dirk H. Trauner, Matteo Tegoni, Eric J. Schelter, Luciano Marchiò

**Affiliations:** † Department of Chemistry, Life Sciences and Environmental Sustainability, University of Parma, Parco Area delle Scienze 17/A, 43124 Parma, Italy; ‡ Vagelos Laboratory for Energy Science and Technology, Department of Chemistry, University of Pennsylvania, Philadelphia, Pennsylvania 19104, United States; § Department of Sustainable Development and Ecological Transition, 19050 University of Eastern Piedmont “A. Avogadro”, Piazza S. Eusebio 5, 13100 Vercelli, Italy; ∥ Department of Civil and Environmental Engineering and Architecture (DICAAR), and Research Unit of INSTM, University of Cagliari, Via Marengo 2, 09123 Cagliari, Italy; ⊥ Environmental Geology and Geoengineering Institute of the National Research Council (IGAG−CNR), Piazza d’Armi, 09123 Cagliari, Italy; # P. Roy and Diana T. Vagelos Laboratories, Department of Chemistry, 6572 University of Pennsylvania, 231 South 34th Street, Philadelphia, Pennsylvania 19104, United States; ¶ Department of Earth and Environmental Science, University of Pennsylvania, Philadelphia, Pennsylvania 19104, United States; ∇ Department of Chemical and Biomolecular Engineering, University of Pennsylvania, Philadelphia, Pennsylvania 19104, United States

## Abstract

Rare-earth element separation processes often rely on a small decrease in ionic radii along the series of elements. Separation processes based on the distinct optical properties of REs remain less explored, although photochemical methods may offer a viable alternative. Accurate selection of the synthetic precursors of a photoswitchable acylhydrazonic ligand led to a system that could quantitatively isomerize (*E–*to–*Z*) upon irradiation with commercial LED lights. Coordination of the photoswitch with RE^III^ nitrates (RE = La–Lu except Pm and Y) resulted in the retention of the photoswitching properties observed in solution. The lower binding affinity of the generated *Z*–isomer with RE^III^ ions yielded the dissociation of the complexes upon irradiation (photodissociation) with the release of RE–nitrates in solution. The rate of the reaction was found to be dependent on the optical properties of the RE^III^ ions, with nonemissive complexes (no 4f excited states) dissociating faster than emissive ones (having accessible 4f excited states). A thorough solid-state characterization of the complexes was performed by using crystallographic and photochemical methods. Ultimately, the accessibility of the 4f excited states of the metals following light irradiation and excitation of the ligand led to a decrease in the rate of the reaction due to quenching of the ligand excited state. These results demonstrate that the direct modulation of the metal coordination environment, combined with the metal-dependent reaction rate, could provide a strategy for the development of RE–separation processes based on differences in their optical properties.

## Graphical abstract

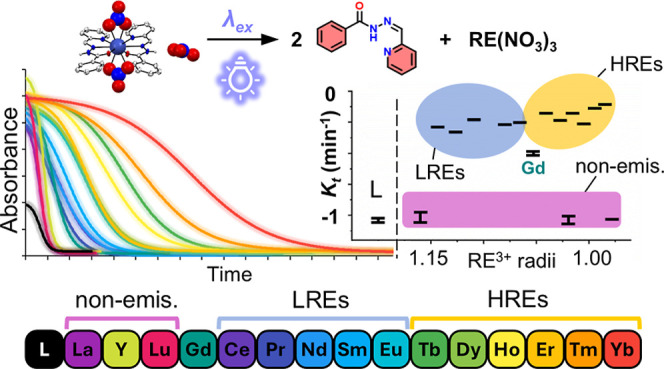

## Introduction

Rare-earth elements (REs) (La–Lu, Sc, and Y) are crucial elements for the so-called “green transition”. Their applications range from permanent magnets and battery materials, to phosphors and ceramics, which accounts for their listing as “critical materials” by both the US and the European Union.^1–3^ A variety of different methods for their separation have been proposed throughout the years. Most of these methods, however, rely on the small differences in the Lewis acidity or ionic radii of different REs, which, from La^III^ to Lu^III^, decrease by ∼15% due to the lanthanide contraction.^4^ This small change makes ionic radii/Lewis acidity-based separations challenging and most effective only for metal pairs far apart in the lanthanide series, in particular light REs (LREs) from heavy REs (HREs).^5,6^ Thus, the separation of neighboring elements often requires processes such as liquid chromatography or many cycles of liquid–liquid extraction, leading to overall non–environmentally friendly processes.^7–9^

Only in recent years has the research on RE separation based on alternative methods gained significant momentum (*e.g.,* magneto-separations).^10–17^ While photochemical strategies for lanthanide separation based on redox modulation—such as those relying on changes in the metal’s oxidation state demonstrated for europium—have been previously reported,^18,19^ to date no separation process has been developed that exploits their luminescent signatures, despite the clear potential for such properties to dictate different response behaviors.^20^ Recently, one of us reported a system that demonstrated that accessible excited 4f states in Dy^3+^ cations can influence a photochemical reaction rate as compared to an isostructural Y^III^ compound that possessed no such 4f states (Scheme 1).^21^ In this case, a decrease in the reaction rate of the Dy^III^ complex was observed compared to the isostructural Y^III^ one. This RE dependence of the reaction rate was not tied to the Lewis acidity of the metal cations as Dy^III^ and Y^III^ have nearly identical ionic radii (1.027 and 1.019 Å, respectively). As such, this system indicated that Dy/Y chemical differentiation could be achieved using excited state reactivity.

**Scheme 1:**
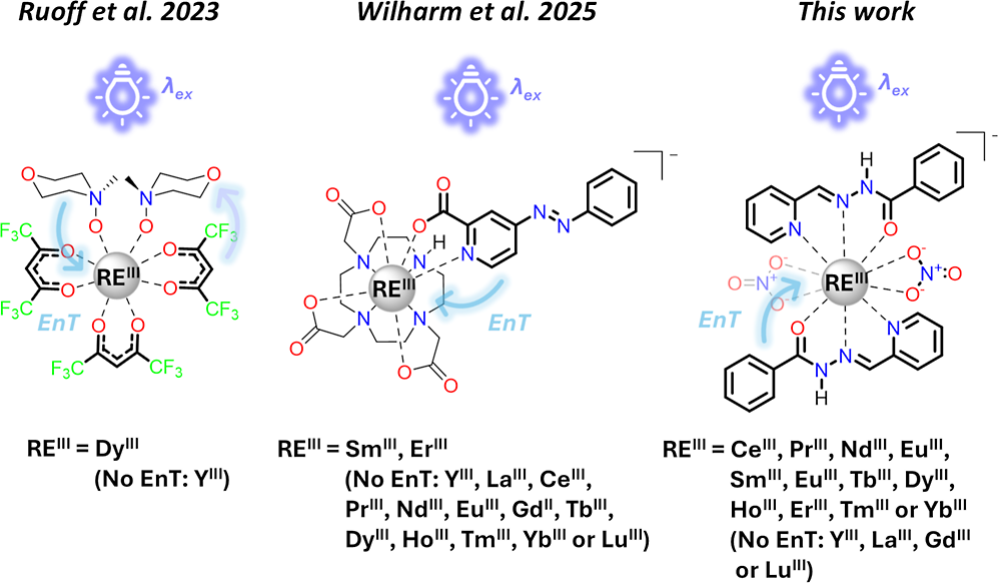
Selected Reported Photochemical Systems Wherein Specific RE^III^ Cations Influence Chemical Properties Including Reaction Rates and Photostationary States (PSS)^a^

a Light-absorbing ligand are depicted in bold.

Among photochemical reactions, isomerization reactions of photoswitches, molecules capable of changing their structure from one isomeric form to another upon light irradiation, are noteworthy.^22–32^ Coupling photoswitching ligands and metal cations has attracted attention in many fields (i.e., magnetism); in reported cases the first coordination sphere of the metal ions, and associated properties, can be tuned upon light irradiation.^33–35^ Subsequently, three of us studied RE complexes of an azobenzene-type photoswitch, Na­[RE^III^(DO3A)­(azo-pic)] (RE = Y, La, Ce, Pr, Nd, Sm, Eu, Gd, Tb, Dy, Ho, Er, Tm, Yb, or Lu) (Scheme 1).^36^ These complexes demonstrated a Lewis acidity trend in the photoisomerization of the azo-pic^–^ ligand coordinated to RE^III^DO3A, specifically the photoisomerization yield increased from La^III^ to Lu^III^, with the notable exceptions of Sm^III^ and Er^III^. Photoisomerization quenching by energy transfer (EnT) from azo-pic^–^ to Sm^III^ or Er^III^ was implicated to break in the Lewis acidity trend, and computational analysis of the excited states of [Y^III^(DO3A)­(azo-pic)]^−^ revealed good energy matching between the relevant states of the azo-pic^–^ ligand and the known 4f states of Sm^III^ or Er^III^, enabling efficient EnT. Conversely, an analogous system displayed an opposing yield profile, wherein the influence of the lanthanide contraction inverted the trend previously observed for the [RE^III^(DO3A)­(azo-pic)] complexes.^37^ These results demonstrated changes in photoswitch photostationary states based on interactions with individual RE cations, further tying photochemical properties to the prospect of single-element separations.

Hydrazones and arylhydrazones are an important class of photoswitches that have also been studied due to their interesting photoswitching properties and ease of synthesis.^38,39^ Additionally, the abundance of heteroatoms in their structures make them tunable ligands capable of binding most metal ions, from alkali metals to REs.^40–42^ Among the possible responses observed upon irradiation of hydrazone complexes are metal-dependent processes that represent two sides of the same coin: (i) complex photodissociation: where the newly formed isomer of the ligand has a lower binding affinity toward metal cations, causing its release in solution; and (ii) configurational locking: where the cations halt the isomerization of the ligand.^35,43–46^ The photoswitching properties of hydrazone complexes has been explored for alkali and transition metals; however, there are no reports on the photoisomerization behavior of RE–hydrazone photoswitch complexes.

In this work, we investigated the response of RE–acylhydrazonic complexes (RE = La–Lu, except Pm and Y) to light irradiation. The inability of the Z–isomer of the ligand to bind trivalent REs cations meant that the complexes underwent photodissociation upon irradiation. Following UV-light irradiation of the complexes, among the multiple processes possible were (1) ligand isomerization; (2) REs luminescence; and (3) nonradiative relaxation. The rate of the isomerization was dependent on these processes, with open-shell RE-complexes photodissociating at slower rates than closed-shell ones due to the absence of 4f excited states that quench the isomerization of the ligand in the latter. Ultimately, a more than 9–fold decrease in the isomerization rate was observed for the Yb^III^ complex compared to the nonemissive RE-complexes. These findings mark a significant advancement in designing RE-selective photoseparation systems, wherein the selectivity-determining step is directly tied to both the reorganization of the first coordination sphere and the contrasting close-shell/open-shell character of the metal ions.

## Results and Discussion

### Synthesis of RE–Acylhydrazone Complexes

The acylhydrazone ligand **(** *E* **)–L** ^**py**^ (Scheme 1) was synthesized following a modified literature procedure, with the pure *E*–isomer being obtained from the synthesis (see the Supporting Information for details) matching literature reports.^47^ The product was confirmed by single-crystal X-ray diffraction (SC–XRD) analysis, which matched the previously reported structure of the *E–*isomer.^48^ **RE–** *E* complexes (RE = La–Lu except Pm, and Y) were obtained according to literature procedures.^48^ The complexes precipitated from the reaction mixture after a few hours as crystalline materials by reacting **(** *E* **)–L** ^**py**^ with RE­(NO_3_)_3_ at room temperature in MeOH. Elemental analysis confirmed a ratio of 1:2:3 of RE^III^ to **(** *E* **)–L** ^**py**^ to NO_3_^–^, with neutrally charged **(** *E* **)–L** ^**py**^ ligands with variable amounts of cocrystallized solvent molecules (MeOH or H_2_O). The identity of the neutral ligand across the complexes was further supported by the observation of the N–H stretch in the associated FT–IR spectra.

The series of **RE–** *E* complexes was characterized by SC–XRD (Figure 1 and Figures S5–S23, Tables S1–19). Three types of molecular structures were observed: (1) [RE^III^((*E*)–L^py^)_2_(NO_3_)_3_]·2­(MeOH) (with RE = La, **La–** *E*); (2) [RE^III^((*E*)–L^py^)_2_(NO_3_)_3_]­[RE^III^((*E*)–L^py^)_2_(NO_3_)_2_]­(NO_3_)·(MeOH) (with RE = Ce, **Ce–** *E*); and (3) [RE^III^((*E*)–L^py^)_2_(NO_3_)_2_]­(NO_3_) (with RE = Pr–Lu, **Pr–** *E*–**Lu–** *E*, including Y and except for Pm). In the structure of **La–** *E*, the La^III^ cation adopted a 12–coordinate icosahedral geometry, coordinated by two neutral tridentate **(** *E* **)–L** ^**py**^ ligands and three bidentate nitrate anions, resulting in a neutral complex. The **RE–** *E* (RE = Pr–Lu) complexes exhibited an overall similar molecular structure across the series, although they crystallized in the triclinic *P*1̅ or monoclinic *C*2/*c* space groups. In the structures of [Pr^III^((*E*)–L^py^)_2_(NO_3_)_2_]­(NO_3_)·0.25­(H_2_O), [Nd^III^((*E*)–L^py^)_2_(NO_3_)_2_]­(NO_3_), [Sm^III^((*E*)–L^py^)_2_(NO_3_)_2_]­(NO_3_), [Eu^III^((*E*)–L^py^)_2_(NO_3_)_2_]­(NO_3_), [Gd^III^((*E*)–L^py^)_2_(NO_3_)_2_]­(NO_3_), [Tb^III^((*E*)–L^py^)_2_(NO_3_)_2_]­(NO_3_), [Dy^III^((*E*)–L^py^)_2_(NO_3_)_2_]­(NO_3_), [Y^III^((*E*)–L^py^)_2_(NO_3_)_2_]­(NO_3_), [Ho^III^((*E*)–L^py^)_2_(NO_3_)_2_]­(NO_3_), [Tm^III^((*E*)–L^py^)_2_(NO_3_)_2_]­(NO_3_), [Yb^III^((*E*)–L^py^)_2_(NO_3_)_2_]­(NO_3_)·1.5­(H_2_O), and [Lu^III^((*E*)–L^py^)_2_(NO_3_)_2_]­(NO_3_), the RE^III^ ions adopted a 10–coordinate bicapped square antiprismatic geometry, coordinated by two neutral tridentate **(** *E* **)–L** ^**py**^ ligands and two bidentate nitrate anions, resulting in cationic complexes. Charge balance was provided by one outer sphere nitrate anion in each case, which engaged in hydrogen bond interactions with the NH groups of the ligands. The structure of [Er^III^((*E*)–L^py^)_2_(NO_3_)_2_]­(NO_3_)·1.5­(H_2_O) was previously reported in the literature and matched the structure reported in this work for **Yb–** *E*.^48^ Notably, the structure of **Ce–** *E* revealed two different complex entities in the asymmetric unit. One complex entity exhibited a “lanthanum–like” coordination with a 12-coordinate icosahedral geometry, coordinated by two tridentate **(** *E* **)–L** ^**py**^ ligands and three bidentate nitrate ions. In the second complex entity, the Ce^III^ ion adopted a 10-coordinate bicapped square antiprism, coordinated by only two bidentate nitrate ions and two tridentate ligands, exhibiting a “praseodymium–like” coordination. As in the case of the rest of the **RE–** *E* series, the charge balance was provided by one outer sphere nitrate anion.

**Figure 1:**
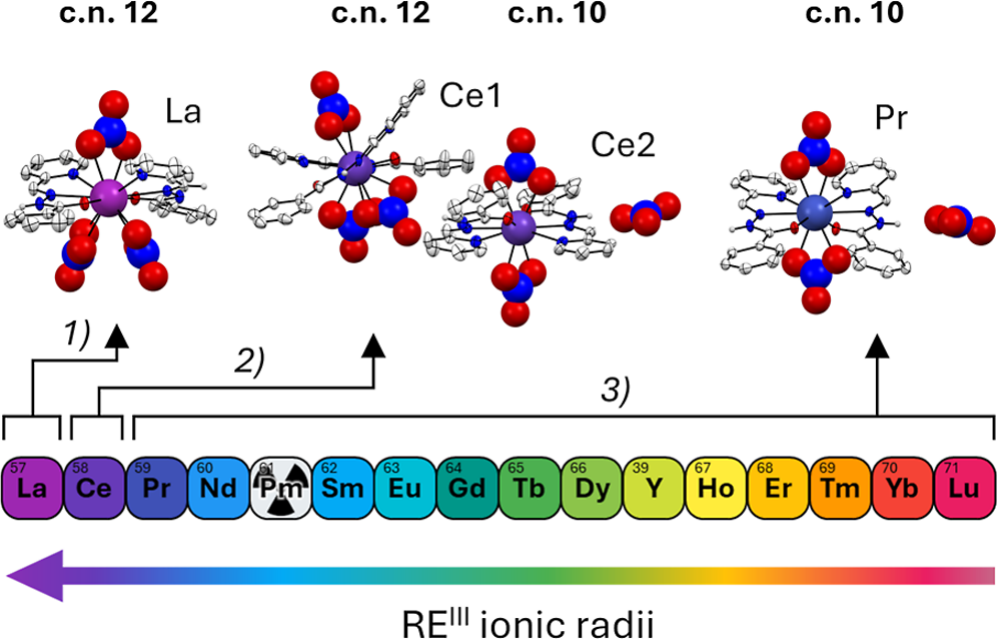
Structural difference in the complex units of **RE–** *E* structures highlighting the three main groups: (1) **La–** *E*: coordination number 12, neutral complex; (2) **Ce–** *E*: coordination number 12, neutral complex and coordination number 10, cationic complex plus nitrate; (3) **Pr–** *E* to **Lu–** *E*: coordination number 10, cationic complex plus nitrate.

The ability of the metal ions to adopt higher coordination numbers, at least in the solid state, was evidently only possible for the larger cations La^III^ and Ce^III^, as commonly reported for rare-earth complexes.^49^ It is worth noting that the structure of the solvate **[**Ce^III^((*E*)–L^py^)_2_(NO_3_)_3_]·2­(acetone)·2­(H_2_O) was previously described, in which only the “lanthanum–like” (coordination number of 12) geometry of the metal ion was reported.^50^ This variable coordination number behavior of Ce^III^ may be explained by the use of different crystallization solvents as we used MeOH whereas the reported structure was crystallized from acetone. Having established the solid state properties of complexes across the RE series, we next turned to their photophysical properties.

### Ligand Photoswitching Properties and Metal Complexation

For a photoswitch to be considered ideal, the two isomeric forms should have significantly different absorption spectra (both in terms of wavelength of the absorption bands and molar extinction coefficients).^32^ According to the first principle of photochemistry, which can be summarized as “if it does not absorb, it does not react”, the highest conversion yield upon irradiation of the *E*–isomer to the *Z*–isomer would be observed using an irradiation wavelength capable of strongly interacting (being absorbed) by the first form but not by the other.^51^ This is possible only if the maximum absorption of the two isomers is well separated and the former exhibits absorption bands where the other form does not absorb, commonly observed for hydrazones and acylhydrazones.^47^ Since the photoisomerization efficiency is highly dependent on the irradiation wavelength, wavelength scans were performed on the ligand to identify the light source that provides the highest isomerization yield (Figure S24).

A 40 μM methanolic solution of **(** *E* **)–L** ^**py**^ was illuminated at room temperature with different monochromatic wavelengths (from 440 to 300 nm with steps of 20 nm, illuminating for 10 min at each wavelength), recording the absorption spectra (in the 250–450 nm range) after each irradiation. The biggest difference in the absorption spectra of the ligand before and after irradiation was observed using a wavelength of 380–400 nm as the absorbance of the band at 365 nm after irradiation with these wavelengths dropped to almost zero. This range is exactly in the region where the emission of common “black light” LEDs occurs (maximum emission at 395 nm, Figure S25). Therefore, the absorption spectrum of the ligand (30 μM in MeOH at room temperature) was recorded before and after irradiation using the 395 nm LED strip in a homemade photoreactor (Figure 2), ensuring the reaching of the PSS (Figures S26–S27). The PSS composition was determined through ^1^H NMR spectroscopy, and complete conversion of **(** *E* **)–L** ^**py**^ to **(** *Z* **)–L** ^**py**^ (*E*:*Z* ratio 0:100 after irradiation) was confirmed by the absence of the ^1^H NMR resonances of the starting isomer after 50 min of irradiation of a 3.5 mM **(** *E* **)–L** ^**py**^ in MeOH–*d*_*4*_ inside a borosilicate NMR tube (Figure S28–S29, Table S20). The ligand showed high bistability with minimal *Z*– to the *E–*conversion at room temperature, congruent with previous reports (see the Supporting Information).^43^

**Figure 2:**
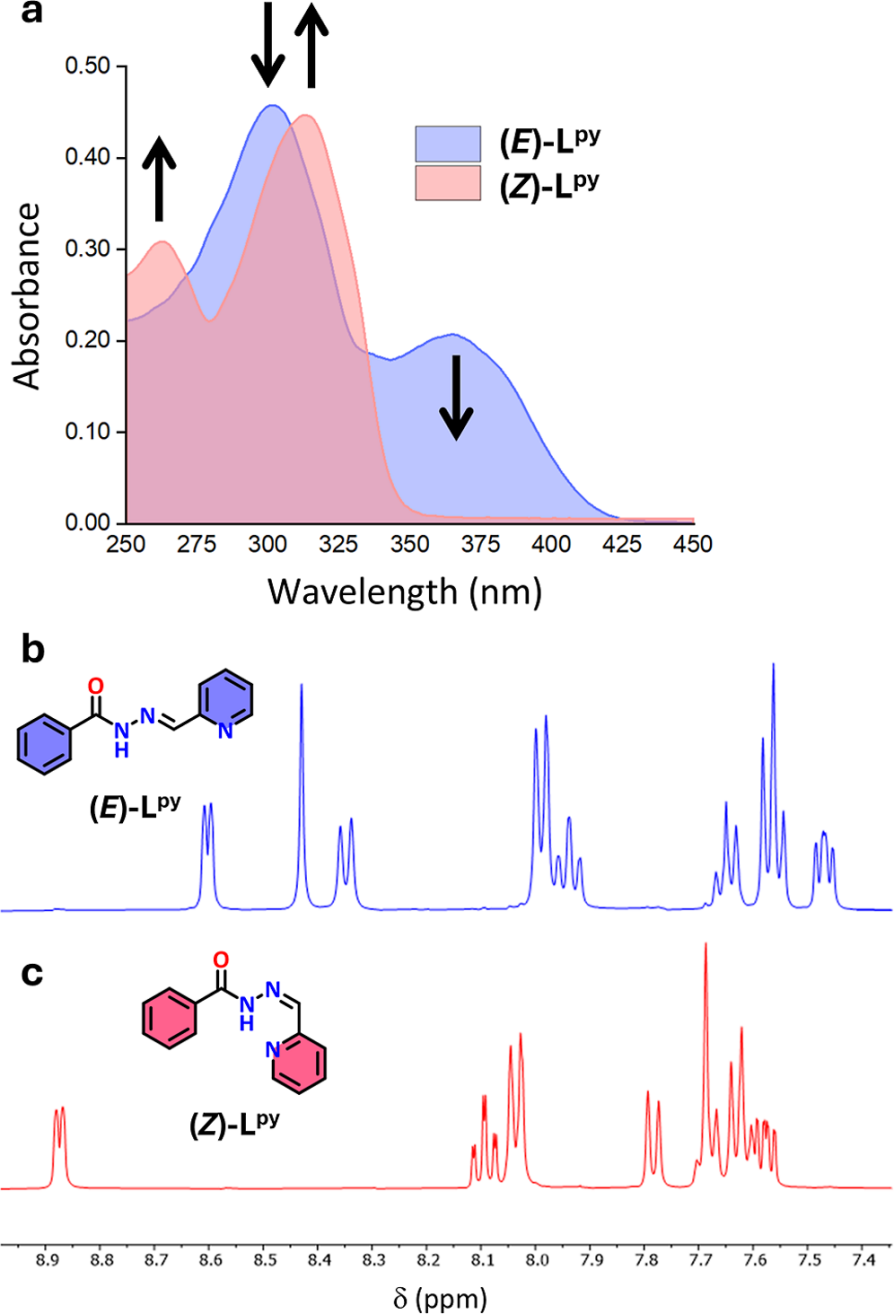
(a) UV–vis spectra of a 30 μM solution of **(** *E* **)–L** ^**py**^ in MeOH before (light blue line) and after (pink line) irradiation inside a 395 nm LED photoreactor for 10 min at room temperature. The UV–vis spectra correspond to the pure **(** *E* **)–L** ^**py**^ and **(** *Z* **)–L** ^**py**^ ligand, respectively. ^1^H NMR spectra (400 MHz, MeOH–*d*_*4*_) of a 3.5 mM solution of **(** *E* **)–L** ^**py**^ (b) before irradiation and (c) after irradiation using a 395 nm photoreactor for 60 min, corresponding to the pure **(** *Z* **)–L** ^**py**^ ligand.

The formation constants for the entire **RE–** *E* series of complexes were determined in MeOH through UV–vis titrations at room temperature. Upon addition of RE–nitrates to 15 μM solutions of **(** *E* **)–L** ^**py**^ in MeOH at room temperature, the absorption band related to the ligand (centered at 303 nm) decreased in absorbance concomitantly to an increase in absorbance of the band at 365 nm (Figure 3). In agreement with the isolated compounds, the model that best fit the data was represented by the formation of just one complex species, with a metal-to-ligand ratio (M:L) of 1:2 (Figures S31–S32). The complexes exhibit similar logarithmic formation constants (10.9–12.4). Notably, lanthanides at the end (Tm–Lu) and especially at the beginning (La–Pr) of the series form slightly less stable complexes than those in the middle (Table S21).^52^

**Figure 3:**
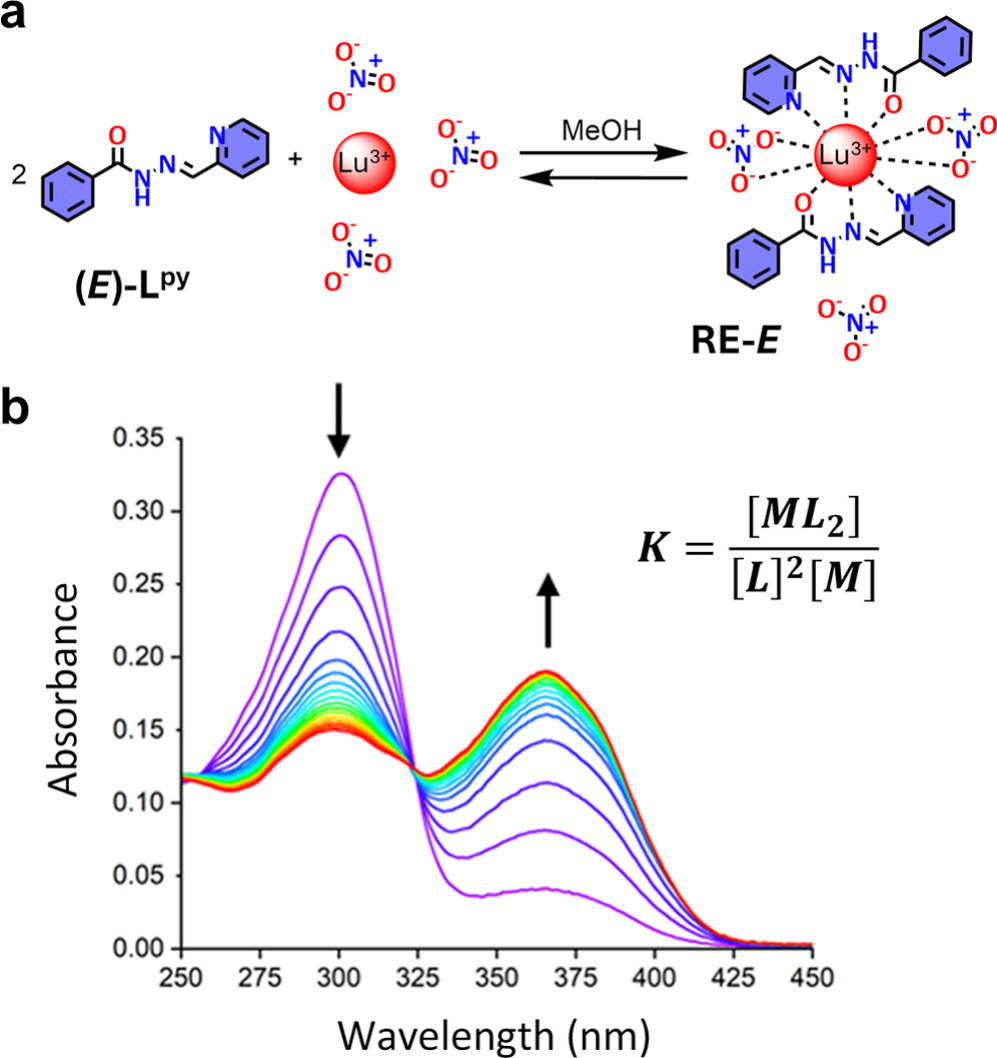
(a) Equilibrium reaction for the complexation of Lu^III^ with **(** *E* **)–L** ^**py**^. (b) UV–Vis spectra depicting the titration of 15 μM **(** *E* **)–L** ^**py**^ with Lu­(NO_3_)_3_ in MeOH at room temperature. Spectra are color-coded based on the addition of the titrant, ranging from 0 (**(** *E* **)–L** ^**py**^, purple line) to 3.44 M:L ratio (red line).

The weak binding affinity of **(** *Z* **)–L** ^**py**^ toward RE^III^ cations was confirmed by UV–Vis titration of a solution containing the starting **(** *E* **)–L** ^**py**^ ligand (15 μM in MeOH at room temperature) irradiated inside the 395 nm LED photoreactor, at room temperature and for 3 min, to obtain complete conversion to **(** *Z* **)–L** ^**py**^. Following irradiation, the titration was performed by addition of the RE–nitrate salts (La^III^, Lu^III^, and Y^III^). No binding was observed for the three metals with **(** *Z* **)–L** ^**py**^ in the experimental conditions as the changes in the absorbance throughout the titration could be ascribed only to dilution effects and not to metal complex formation (Figures S32–S33). Indeed, this behavior was described previously as **(** *Z* **)–L** ^**py**^ can act only as a bidentate ligand with a relatively low binding affinity, unlike **(** *E* **)–L** ^**py**^ which can act as a tridentate chelator.^43,53^ The relatively strong binding affinity of **(** *E* **)–L** ^**py**^ toward RE^III^ cations compared to the weak affinity of **(** *Z* **)–L** ^**py**^ indicated the possibility of this system to undergo ligand photodissociation. If the strength of this effect varies across different RE ions, it may be possible to achieve selective metal release, opening the door to photoinduced separation. As such, we next considered the photoswitching properties of the complexes.

### Photoswitching Properties of RE Complexes

The photoswitching behavior of complexes **RE–** *E* was evaluated. Wavelength scans were performed on the **Nd–** *E* complex (10 μM in MeOH at room temperature). Similar to the free ligand, the wavelength that produced the largest difference in the absorption spectra was 380–400 nm, suggesting that RE coordination does not significantly alter the wavelength that induces the most efficient isomerization (Figure S25). The determination of PSS compositions was evaluated by irradiation of borosilicate NMR tubes containing 5.0 mM **La–** *E* and **Y–** *E* in MeOH–*d*_*4*_ for 10 min using the 395 nm LED photoreactor, and the ^1^H NMR spectra were recorded before and after irradiation (Figure S35). Due to the diamagnetism of the **La–** *E* and **Y–** *E* complexes, the peaks in the ^1^H NMR spectra were well resolved and could be assigned to the protons of the *E*–isomer bound to the metals. Notably, only one set of resonances formed upon irradiation, and they were essentially identical with that of **(** *Z* **)–L** ^**py**^ obtained from the irradiation of the pure **(** *E* **)–L** ^**py**^ ligand (Figure 4). The absence of the **La–** *E* and **Y–** *E* metal complex in the ^1^H NMR resonances after irradiation suggested the quantitative conversion from the *E*– to the *Z–*isomer of the ligand even when involved in metal complexation (Figures S37–S38). Additionally, due to the low binding capabilities of **(** *Z* **)–L** ^**py**^ toward RE^III^ ions, we suggest the release of RE­(NO_3_)_3_ in solution together with the *Z–*ligand, implying that the complexes undergo photodissociation. No isomerization was observed by irradiation of solid samples of the **Y–** *E*, **Lu–** *E*, and **(** *E* **)–L** ^**py**^ in the 395 nm LED photoreactor by subsequent dissolution and ^1^H NMR spectroscopy analysis of the samples after irradiation (Figure S39). This behavior is typical of arylhydrazones, whose isomerization usually involves rotation around the C=N double bond or via the S_1_ excited-state population—a process that is less favorable when the molecules are tightly packed in the crystalline state.^54–61^

**Figure 4:**
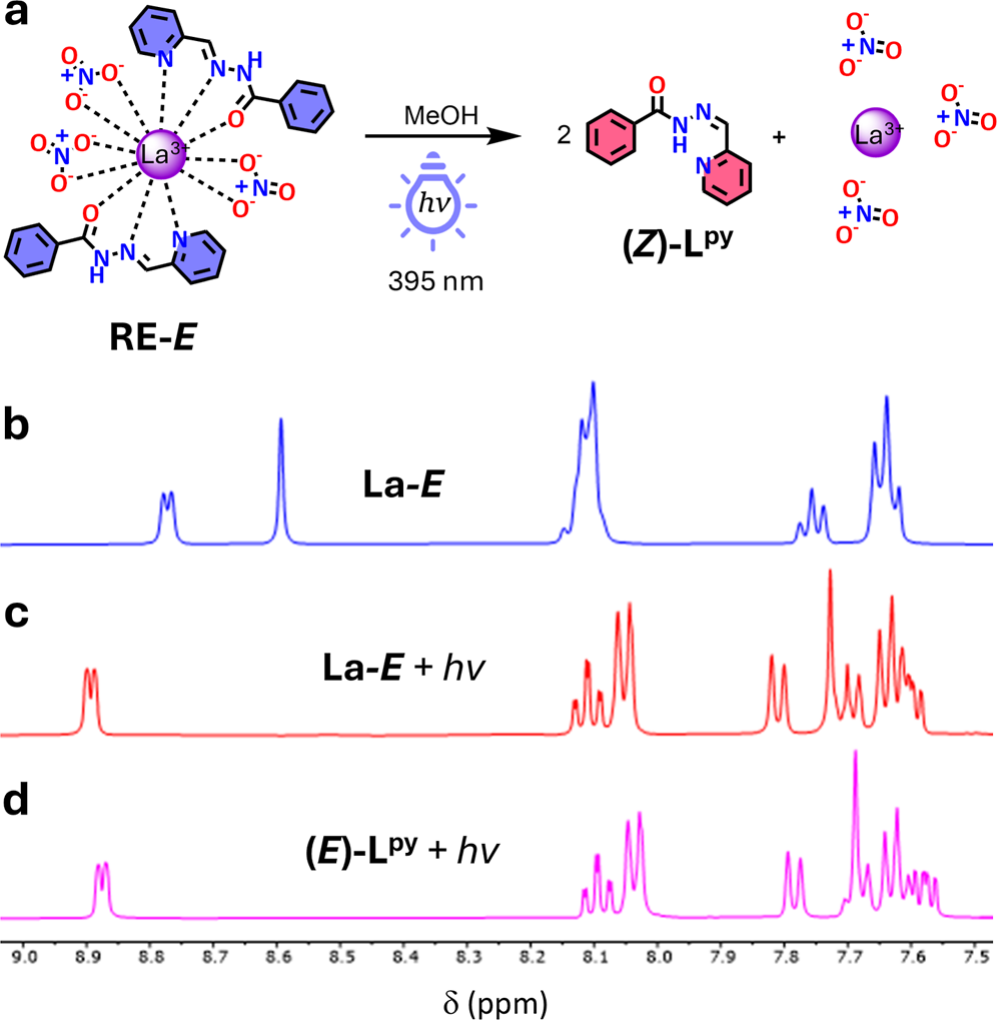
(a) Proposed reaction of **La–** *E* complexes upon irradiation. ^1^H NMR spectrum (400 MHz, MeOH–*d*_*4*_) of a 5.0 mM solution of **La–** *E* (b) before and (c) after irradiation with the 395 nm LED photoreactor for 60 min. (d) ^1^H NMR spectrum of a 10 mM solution of **(** *E* **)–L** ^**py**^ after irradiation with the 395 nm LED photoreactor for 60 min showing 100% conversion to **(** *Z* **)–L** ^**py**^.

Further confirmation of the hypothesis of photodissociation came from the irradiation of the **Nd–** *E* and **Dy–** *E* complexes (5.0 mM in MeOH–*d*_*4*_ at room temperature) and ^1^H NMR analyses. The starting ^1^H NMR spectra did not reveal any resonances in the −20 to +30 ppm region due to the paramagnetism of the two RE^III^ ions. Upon irradiation, however, a new set of resonances was clearly visible in the two samples, that again corresponded to the unbound *Z*–isomer, that was especially visible in the case of **Nd–** *E* (Figure S36). We reasoned that the ability to discern the resonances of **(** *Z* **)–L** ^**py**^ in the presence of Nd^III^ and Dy^III^ ions was an additional indication of the photodissociation of the complexes due to the weak interaction of the Z–isomer with the metal cations.

Photokinetic experiments were recorded for the free ligand and for the entire **RE–** *E* series of complexes (RE = La–Lu, except Pm and Y; 15 μM in MeOH at room temperature). The UV–Vis spectra (in the range 250–500 nm) were recorded every 6 s under continuous irradiation with a 390 nm monochromatic wavelength until the change in the absorbance at 365 nm (band corresponding to the complex) plateaued. The experiments were carried out in triplicate, and the average absorbances were plotted for each wavelength (Figures S42–S57 and Figure S60). The absorbance profiles at 365 nm did not result in a linear decrease of the absorbance and were fitted with the Boltzmann Sigmoidal Equation (Figure S58 and Table S24), and the results are reported in Figure 5. Since the **(Z)–L** ^**py**^ generated during the photodissociation reaction has a weak absorbance at 365 nm, the traces monotonically decrease to an almost null value of absorbance, suggesting the photodissociation was quantitative for the entire series of **RE–** *E* complexes on variable time scales. Additionally, the nonlinear decrease in absorbance was ascribed to the presence of concomitant equilibrium reactions: (1) formation of the complexes **RE–** *E*; (2) photodissociation of the **RE–** *E* complexes; and (3) free ligand isomerization (Figure S61). According to the calculated log­(K) values obtained for the entire series (range of log­(K) values: 10.9–12.5), the percentage of **(** *E* **)–L** ^**py**^ free ligand before the isomerization is in the 7–22% range, which can interact with the metal released after the photodissociation thus replenishing the concentration of the **RE–** *E* complexes in the initial stages. These conclusions were further supported by **Lu–** *E* photodissociation experiments performed in excess of Lu^III^. These conditions promote a reduced concentration of the free ligand. As a consequence, the absorbance decrease of the ligand as a function of the illumination time follows a more traditional zero-order reaction kinetics without a sigmoidal profile (Figure S62). The free ligand **(** *E* **)–L** ^**py**^ showed a rapid decrease in the absorbance that stabilized to a value close to zero after 7 min of irradiation. A similar behavior, in terms of kinetics, was observed for the nonemissive **La–** *E*, **Lu–** *E*, and **Y–** *E*, suggesting that these metal ions did not alter significantly the kinetic profile for the isomerization of the hydrazonic ligand and remarkably, slower isomerizations were observed for open-shell **RE–** *E* complexes. Moreover, the similar behavior of **La–** *E* and **Lu–** *E* confirmed that the ionic radius or Lewis acidity of the cation did not significantly impact the kinetic response of the complexes. These results also confirmed that the differences in the metal coordination observed in the solid state for **La–** *E* did not produce a difference in the photoisomerization reaction in solution compared to the other nonemissive **RE–** *E* complexes. This result is different from those reported by us recently for the Na­[RE^III^(DO3A)­(azo-pic)] system comprising the azobenzene-containing azo-pic^–^ ligand. In that case, a pronounced Lewis acid effect was observed on the measured PSS across the series, probably related to the higher electrostatic attraction produced by the negative charge of the ligand, compared to the neutrally charged acylhydrazone reported in this work. On the other hand, the effect of 4f excited states quenching was observed only in the case of Sm^III^ and Er ^III^ for azobenzenes, whereas in this work, we observed the absence of this effect only in **RE–** *E* complexes of non-emissive rare earths (La^III^, Y^III^, and Lu^III^).

**Figure 5:**
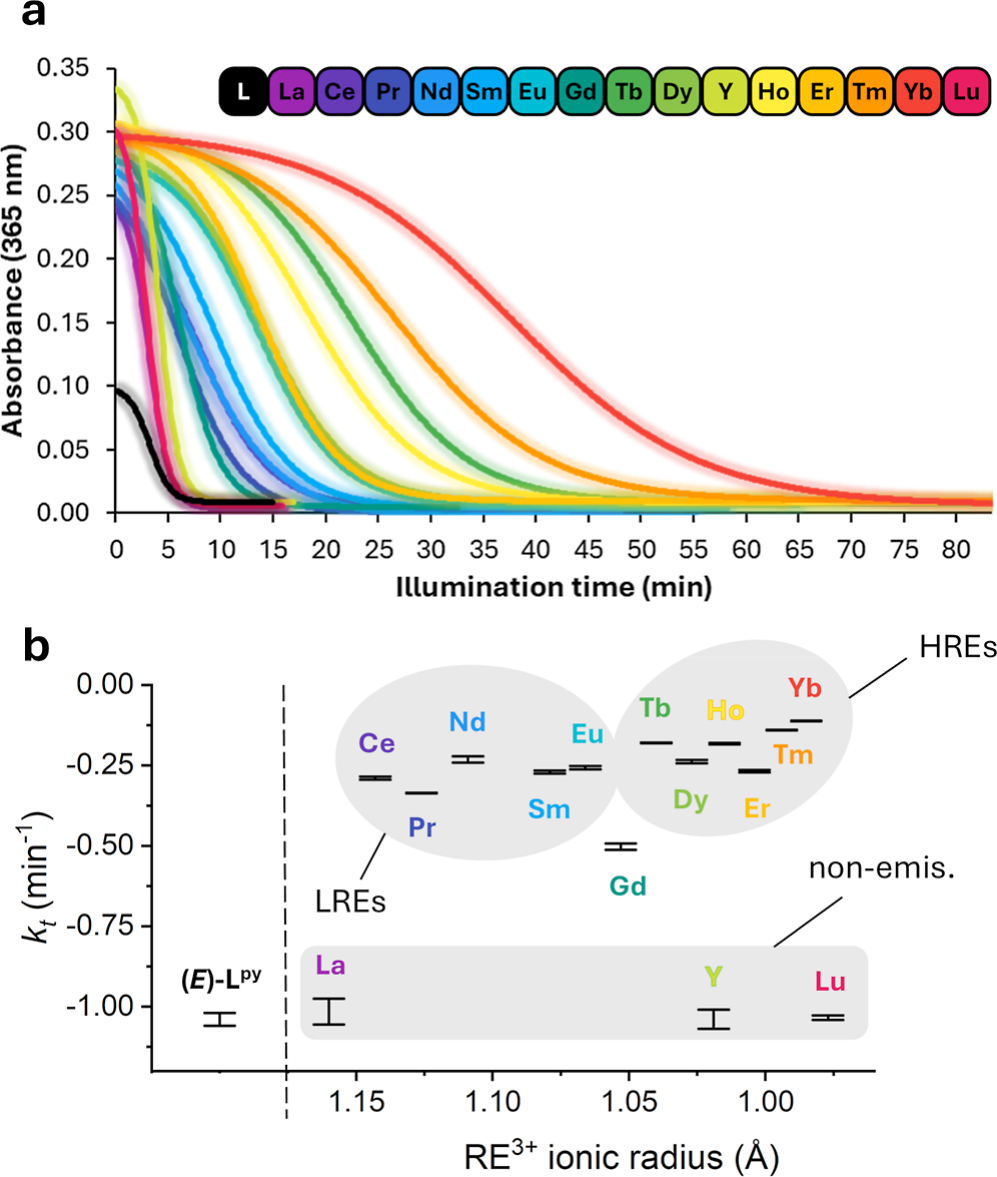
(a) Boltzmann sigmoidal fitting of the photokinetic experiments of **RE–** *E* complexes (15 μM) and **(** *E* **)–L** ^**py**^ (30 μM) in MeOH at room temperature, showing the trace of the absorbances at 365 nm while using a 390 nm monochromatic wavelength for irradiation. (b) Photodissociation parameters obtained by the Boltzmann sigmoidal equation fitting of the photokinetic experiments for **RE–** *E* complexes versus RE^III^ ionic radius. Regions of the nonemissive REs, emissive LREs, and emissive HREs were arbitrarily indicated in gray.

The measured photodissociation parameter (*k*_*t*_) for this system, corresponding to the slope of the associated sigmoidal curves, was about −1.03 min^–1^ for the ligand and nonemissive **RE–** *E* complexes, with half–lives (*t*_1/2_, time taken for the absorbance to become half the starting value) in the 2.9–4.0 min range, Table 1. Conversely, when **RE–** *E* complexes with accessible 4f excited states were irradiated, a decrease in absorbance related to the isomerization of the ligand occurred at a slower rate. The behavior of gadolinium was exactly in between the emissive and nonemissive REs as for **Gd–** *E*, the isomerization rate was half that of the nonemissive compounds (*k*_*t*_ = −0.50(1) min^–1^), presumably associated with increased ISC or with the relatively high energy 4f state associated with the Gd^III^ cation. The isomerization of LREs complexes with partially filled 4f shells exhibited rates similar to each other, with half**–**lives ranging from 6.0 to 13.4 min and *k*_*t*_ values in the range of −0.336(8) to −0.23(1) min^–1^. Interestingly, the measured photodissociation of HREs complexes with partially filled 4f shells was slower with *t*_1/2_ values of 13.7–37.5 min and *k*_*t*_ of −0.111(1) to −0.268(4) min^–1^. Complex **Yb–** *E* exhibited the slowest isomerization, >9× slower than the isomerization of the nonemissive **RE–** *E* complexes.

**Table 1:** Fitting Parameters for the Photokinetic Experiments of (*E*)–L^py^ and RE–*E* Using a 390 nm Monochromatic Wavelength and Fitted Using Boltzmann Sigmoidal Curves^a^

compound	*t*_1/2_ (min)	*k*_*t*_ (min^–1^)
**La–** *E*	3.1(1)	–1.02(4)
**Ce–** *E*	7.7(1)	–0.289(5)
**Pr–** *E*	6.0(1)	–0.336(8)
**Nd–** *E*	6.3(3)	–0.23(1)
**Sm–** *E*	9.2(1)	–0.271(5)
**Eu–** *E*	13.4(1)	–0.257(5)
**Gd–** *E*	5.9(1)	–0.50(1)
**Tb–** *E*	22.2(1)	–0.180(1)
**Dy–** *E*	13.7(1)	–0.238(5)
**Y–** *E*	4.0(1)	–1.04(3)
**Ho–** *E*	18.0(1)	–0.182(2)
**Er–** *E*	13.7(1)	–0.268(4)
**Tm–** *E*	26.3(1)	–0.140(1)
**Yb–** *E*	37.5(1)	–0.111(1)
**Lu–** *E*	2.9(1)	–1.03(7)
**(** *E* **)–L** ^**py**^	3.2(1)	–1.04(2)

a Standard deviations are reported in parentheses and refer to the last significant digit.

### Photophysical Characterization of RE Complexes

To further elucidate the differences in behavior between the open- and closed-shell complexes observed in the photokinetic studies, photoluminescence (PL) characterization of the complexes was also performed in the solid state to avoid photodissociation. Diffuse reflectance (DR) spectra of **RE–** *E* complexes and the free ligand **(** *E* **)–L** ^**py**^ were recorded in the UV, visible, and near**-**infrared (NIR) regions (200**–**2000 nm). The absorption band of electronic transitions of the coordinated **(** *E* **)–L** ^**py**^ ligand ranged from 200 to 450 nm, while in the remaining region of the spectra, the narrow peaks related to the f–f transitions of RE^III^ ions were observed (Figure S67).

Figure 6 schematically depicts the typical photoluminescence mechanism occurring in emissive RE complexes with organic ligands acting as photosensitizers (antenna). The photocycle commonly involves low**–**lying ligand triplet states that are populated after photoexcitation and intersystem**–**crossing (ISC) from the ligand excited singlet state. EnT can subsequently occur to 4f emissive levels of RE^III^ ions, provided they lie below (∼2500**–**3000 cm^–1^) the ligand donor triplet state, giving rise to intra**–**shell, narrow**–**band RE**–**centered emission. Since La^III^, Y^III^, Gd^III^, and Lu^III^ do not possess accessible (or easily accessible) excited 4f electronic states, the emission spectra of their complexes exhibited only broadband ligand**-**centered fluorescence (Figures S68–S69) in the blue spectral range (∼350–500 nm) at room temperature. The broadband blue emission of **Ce–** *E* was attributed to the overlap of ligand fluorescence and the 5d^1^ → 4f^1^ transition of Ce^III^.^62^ The energy of the first triplet excited state of the ligand (T_1_ ∼ 20900 cm^–1^) was estimated from the emission spectrum of **Gd–** *E* (Figure S70) and agreed with the DFT**-**calculated value for the free **(** *E* **)–L** ^**py**^ ligand (∼20800 cm^–1^) (Table S30). As evidenced from the PL measurements reported in Figure 6, upon photoexcitation into the ligand absorption band at 360 nm, all the investigated **RE–** *E* complexes containing the typically emissive RE^III^ ions gave rise to narrow**–**band 4f PL in the visible (Pr^III^, Sm^III^, Eu^III^, Ho^III^) and NIR (Pr^III^, Nd^III^, Er^III^, Tm^III^, Ho^III^, Yb^III^) spectral ranges, except for **Tb–** *E* and **Dy–** *E* (Table S26) while the excitation spectra closely resembled the absorption profile of the complexes. The absence of any detectable emission in **Tb–** *E* and **Dy–** *E* is likely ascribable to the reduced energy gap (<1500 cm^–1^) of the ligand’s donor triplet state and the Tb^III^ and Dy^III^ “receiving” levels,^63^ as highlighted in the Dieke diagram in Figure 6, which prevents the efficient sensitization of the metal-centered luminescence, as it will be explained later.^64^ These results are again different from what was observed for the Na­[RE^III^(DO3A)­(azo-pic)] system, where 4f-based emission was not observed from any member of the series due to the ligands nature.^36,65^

**Figure 6:**
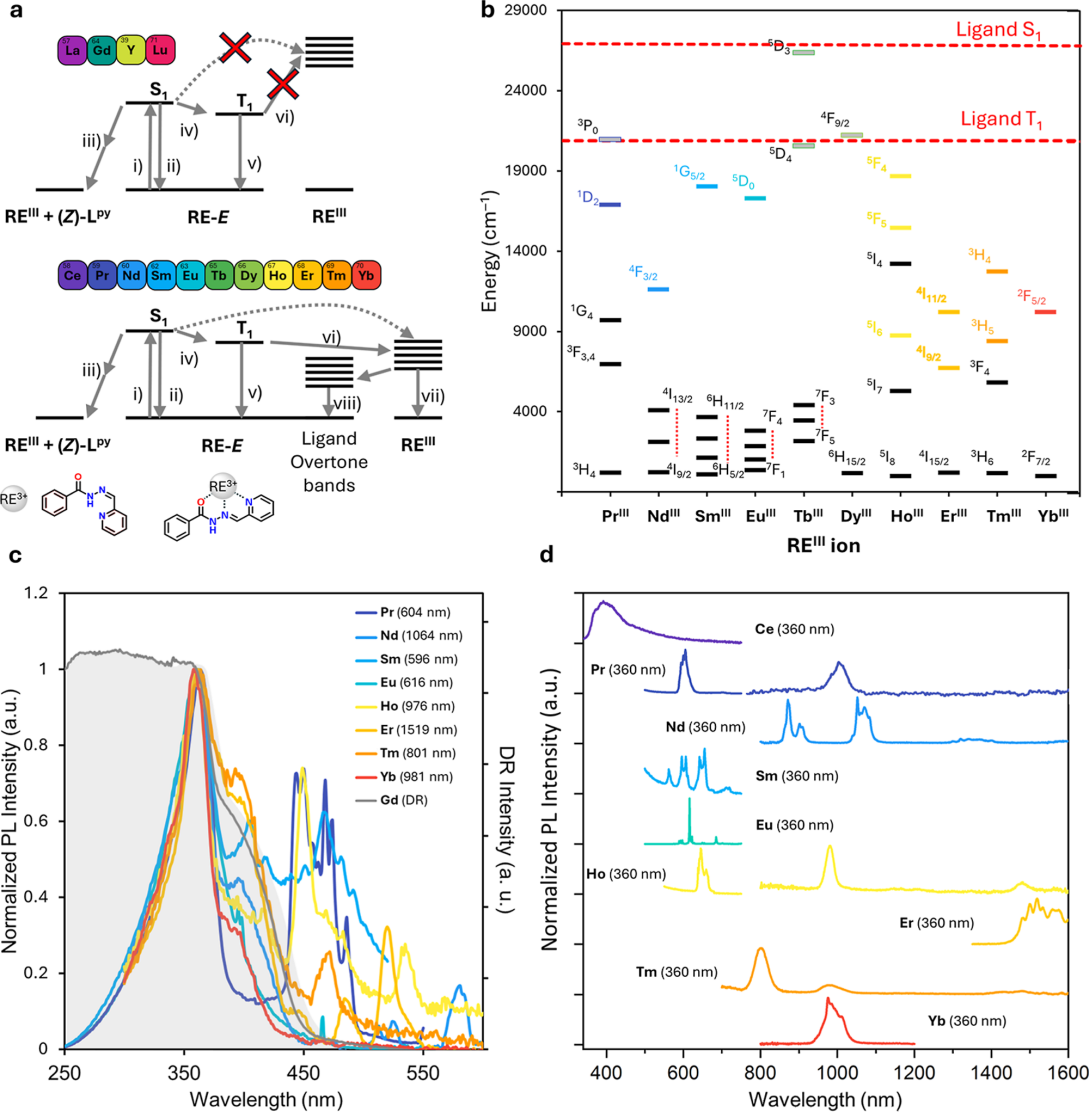
(a) Jablonski**–**type diagrams of the ligand and metal excited states for **RE–** *E* complexes and possible photocycle pathways: (i) excitation; (ii) fluorescence; (iii) isomerization; and photodissociation; (iv) ISC; (v) phosphorescence; (vi) EnT; (vii) RE–luminescence; (viii) nonradiative decay (overtone stretching bands); (b) partial Dieke diagram reporting the emissive energy ^2S+1^Γ_J_ levels of RE^III^ ions compared to the estimated ligand triplet energy level T_1_ (dashed red line). Colors evidence the emissive levels and transition ending levels are represented in black. High–lying emissive levels which do not act as energy acceptors in **RE**–*E* complexes are reported in gray. (c) PL excitation spectra of emissive RE complexes monitored at corresponding f–f transition wavelengths as indicated in the legend. The shaded gray area indicates the solid-state DR spectrum of **Gd** *–E*. Narrow bands were attributed to RE^III^ f–f transitions. (d) PL emission spectra of **RE**–*E* complexes excited in the ligand absorption band at 360 nm.

Time-resolved PL measurements were performed on **RE**–*E* complexes upon pulsed excitation at 360 or 375 nm (see the Experimental Section). Decay curves for RE-centered emission of **Pr**–*E*, **Nd–** *E*, **Sm–** *E*, **Eu–** *E*, **Ho–** *E*, **Er–** *E*, **Tm–** *E*, and **Yb–** *E* were fitted, where possible, with a monoexponential decay function (Figures S71–S72 and Table S27) indicating the existence of a single population of emitters in the crystalline materials, as expected from the crystallographic data. The relatively short decay time constants were likely a result of strong vibrational quenching effects by the superior overtones of CH, NH, and OH groups in the surroundings of the RE^III^ ion emitter, as evidenced in the UV–vis–NIR DR spectra (Figure S67 and Table S25).^66^

From the luminescence experiments, and on the same rationale reported by some of us previously, we hypothesize that the luminescent REs become excited through EnT from the first triplet excited state of **(** *E* **)–L** ^**py**^ ligand, after that, ISC and EnT to the metal cations, with the latter able to quench the excited state of the ligand through radiative (RE**–**luminescence) or nonradiative relaxation.^21^ On the other hand, no ligand**–**to**–**metal EnT can occur in the case of the nonemissive La^III^, Y^III^, Gd^III^, and Lu^III^, as previously mentioned. Therefore, photoexcitation is more likely to result in ligand isomerization, in agreement with the measured dissociation parameters.

To further investigate the ligand**–**to**–**metal EnT efficiency, we compared the emission intensity of the ligand residual fluorescence of the **RE–** *E* complexes displaying RE**–**centered emission with that of **Gd–** *E*. Gd**-**based complexes are often taken as useful reference in this regard since the spin**–**orbit coupling effect of Gd^III^ (favoring ISC) is comparable to the other luminescent REs but no triplet deactivation by the RE ion is observed. In **Pr–** *E*, **Nd–** *E*, **Sm–** *E*, **Eu–** *E*, **Tb–** *E*, **Dy–** *E*, **Ho–** *E*, **Er–** *E*, **Tm–** *E*, and **Yb–** *E*, the fluorescence intensity of the ligand dramatically drops with respect to that observed in **Gd–** *E*, and we assume that this is a reliable indirect evidence of efficient ligand**–**to**–**metal EnT (Figure S73). In fact, the depopulation of the excited triplet state of the ligand by REs feeding significantly boosts the singlet–triplet intersystem crossing (ISC) dynamics, resulting in a decreased fluorescence signal.^67^ EnT efficiencies estimated values based on steady-state data (see the Supporting Information for details) fall in the 73**–**96% range (Table S28 and Figure S74). As can be seen, the series visible emitters **Pr–** *E*, **Sm–** *E*, **Eu–** *E*, and **Ho–** *E* show the largest (>90%) EnT efficiencies, whereas NIR emitters (**Nd–** *E*, **Er–** *E*, **Tm–** *E*, and **Yb–** *E*) display lower values. This behavior can be reliably ascribed to an energy gap effect, that is, a better energy match (<∼3000 cm^–1^) between the ligand’s triplet donor level and the highest emissive levels of visible emitters with respect to NIR emitters (Figure S75).^68^ However, the observed trend presents significant deviations for the visible emitters **Tb–** *E* and **Dy–** *E* (EnT efficiencies <85%), which correlate with the absence of any sensitized emission in these compounds. Moreover, no luminescence is detected for **Dy-** *E* even in the NIR range, even though Dy^III^ possesses low-lying NIR-emissive levels that can be in principle sensitized by direct EnT from the ligand (Figure S76).^63^ Indeed, strong ligand-sensitized NIR emission is observed in **Yb–** *E* where the only Yb^III^ receiving level lies at >10000 cm^–1^ energy gap below the ligand’s triplet state (Figure 6). Based on these observations, one can infer that while effective ligand singlet-to-triplet-to-metal EnT to the high-lying levels of Dy^III^ and Tb^III^ takes place, quickly depleting the singlet state and quenching fluorescence, competitive metal-to-ligand energy back transfer processes likely lead to energy dissipation through the population of dark states (Figure S76). The efficiency of such energy back transfer mechanism prevents a sufficient lifetime of the visible emissive levels of Tb^III^ and Dy^III^, as well as the population of the low-lying NIR emissive level of Dy^III^, accounting for the absence of any metal-centered luminescence, even if the ligand’s triplet state is relatively efficiently activated.

On the other hand, for NIR emitters (Nd^III^, Er^III^, Tm^III^, Yb^III^), where the energy gap between the ligand triplet level and the accepting RE excited states is large (>7000 cm^–1^), a linear increase of EnT with Z^2^ (Figure S75) emerges. Since ISC is dependent on Z^2^, we can conclude that the observed EnT efficiency is herein regulated by the rate of ligand’s triplet population. This Z^2^ trend is largely masked in visible emitters because the energy-gap effect dominates the photocycle, but it likely remains an underlying cofactor. Thus, while the kinetic behavior scales consistently with the Lewis acidity across the series, this trend is only an apparent one, emerging from the subset of emitting ions and not holding when diamagnetic species are considered (Figure S59). These observations indicate that the branching ratio populating the ligand triplet state(s) governs the dissociation kinetics, in line with the lower isomerization propensity of the triplet relative to that of the singlet manifold. Despite the fact no simple direct quantitative correlation could be drawn between the above**-**discussed photodissociation parameters and the ligand**–**to**–**metal ET efficiencies in emissive RE complexes, these results nonetheless represent further confirmation of the striking difference between the complexes undergoing, or not undergoing (**La–** *E*, **Y–** *E*, **Gd–** *E*, and **Lu–** *E*), ligand**–**to**–**metal EnT.

Based on these observations, the large differences observed in the photokinetic response of the **Yb–** *E* and **Lu–** *E* pair was even more remarkable considering the proximity of these metals in the lanthanide series, with a difference in their ionic radii of only 0.008 Å, less than 1% (Figure S64).^4^ Notably, among the most technologically relevant RE pairs, the use of emissive and nonemissive REs together is common, especially for optical applications. Indeed, the Eu^III^/Y^III^ pair is widely used in red phosphor lamps usually composed of a Y^III^_2_O_3_:Eu^III^ blend named YOX.^69,70^ Other commonly used blends consist of La^III^PO_4_:Ce^III^,Tb^III^ (LAP), (Ce^III^,Tb^III^)­MgAl_11_O_19_ (CAT), and (Gd^III^,Mg)­B_5_O_10_:Ce^III^,Tb^III^ (CBT) in green phosphors, and BaMgAl_10_O_17_:Eu^2+^ (BAM) in blue phosphors. For these pairs of **RE–** *E* complexes, the photodissociation rates were notably different, with the one of **Eu–** *E* being almost four times slower than that of **Y–** *E* and the one of **Tb–** *E* being two times slower than that of **Gd–** *E*.^69^ The proximity in ionic radii for these pairs of REs usually prevents easy separation using common separation methods. Nevertheless, we suggest that a separation strategy based on the potential modification of the RE coordination environment following photodissociation, and governed by their optical characteristics, may provide a preliminary basis for improved separations.^4,71^

## Conclusions

The release of RE^III^ ions through the irradiation of complexes containing a photosensitive acylhydrazonic ligand was investigated. The RE complexes were fully characterized in the solid state, revealing their neutral [RE^III^((*E*)**–**L^py^)_2_(NO_3_)_3_] (RE = La) or charged nature [RE^III^((*E*)**–**L^py^)_2_(NO_3_)_2_]­(NO_3_) (RE = Pr–Lu, and Y). Interestingly, the behavior of the cerium complex was intermediate between that of the lanthanum and praseodymium complexes, with two distinct complexes in the asymmetric unit, giving rise to a complex of the formula [Ce^III^((*E*)**–**L^py^)_2_(NO_3_)_3_]­[Ce^III^((*E*)**–**L^py^)_2_(NO_3_)_2_]­(NO_3_). The isomerization of the ligand was studied upon irradiation with a 395 nm commercial black light LED strip, confirming the quantitative isomerization from the *E* **–** to the *Z* **–**form. The capability of the ligand to photoisomerize in solution even when involved in metal complexation was confirmed and made the system tunable, from a RE complex to a RE­(NO_3_)_3_ and free *Z* **–**isomer mixture due to the inability of the latter to coordinate RE^III^ ions. Photokinetic experiments under constant irradiation were performed for the entire series of complexes through simultaneous irradiation and UV**–**vis spectra monitoring. Although the photodissociation was quantitative for all the complexes, the decrease in the absorption band related to the complexes clearly showed differences along the RE**–**series that could not be ascribed to differences in the RE^III^ ionic radii. The fastest photodissociation was observed for the free ligand and the complexes with nonemissive RE^III^ ions (**La–** *E*, **Y–** *E*, and **Lu–** *E*). Conversely, for all emissive RE complexes, slower *E* **–**to**–** *Z* isomerization was observed, with **Yb–** *E* exhibiting an isomerization rate nine times slower than that of its neighbor containing Lu^III^. This phenomenon was ascribed to the inaccessibility of the metal excited stated in the case of the nonemissive complexes. In summary, we propose that RE**–**luminescence was possible for emissive RE complexes, along with additional nonradiative decay paths, causing a decrease in the photodissociation rate due to the effective population of triplet states of the ligand that are less reactive toward isomerization. Conversely, ISC is significantly slowed down or hindered in complexes with nonemissive ions, resulting in faster isomerizations and photodissociations. We contend this work could open a window on the separation of REs using alternative separation methods based on their different optical properties, such as photodissociation.

## Supplementary Material



## Notes

Experimental section, single-crystal X-ray structural characterization, ligand and complexes isomerization studies, UV–vis experiments, photophysical characterization, and DFT computations (PDF)
