# Supplementary material for: Metal-Dependent Photodissociation of Hydrazone Photoswitches from Rare-Earth Complexes
Source: J Am Chem Soc. 2026 Apr 9;148(15):16422–32. doi: 10.1021/jacs.6c03443 (PMC13107456; doi:10.1021/jacs.6c03443)
Supplement: Supplementary file 1 [file ja6c03443_si_001.pdf]

Supporting Information

Metal-Dependent Photodissociation of Hydrazone Photoswitches from Rare Earth Complexes

Matteo Melegari,[a] Randall K. Wilharm,[f] Rodolpho Nesta Silva,[d] Flavia Artizzu,[d] Angela Serpe,[b,c] Dirk H. Trauner,[e] Matteo Tegoni,[a] Eric J. Schelter,[f,g,h] and Luciano Marchiò[a]**

[a] Department of Chemistry, Life Sciences and Environmental Sustainability, University of Parma, Parco Area delle Scienze 17/A, 43124, Parma, Italy

[b] Department of Civil and Environmental Engineering and Architecture (DICAAR), and research unit of INSTM, University of Cagliari, Via Marengo 2, 09123 Cagliari, Italy

[c] Environmental Geology and Geoengineering Institute of the National Research Council (IGAG–CNR), Piazza d'Armi, 09123 Cagliari, Italy

[d] Department of Sustainable Development and Ecological Transition, University of Eastern Piedmont "A. Avogadro", Piazza S. Eusebio 5, 13100 Vercelli, Italy

[e] P. Roy and Diana T. Vagelos Laboratories, Department of Chemistry, University of Pennsylvania, 231 South 34th Street, Philadelphia, PA 19104, USA

[f] Vagelos Laboratory for Energy Science and Technology, Department of Chemistry, University of Pennsylvania, Philadelphia, PA 19104, USA

[g] Department of Earth and Environmental Science, University of Pennsylvania, Philadelphia, PA 19104, USA

[h] Department of Chemical and Biomolecular Engineering, University of Pennsylvania, Philadelphia, PA 19104, USA

Contents

Experimental Section **3**
Syntheses 6
Characterizations 7

X–ray Diffraction Data **15**

Ligand Isomerization **39**

UV–Vis Titrations **43**

Complexes Isomerization **46**
Bistability Study 50

UV–Visible Illumination Kinetics Data **52**

Photophysical Characterization of RE–complexes **65**
Ligand–to–metal energy transfer efficiency. 71

DFT Computations **75**

Experimental Section

General measurements. All reagents and solvents were purchased from Sigma–Aldrich and used without further purification. NMR experiments were performed on either a Brüker Avance 400 MHz instrument at 298 K and chemical shifts were reported in ppm relative to tetramethylsilane. ESI–MS measurements were carried out using a Waters Acquity Ultra Performance LC with Waters Acquity SQ Detector and with ESI interface. The data were collected in positive and negative ion modes, in the region of 100–2000 *m/z* at a flow rate of 0.20 mL/min. Infrared spectra were recorded by a Perkin Elmer Spectrum Two spectrometer in the 4000–400 cm^{-1} interval. Elemental analyses (CHN) were performed on Thermo Fischer Scientific FlashSmart analyzer (sample mass 2–3 mg). UV–Visible spectra were recorded on a Lambda 465 (PerkinElmer) UV–Visible spectrophotometer, equipped with a diode array detector. Samples were measured at 25°C inside quartz or plastic cuvettes of 10 cm optical path length in the spectral range 250–500 nm. The dissociation constants of the complexes were calculated using the HypSpec2014 software.[1] Commercial light sources were used for the photoisomerization experiment: 1) λ_1: 395 nm photoreactor built wrapping a commercial strip of 300 blue LEDs (chip SMD2835–300 ip20, 12V, 24W) on the inside of a crystallizing dish (15 cm in diameter); 2) λ_2: 365 nm, UVA radiation from a common TLC lamp; 3) λ_3: 254 nm, UVC radiation from the same TLC lamp.

UV–Visible Illumination kinetics. UV–Visible spectra were recorded using a Varian Cary 50 Bio UV–Visible Spectrophotometer. Photo–switching was achieved using a monochromator light source for varying wavelengths. The monochromator was pointed downward directly on top of the cuvette and held still through a hole in the cap of the cuvette. Different wavelengths of light were used to illuminate the solution. Wavelength scans were performed recording the absorption spectra (250–500 nm) at varying illumination wavelength (from 440 nm to 300 nm with 20 nm step size) after 10 min of irradiation at the respective wavelength to reach steady PSS. The reference spectra of the compound before isomerization, labeled as "dark", was recorded illuminating the sample with a 600 nm wavelength, which could not trigger the isomerization reaction. The measurements were performed at 25°C without stirring the solutions inside the cuvettes.

Actinometry and Quantum Yield Determination. The quantum yield of photoisomerization was determined using 390 nm LED (Roithner LaserTechnik VL390-5-15, 3.2-4.2 V, 8.1-11.5 mW) irradiation, monitoring with UV-Vis absportion spectroscopy. Molar photon flux of the 390 nm LED apparatus was determined by potassium ferroxilate actinomentery.[2, 3] The set of 3 LEDs were positioned directly at the opening of the cuvette for all actinometric and quantum yield determination with identical clamping, positioning, and ambient light. Irradiation of 1 mL of an aqueous 0.15 M $K_3[Fe(C_2O_4)_3]$ solution in 0.05 M H_2SO_4 in a cuvette was performed for 10 s with magnetic stirring. To this solution was added 0.175 mL of an aqueous solution containing 0.00555 M 1,10-phenanthroline , 2.74 M sodium acetate, and 0.5 M H_2SO_4. This mixture was allowed to equilibrate in complete darkness for 1 h. The developed irradiated solution and an identically prepared non-irradiated solution were measured by UV-Vis spectroscopy to calculate the molar photon flux of the LED apparatus (I_0 = 1.439×10^{-5} E s^{-1}).

Samples of ***E*-L**^{py} or the rare earth complexes were prepared and irradiated with the same apparatus. Samples were irradiated for set time points, then removed from the LED apparatus to be measured by UV-Vis absorption spectroscopy, and repeated until no change in absorption was observed. Effective quantum yields (for the systems under equilibrium) were calculated using Equation 1 where $\frac{dA}{dt}$ is the slope at the inflection point of a fitted Boltzmann sigmoidal curve and the extinction coefficient of the *E* isomer (ε^{390}) to determine the rate of photoisomerization, *V* is the volume of the sample (0.001 L), and *f* is the fraction of absorbed photons at the inflection point calculated from the measured absorbance.

$$\Phi_{E\rightarrow Z}^{390} = -\frac{dA}{dt} \cdot \frac{V}{\varepsilon^{390} \cdot I_0 \cdot f} \qquad (1)$$

Photoluminescence (PL) Measurements. Photoluminescence measurements were performed using an Edinburgh FLS1000 spectrophotometer equipped with a PMT–900 Detector 200–870 nm and PMT–1700 in Liquid Nitrogen Cooled Housing, 300–1700 nm. Steady–state PL spectra were acquired using a 450 W Xenon Lamp. Appropriate optical filters were used, and steady–state emission data were corrected for the instrumental spectral response. All PL measurements were recorded at room temperature. Solid–state samples were put between quartz plates (Starna cuvette type 20/C/Q/0.2). Pulsed excitation for time–resolved measurements was provided by 375 nm pulsed diode laser operating at a repetition rate of 20 MHz (**Pr–*E***) or a 5W microsecond flash white light lamp (repetition rate 100 Hz).

Diffuse reflectance spectra were recorded on a Cary 5000 Series UV–Vis–NIR spectrometer equipped with an external DRA integrating sphere module in the 200–2000 nm range, using Teflon tape as blank measurement.

Single crystal X–ray Diffraction. Single crystal data were collected at 200 K with a Bruker (US) D8 Venture diffractometer equipped with a Photon III detector, using a microfocus radiation source (Mo Kα: λ = 0.71073 Å; Cu Kα: λ = 1.54184 Å). The intensity data were integrated from several series of exposure frames covering the sphere of reciprocal space. Data reductions were performed with APEX5. Absorption corrections were applied using the program SADABS.[4] The structures were solved by intrinsic phasing with the program SHELXT.[5] Fourier analysis and refinement were performed by the full–matrix least–squares methods based on F^2 using SHELXL–2017 implemented in Olex2 software (version 1.5).[6] Non–H atoms were refined anisotropically. H atoms were placed in their calculated positions except for the ones bonded to oxygen and nitrogen atoms, in OH and NH, respectively, that were found and refined from their electron density on the residual electron density map. In the structure of **La–*E*** two methanol molecules were found disordered over two positions each. The first one (O1S) was refined with site occupancy factors of 0.5, each, while the second one (O2S) was refined with site occupancy factors of 0.75 and 0.25, respectively. In the structure of **Ce–*E*** the uncoordinated nitrate ion was found disordered over two positions that were refined with site occupancy factors of 0.52 and 0.48, respectively. In the structure of **Pr–*E*** the uncoordinated nitrate ion was found disordered over two positions that were refined with site occupancy factors of 0.68 and 0.32, respectively. The water molecule present in the structure was refined with site occupancy of 0.3. In the structure of **Sm–*E*** The uncoordinated nitrate ion was found disordered over two positions that were refined with site occupancy factors of 0.64 and 0.36, respectively. In the structure of **Eu–*E*** the uncoordinated nitrate ion was found disordered over two positions that were refined with site occupancy factors of 0.73 and 0.27, respectively. In the structure of **Gd–*E*** the uncoordinated nitrate ion was found disordered over two positions that were refined with site occupancy factors of 0.54 and 0.46, respectively. In the structure of **Tb–*E*** The uncoordinated nitrate ion was found disordered over two positions that were refined with site occupancy factors of 0.59 and 0.41, respectively. In the structure of **Dy–*E*** the uncoordinated nitrate ion was found disordered over two positions that were refined with site occupancy factors of 0.60 and 0.40, respectively. In the structure of **Ho–*E*** the uncoordinated nitrate ions were both found disordered over two positions each. The first one (N17) was refined with site occupancy factors of 0.63 and 0.37, respectively, while the second one (N110) was refined with site occupancy factors of 0.53 and 0.47, respectively. In the structure of **Tm–*E*** the uncoordinated nitrate ions were both found disordered over two positions each. The first one (N2) was refined with site occupancy factors of 0.62 and 0.38, respectively, while the second one (N1) was refined with site occupancy factors of 0.50 and 0.50, respectively. In the structure of **Yb–*E*** the uncoordinated nitrate ion and one of the phenyl rings of the ligands were found disordered over two positions that were refined with site occupancy factors of 0.5 and 0.5, respectively. One water molecule present in the structure was refined with site occupancy of 0.5. In the structure of **Lu–*E*** one of the non-coordinated nitrate ions (N19) was found disordered over two positions that were refined with site occupancy factors of 0.57and 0.43, respectively. In the structure of **Y–*E*** The uncoordinated nitrate ion was found disordered over two positions that were refined with site occupancy factors of 0.52 and 0.48, respectively. The pyridyl ring of one of the ligands was found disordered over two positions that were refined with site occupancy factors of 0.52 and 0.48, respectively. The pyridyl and phenyl ring of the

other ligand molecule were found disordered over two positions each, that were refined with site occupancy factors of 0.52 and 0.48, respectively.

Buried and free volume calculations were performed using the online program SambVca.[7] Shape analysis to determine the coordination geometry of the metal in the complexes was performed using the SHAPE 2.1 program.[8] Graphical material was prepared with the Mercury 2021 program.[9]

Computational results. Density functional theory (DFT) calculations were performed using the Gaussian 16 program suite.[10] Molecular geometries in the ground state were optimized without symmetry constraints, starting from the X–ray geometries of **(*E*)–L^{py}**. Inputs for the *Z*–isomers were obtained by rotation around the C=N of 180°. The PBE0 density functional, suitable for the calculation of excited state energies, was used along with def2–TZVP basis set.[11, 12] The calculations were performed using the polarizable continuum model (CPCM) using the dielectric constant of MeOH to address implicit solvation effects in solution.[13] Frequency calculations were carried out at the same level of theory to confirm the nature of stationary points as minima on the potential energy surface. Thermochemical values (electronic energy and thermal free energy correction) were calculated at 298.15 K and 1.0 atm. Time Dependent (TD–DFT) calculations were performed on the minimum energy configurations to compute the excitation energies. The computations were performed on the same level of theory as the optimization calculations. The lowest energy transitions (wavelength higher than 240 nm) are reported along with the excited state energies (eV), associated wavelengths (nm), oscillator strengths, number of the orbitals involved, and related coefficients. The 3D representation of the electron probability distributions of the orbitals involved in the S_1 transition were visualized using GaussView 6. The simulated UV–Visible spectra based on TD–DFT calculations (25 singlet or 25 triplet states) were simulated with Gaussview 6 and the extracted data were plotted using OriginPro 2019.

Syntheses

Synthesis of (*E*)–L^{py}

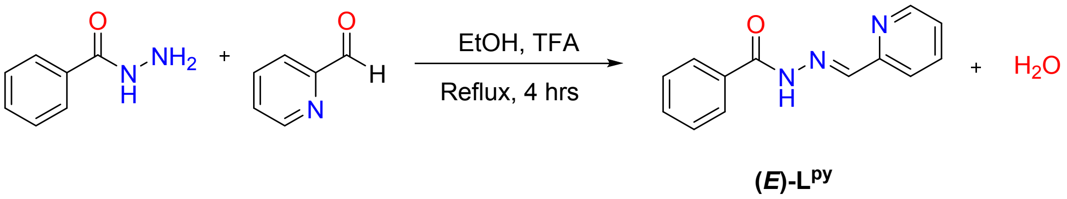

Scheme S1. Synthetic path for the synthesis of **(*E*)–L^{py}**.

The synthesis of the ligand was adapted from literature.[14] Benzohydrazide (544 mg; 4.0 mmol) was dissolved in 7 mL of EtOH. Pyridine–2–carboxaldehyde (424 mg; 4.0 mmol) was added to the mixture together with trifluoroacetic acid (catalytic amount, *c.a.* 3 drops). The mixture was refluxed for 4 hours and then the solution was layered with Et_2O and cooled in the fridge overnight. A consistent amount of white crystalline material was formed then filtered and washed with a cold 1:2 mixture of EtOH:Et_2O and dried under vacuum.

Synthesis of RE–*E* complexes

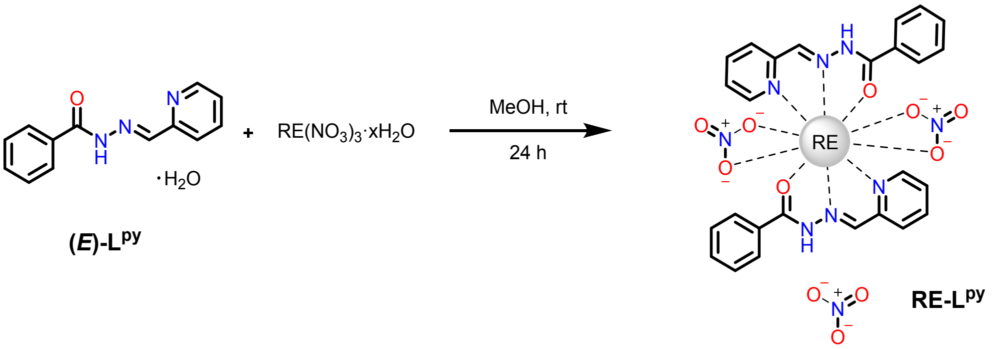

Scheme S2. Synthetic path for the synthesis of **RE–*E*** complexes.

The synthesis of the complexes was adapted from literature.[14] **(*E*)–L^{py}**$\cdot H_2O$ (80 mg; 0.33 mmol) was dissolved in 1 mL of MeOH. $RE(NO_3)_3\cdot xH_2O$ (RE = Y, La–Lu except Pm; x = 5 for Y, La–Sm and Gd, and 6 for Eu, Tb–Lu) (0.165 mmol) was dissolved in 1 mL of MeOH and slowly added to the ligand solution at room temperature. The reaction mixture was kept at room temperature without stirring for a few days. After that period, the formation of crystals is observed. The solid was crushed, filtered and washed two times with 1 mL of MeOH.

Characterizations

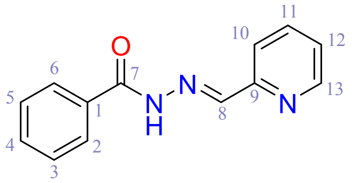

(*E*)–N'–(pyridin–2–ylmethylene)benzohydrazide ((*E*)–L^py^). Yield: 701 mg of white crystalline solid (72 %). ^{1}H–NMR (400 MHz, MeOH–d_4): 8.60 (d, J = 5.0 Hz, 1H, H_{13}), 8.43 (s, 1H, H_8), 8.35 (d, J = 8.0 Hz, 1H, H_{10}), 7.99 (d, J = 7.2 Hz, 2H, $H_{6,2}$), 7.90 (t, J = 7.6 Hz, 1H, H_{11}), 7.62 (t, J = 7.6 Hz, 1H, H_4), 7.53 (t, J = 8.4 Hz, 2H, $H_{5,3}$), 7.44 (t, J = 6 Hz, 1H, H_{12}). ESI–MS (ACN): calculated for $[M + H]^+$ m/z = 226.26. Found: 226.15. Elemental analysis calculated % (found %) for $C_{13}H_{11}N_3O{\cdot}H_2O$ (M = 243.27 g mol^{-1}) (%): C, 64.19 (64.41); H, 5.39 (5.21); N, 17.27 (17.51). FTIR (ATR, ν_{max}/cm^{-1}): 3457 (m, stretching NH); 3183 (w); 3066 (w); 3005 (m); 1656 (m, stretching C=O); 1560 (s); 1468 (m); 1435 (m); 1351 (m); 1280 (s); 1144 (m); 1073 (m); 997 (m); 962 (w); 954 (m); 921 (m); 844 (w); 803 (m); 776 (m); 746 (m); 700 (s); 680 (s); 625 (s); 502 (m); 426 (m). Crystals suitable for X–ray diffraction were grown from slow evaporation of an ethanol solution and the structure matched that present in the CSD (deposition number 142156).[15] ^{13}C NMR (400 MHz, MeOH–d_4): 150.8 (C_{13}), 149.9 (C_8), 139.3 (C_{11}), 134.2 (C_4), 130.4 ($C_{5,3}$), 129.5 ($C_{6,2}$), 126.7 (C_{12}), 122.9 (C_{10}).

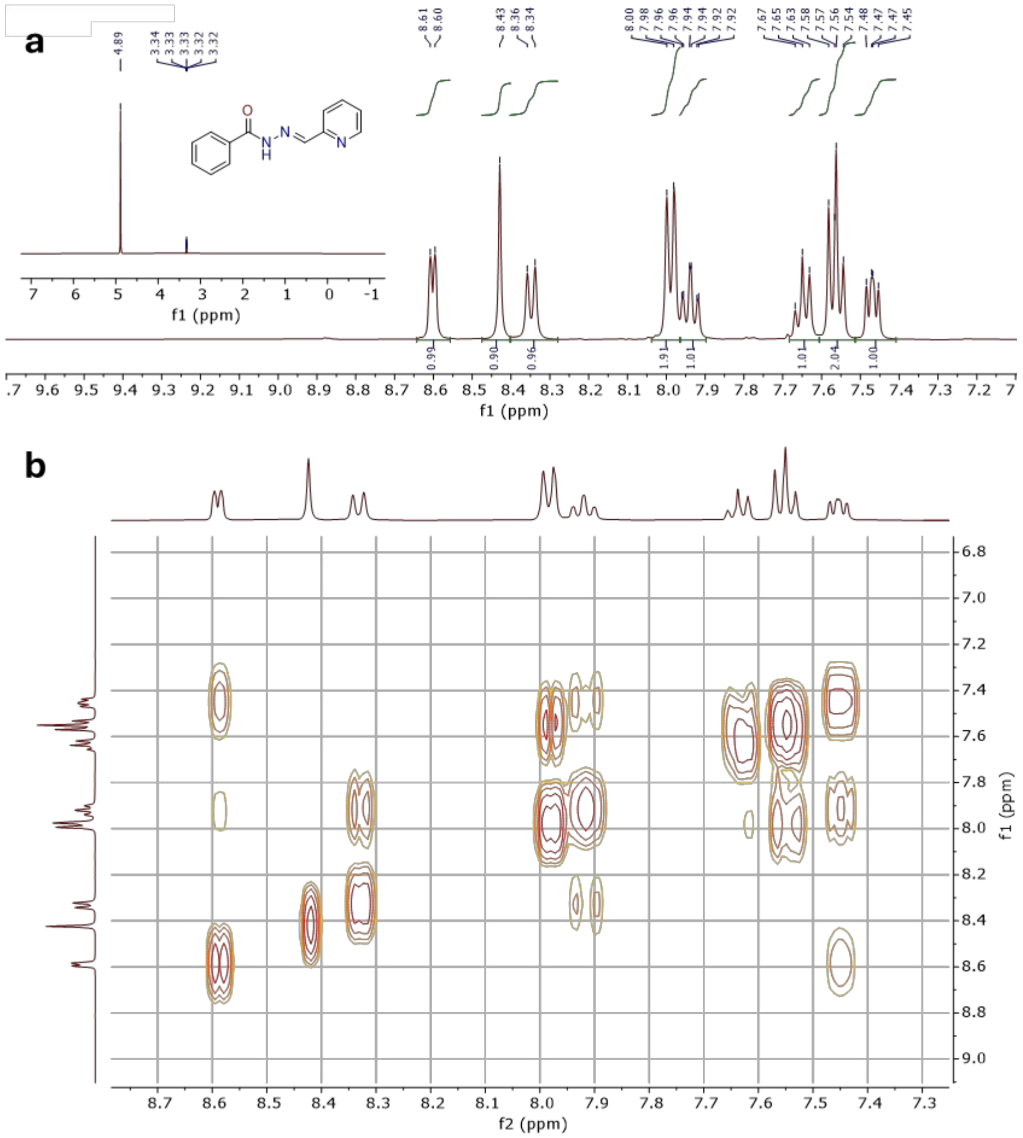

Figure S1. a) ^{1}H NMR (400 MHz, MeOH–d_4) and b) COSY NMR spectra of **(*E*)–L^py^**.

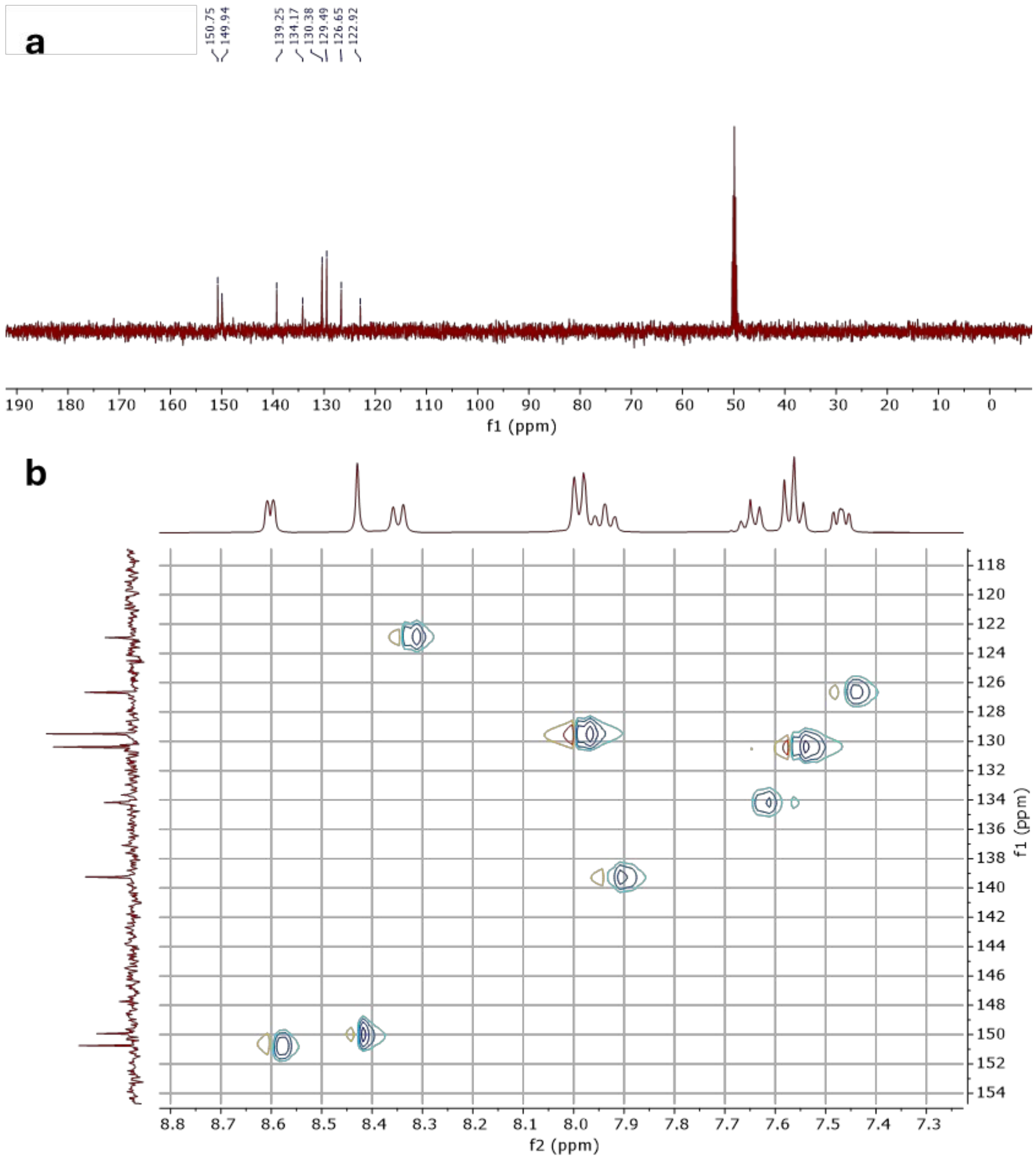

Figure S2. a) ^{13}C NMR (400 MHz, MeOH–d_4) and b) HSQC NMR (400 MHz, MeOH–d_4) spectra of **(*E*)–L^{py}**.

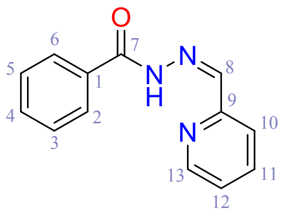

(*Z*)–N'–(pyridin–2–ylmethylene)benzohydrazide ((*Z*)–L^{py}). ^{1}H NMR (400 MHz, MeOH–*d*$_4$): 8.87 (d, J = 8 Hz, 1H, H$_{13}$), 8.04 (td, J1 = 8 Hz, 1H, H$_{11}$), 8.01 (d, J = 8 Hz, 2H, H$_{2,6}$), 7.78 (d, J = 8 Hz, 1H, H$_{10}$), 7.69 (s, 1H, H$_8$), 7.60 (m, 4H, J = 8 Hz, H$_{3,5}$, H$_4$, H$_{12}$). FTIR (ATR, ν_{max}/cm^{-1}): 2963 (w), 2923 (w), 2851 (w), 1683 (s, C=O stretching), 1595 (m), 1578 (m), 1558 (m), 1527 (m), 1472 (m), 1423 (m), 1384 (m), 1300 (m), 1261 (s, C=O stretching), 1245 (m), 1183 (w), 1132 (m), 1101 (s), 1075 (m), 1050 (s), 1018 (m), 915 (m), 807 (m), 778 (s), 698 (s), 666 (m), 582 (s), 504 (m), 436 (w). ^{13}C NMR (400 MHz, MeOH–*d*$_4$): 150.5 (C$_{13}$), 142.1 (C$_8$), 140.6 (C$_{11}$), 134.7 (C$_4$), 131.1 (C$_{5,3}$), 129.5 (C$_{6,2}$), 128.8 (C$_{10}$), 127.0 (C$_{12}$).

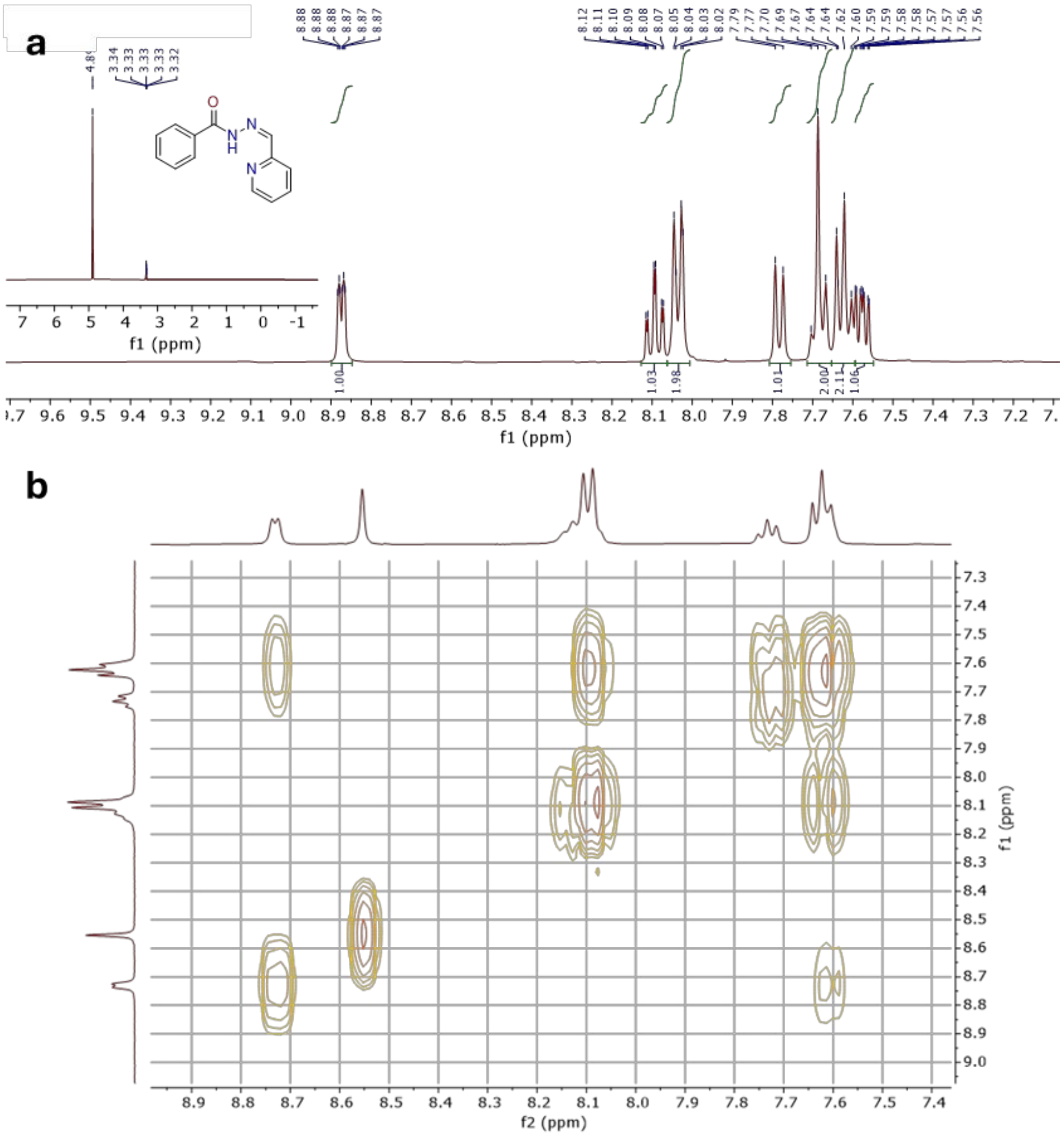

Figure S3. a) ^{1}H NMR (400 MHz, MeOH–*d*$_4$) and b) COSY NMR (400 MHz, MeOH–*d*$_4$) spectra of **(*Z*)–L^{py}**.

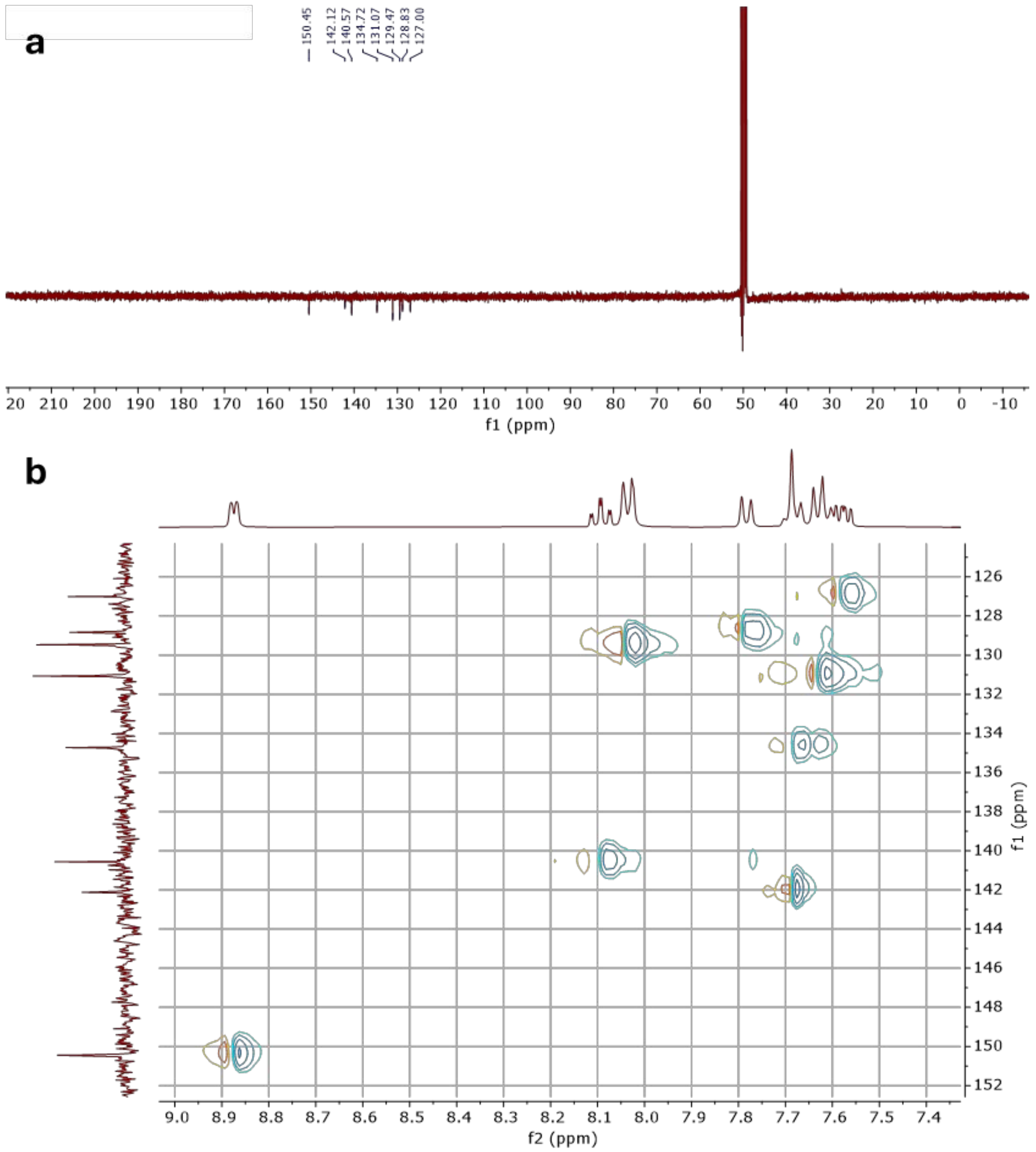

Figure S4. a) DEPT–135 (400 MHz, MeOH–d_4) and b) HSQC NMR (400 MHz, MeOH–d_4) spectra of **(*Z*)–L^{py}**.

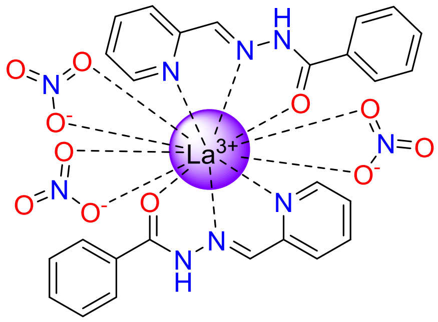

La–*E*. White solid. Yield: 100 mg, 76%. Elemental analysis calculated % (found %) for $La(C_{13}H_{11}N_3O)_2(NO_3)_3(H_2O)$ (M = 793.43 g mol^{-1}) (%): C, 38.91 (39.00); H, 3.14 (3.21); N, 15.70 (15.49). FTIR (ATR, ν_{max}/cm^{-1}): 3459 (w), 3191 (w, NH stretching), 3066 (w), 2293 (w), 2853 (w), 1631 (m, C=O stretching), 1601 (m, C=O stretching), 1568 (m, C=N stretching), 1435 (s), 1359 (s), 1310 (s), 1296 (s, nitrate stretching), 1228 (m), 1185 (m), 1142 (m), 1081 (m), 1042 (m), 1003 (m), 950 (m), 921 (m), 862 (w), 805 (m), 774 (m), 739 (m), 702 (s), 672 (m), 631 (m), 518 (m), 416 (m). ^{1}H NMR (400 MHz, MeOH–d_4): 8.77 (d, J = 4.8 Hz, 1H, H_{13}), 8.59 (s, 2H, H_8), 8.15–8.10 (m, 8H, $H_{2,6,10,11}$), 7.76 (t, J = 7.4 Hz, 2H, H_{12}), 7.64 (m, 6H, $H_{3,4,5}$). Crystals suitable for single crystal X–ray diffraction were grown from the reaction mixture in MeOH corresponding to $[La^{III}((E)–L^{py})_2(NO_3)_3]·2(MeOH)$.

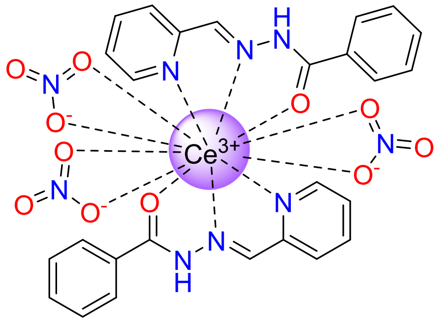

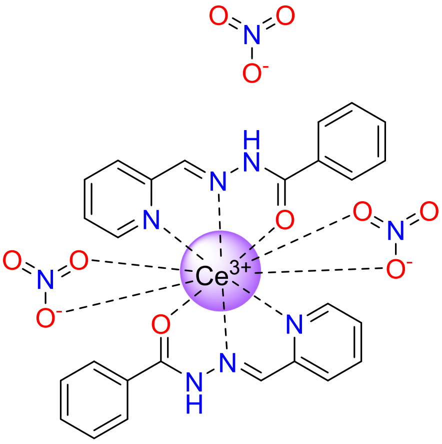

Ce–*E*. Yellow solid. Yield: 101 mg, 77%. Elemental analysis calculated % (found %) for $Ce(C_{13}H_{11}N_3O)_2(NO_3)_3(H_2O)$ (M = 794.64 g mol^{-1}) (%): C, 39.30 (39.34); H, 3.04 (3.03); N, 15.86 (15.50). FTIR (ATR, ν_{max}/cm^{-1}): 3447 (w), 3195 (w, N–H stretching), 3070 (w), 2999 (w), 2851 (w), 1630 (m, C=O stretching), 1622 (m, C=O stretching), 1612 (m, C=O stretching), 1601 (m, C=O stretching), 1566 (m, C=N stretching), 1435 (s), 1355 (m), 1298 (s, nitrate stretching), 1275 (s, nitrate stretching), 1228 (m), 1149 (m), 1085 (m), 1036 (m), 1022 (m), 1005 (m), 948 (m), 919 (m), 864 (m), 815 (m), 776 (m), 747 (m), 731 (m), 696 (m), 629 (m), 518 (m), 418(m). Crystals suitable for single crystal X–ray diffraction were grown from the reaction mixture in MeOH, corresponding to $[Ce^{III}((E)–L^{py})_2(NO_3)_3][Ce^{III}((E)–L^{py})_2(NO_3)_2](NO_3)·(MeOH)$.

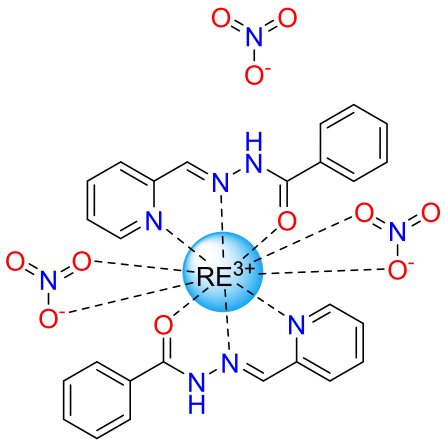

Pr–*E*. Greenish solid. Yield: 93 mg, 73%. Elemental analysis calculated % (found %) for $Pr(C_{13}H_{11}N_3O)_2(NO_3)_3$ (M = 777.41 g mol^{-1}) (%): C, 40.17 (40.03); H, 2.85 (2.82); N, 16.22 (15.87). FTIR (ATR, ν_{max}/cm^{-1}): 3189 (w, N–H stretching), 3068 (w), 2999 (w), 2958 (w), 2857 (w), 1605 (m, C=O stretching), 1600 (m, C=O stretching), 1566 (s, C=N stretching), 1496 (m), 1474 (m), 1443 (m), 1368 (m), 1331 (m), 1304 (s), 1271 (s, nitrate stretching), 1228 (m), 1189 (m), 1147 (m), 1112 (m), 1089 (m), 1054 (m), 1026 (m), 1005 (m), 977 (m), 950 (m), 917 (m), 862 (m), 815 (m), 782 (s), 729 (m), 688 (s), 672 (m), 633 (m), 528 (m), 426 (m), 412 (m). Crystals suitable for single crystal X–ray diffraction were grown from the reaction mixture in MeOH corresponding to $[Pr^{III}((E)–L^{py})_2(NO_3)_2](NO_3)\cdot 0.25(H_2O)$.

Nd–*E*. Pink solid. Yield: 102 mg, 79.1%. Elemental analysis calculated for $Nd(C_{13}H_{11}N_3O)_2(NO_3)_3$ (M = 780.75 g mol^{-1}) (%): C, 40.00 (40.04); H, 2.84 (2.83); N, 16.15 (15.98). FTIR (ATR, ν_{max}/cm^{-1}): 3193 (w, N–H stretching), 3063 (w), 3025 (w), 2861 (w), 1607 (m, C=O stretching), 1600 (m, C=O stretching), 1565 (s, C=N stretching), 1504 (m), 1476 (s), 1448 (m), 1371 (m), 1327 (m), 1303 (m), 1271 (m, nitrate stretching), 1231 (m), 1188 (w), 1152 (m), 1113 (m), 1090 (m), 1056 (m), 1031 (m), 1026 (m), 1009 (m), 1000 (w), 694 (w), 917 (m), 867 (w), 823 (w), 815 (m), 800 (w), 781 (m), 750 (w), 733 (m), 688 (s), 671 (m), 633 (m), 528 (m), 520 (m), 503 (m), 416 (m). Crystals suitable for single crystal X–ray diffraction were grown by stratification of Et_2O over a MeOH solution of the compound corresponding to $[Nd^{III}((E)–L^{py})_2(NO_3)_2](NO_3)$.

Sm–*E*. Off white solid. Yield: 94 mg, 72%. Elemental analysis calculated % (found %) for $Sm(C_{13}H_{11}N_3O)_2(NO_3)_3$ (M = 786.87 g mol^{-1}) (%): C, 39.69 (39.82); H, 2.82 (2.79); N, 16.02 (15.73). FTIR (ATR, ν_{max}/cm^{-1}): 3193 (w, N–H stretching), 3068 (w), 3019 (w), 2999 (w), 2862 (w), 1618 (m, C=O stretching), 1608 (m, C=O stretching), 1599 (m, C=O stretching), 1564 (m, C=N stretching), 1499 (m), 1474 (m), 1447 (m), 1370 (m), 1327 (s), 1302 (s), 1275 (s, nitrate stretching), 1228 (m), 1187 (m), 1151 (s), 1089 (m), 1056 (m), 1026 (m), 1007 (m), 950 (m), 917 (m), 864 (m), 813 (m), 782 (m), 749 (m), 686 (s), 633 (m), 528 (m), 416 (m). Crystals suitable for single crystal X–ray diffraction were grown from the reaction mixture in MeOH, corresponding to $[Sm^{III}((E)–L^{py})_2(NO_3)_2](NO_3)$.

Eu–*E*. Off white solid. Yield: 111 mg, 85%. Elemental analysis calculated % (found %) for $Eu(C_{13}H_{11}N_3O)_2(NO_3)_3$ (M = 788.47 g mol^{-1}) (%): C, 39.61 (39.82); H, 2.81 (2.74); N, 15.99 (15.72). FTIR (ATR, ν_{max}/cm^{-1}): 3185 (w, N–H stretching), 3066 (w), 3021 (w), 2964 (w), 2857 (w), 1618 (m, C=O stretching), 1608 (m, C=O stretching), 1600 (m, C=O stretching), 1566 (m, C=N stretching), 1501 (m), 1476 (m), 1368 (m), 1327 (s), 1304 (s), 1273 (s, nitrate stretching), 1230 (m), 1189 (m), 1149 (m), 1114 (m), 1093 (m), 1026 (m), 1007 (m), 948 (m), 919 (m), 866 (m), 813 (m), 780 (w), 735 (m), 688 (s), 633 (m), 530 (m), 418 (m). Crystals suitable for single crystal X–ray diffraction were grown from the reaction mixture in MeOH, corresponding to $[Eu^{III}((E)–L^{py})_2(NO_3)_2](NO_3)$.

Gd–*E*. Off white solid. Yield: 106 mg, 81%. Elemental analysis calculated % (found %) for $Gd(C_{13}H_{11}N_3O)_2(NO_3)_3$ (M = 793.76 g mol^{-1}) (%): C, 39.34 (39.47); H, 2.79 (2.74); N, 15.88 (15.59). FTIR

(ATR, ν_{max}/cm^{-1}): 3197 (w, N–H stretching), 3066 (w), 3025 (w), 3003 (w), 2964 (w), 2857 (w), 1619 (m, C=O stretching), 1609 (m, C=O stretching), 1600 (m, C=O stretching), 1566 (s, C=N stretching), 1505 (m), 1478 (s), 1449 (m), 1372 (m), 1327 (m), 1304 (s), 1275 (s, nitrate stretching), 1230 (m), 1185 (m), 1153 (m), 1114 (m), 1091 (m), 1056 (m), 1026 (m), 1009 (m), 948 (m), 919 (m), 813 (m), 780 (m), 737 (m), 688 (s), 633 (m), 530 (m), 520 (m), 418 (m). Crystals suitable for single crystal X–ray diffraction were grown from the reaction mixture in MeOH, corresponding to $[Gd^{III}((E)–L^{py})_2(NO_3)_2](NO_3)$.

Tb–*E*. Off white solid. Yield: 107 mg, 81%. Elemental analysis calculated % (found %) for $Tb(C_{13}H_{11}N_3O)_2(NO_3)_3$ (M = 795.43 g mol^{-1}) (%): C, 39.26 (39.45); H, 2.79 (2.76); N, 15.85 (15.71). FTIR (ATR, ν_{max}/cm^{-1}): 3193 (w, N–H stretching), 3066 (w), 3021 (w), 2966 (w), 2860 (w), 1619 (m, C=O stretching), 1609 (m, C=O stretching), 1600 (m, C=O stretching), 1566 (s, C=N stretching), 1505 (m), 1476 (s), 1449 (m), 1370 (m), 1329 (s), 1302 (s), 1277 (s, nitrate stretching), 1230 (m), 1187 (m), 1153 (m), 1112 (m), 1095 (m), 1028 (m), 1009 (m), 948 (m), 919 (m), 864 (w), 813 (m), 780 (s), 739 (m), 688 (s), 633 (m), 533 (m), 520 (m), 418 (m). Crystals suitable for single crystal X–ray diffraction were grown from the reaction mixture in MeOH, corresponding to $[Tb^{III}((E)–L^{py})_2(NO_3)_2](NO_3)$.

Dy–*E*. Yellow solid. Yield: 106 mg, 81 %. Elemental analysis calculated for $Dy(C_{13}H_{11}N_3O)_2(NO_3)_3$ (M = 799.01 g mol^{-1}) (%): C, 39.08 (39.16); H, 2.76 (2.78); N, 15.78 (15.56). FTIR (ATR, ν_{max}/cm^{-1}): 3193 (w, N–H stretching), 3063 (w), 3025 (w), 2861 (w), 1620 (m, C=O stretching), 1609 (m, C=O stretching), 1600 (m, C=O stretching), 1568 (s, C=N stretching), 1504 (m), 1476 (s), 1448 (m), 1371 (m), 1327 (m), 1304 (m), 1294 (m), 1271 (s, nitrate stretching), 1231 (m), 1188 (w), 1152 (m), 1113 (m), 1090 (m), 1056 (m), 1034 (m), 1028 (m), 1009 (m), 1000 (w), 694 (w), 920 (m), 863 (w), 823 (w), 814 (m), 800 (w), 781 (m), 750 (w), 740 (m), 688 (s), 671 (m), 633 (m), 534 (m), 522 (m), 503 (m), 420 (m). Crystals suitable for single crystal X–ray diffraction were grown by stratification of Et_2O over a MeOH solution of the compound corresponding to $[Dy^{III}((E)–L^{py})_2(NO_3)_2](NO_3)$.

Y–*E*. White solid. Yield: 93 mg, 74%. Elemental analysis calculated % (found %) for $Y(C_{13}H_{11}N_3O)_2(NO_3)_3(H_2O)_2$ (M = 761.44 g mol^{-1}) (%): C, 41.01 (41.15); H, 3.44 (3.24); N, 16.56 (6.33). FTIR (ATR, ν_{max}/cm^{-1}): 3191 (w, N–H stretching), 2984 (w), 2900 (w), 1619 (m, C=O stretching), 1610 (m, C=O stretching), 1600 (m, C=O stretching), 1568 (m, C=N stretching), 1466 (m), 1404 (m), 1363 (m), 1320 (s), 1292 (s, nitrate stretching), 1226 (m), 1183 (m), 1149 (m), 1087 (m), 1032 (m), 1009 (m), 952 (m), 917 (m), 815 (m), 776 (m), 741 (m), 704 (m), 688 (m), 539 (m), 524 (m), 420 (m). ^{1}H NMR (400 MHz, MeOH–d_4): 8.60 (d, J = 4.0 Hz, 1H, H_3), 8.50 (s, 1H, H_8), 8.18 (s, 1H, H_{10}), 8.08 (s, 1H, H_{11}), 8.03 (d, J = 8.4 Hz, 2H, $H_{2,6}$), 7.66 (t, J = 7.2 Hz, 1H, H_4), 7.59 (s, 1H, H_{12}), 7.56 (t, J = 8.0 Hz, 2H, $H_{3,5}$). Crystals suitable for single crystal X–ray diffraction were grown from the reaction mixture in MeOH corresponding to $[Y^{III}((E)–L^{py})_2(NO_3)_2](NO_3)$.

Ho–*E*. Off white solid. Yield: 102 mg, 74%. Elemental analysis calculated % (found %) for $Ho(C_{13}H_{11}N_3O)_2(NO_3)_3(H_2O)_2$ (M = 837.47 g mol^{-1}) (%): C, 37.29 (37.59); H, 3.13 (2.93); N, 15.05 (14.94). FTIR (ATR, ν_{max}/cm^{-1}): 3189 (w, N–H stretching), 3062 (w), 2972 (w), 2849 (w), 1619 (m, C=O stretching), 1609 (m, C=O stretching), 1600 (m, C=O stretching), 1568 (m, C=N stretching), 1464 (m), 1406 (m), 1363 (m), 1318 (m), 1290 (s, nitrate stretching), 1226 (m), 1185 (m), 1149 (m), 1114 (m), 1091(m), 1032 (m), 1009 (m), 950 (m), 919 (m), 813 (m), 776 (s), 741 (m), 688 (s), 635 (m), 539 (m), 522 (m), 508 (m), 422 (m). Crystals suitable for single crystal X–ray diffraction were grown from the reaction mixture in MeOH, corresponding to $[Ho^{III}((E)–L^{py})_2(NO_3)_2](NO_3)$.

Er–*E*. White solid. Yield: 99 mg, 73%. Elemental analysis calculated % (found %) for $Er(C_{13}H_{11}N_3O)_2(NO_3)_3(H_2O)$ (M = 821.76 g mol^{-1}) (%): C, 38.00 (37.78); H, 2.94 (2.86); N, 15.34 (15.09). FTIR (ATR, ν_{max}/cm^{-1}): 3189 (w, N–H stretching), 3066 (w), 2974 (w), 2843 (w), 1619 (m, C=O stretching), 1610 (m, C=O stretching), 1601 (m, C=O stretching), 1568 (m, C=N stretching), 1470 (m), 1363 (m), 1290 (s, nitrate stretching), 1228 (m), 1189 (m), 1151 (m), 1114 (m), 1095 (m), 1032 (m), 948 (m), 919 (m), 813 (m),

776 (m–s), 741 (m), 688 (s), 633 (m–s), 537 (m), 522 (m), 422 (m). The crystal structure of the compound was already present in the CSD (ref code 141461), corresponding to $[Er^{III}((E)–L^{py})_2(NO_3)_2](NO_3)·1.5(H_2O)$.[13]

Tm–*E*. After 3 days no crystals were observed, so the reaction mixture was left to evaporate to half of the initial volume. After that, colorless crystals form in a few more days. White solid. Yield: 81 mg, 59%. Elemental analysis calculated % (found %) for $Tm(C_{13}H_{11}N_3O)_2(NO_3)_3(H_2O)$ (M = 823.76 g mol^{-1}) (%): C, 37.92 (38.13); H, 2.94 (2.81) N, 15.31 (15.07). FTIR (ATR, ν_{max}/cm^{-1}): 3191 (w, N–H stretching), 3068 (w), 2970 (w), 2853 (w), 1616 (m, C=O stretching), 1610 (m, C=O stretching), 1600 (m, C=O stretching), 1566 (m, C=N stretching), 1476 (m), 1363 (m), 1284 (s, nitrate stretching), 1226 (m), 1185 (m), 1153 (m), 1116 (m), 1095 (m), 1034 (m), 1009 (m), 948 (m), 919 (m), 813 (m), 778 (m), 743 (m), 686 (s), 633 (m), 537 (m), 522 (m), 422 (m). Crystals suitable for single crystal X–ray diffraction were grown from the reaction mixture in MeOH, corresponding to $[Tm^{III}((E)–L^{py})_2(NO_3)_2](NO_3)$.

Yb–*E*. After 3 days no crystals were observed, so the reaction mixture was left to evaporate to half of the initial volume. After that, colorless crystals form in a few more days. White solid. Yield: 68 mg, 49%. Elemental analysis calculated % (found %) for $Yb(C_{13}H_{11}N_3O)_2(NO_3)_3(H_2O)(CH_3OH)_{0.5}$ (M = 843.58 g mol^{-1}) (%): C, 37.73 (37.41); H, 3.11 (2.88); N, 14.93 (14.66). FTIR (ATR, ν_{max}/cm^{-1}): 3191 (w, N–H stretching), 3064 (w), 2974 (w), 2849 (w), 1618 (m, C=O stretching), 1610 (m, C=O stretching), 1601 (m, C=O stretching), 1568 (m, C=N stretching), 1470 (m), 1443 (m), 1411 (m), 1363 (m), 1320 (s), 1290 (s, nitrate stretching), 1228 (m), 1185 (m), 1151 (m), 1116 (m), 1091 (m), 1034 (m), 1009 (m), 954 (m), 919 (m), 813 (m), 776 (m), 743 (m), 704 (m), 688 (m–s), 633 (m), 541 (m), 522 (m), 442 (m), 422 (m). Crystals suitable for single crystal X–ray diffraction were grown from the reaction mixture in MeOH, corresponding to $[Yb^{III}((E)–L^{py})_2(NO_3)_2](NO_3)·1.5(H_2O)$.

Lu–*E*. After 3 days no crystals were observed, so the reaction mixture was left to evaporate to half of the initial volume. After that, colorless crystals form in a few more days. White solid. Yield: 56 mg, 40%. Elemental analysis calculated % (found %) for $Lu(C_{13}H_{11}N_3O)_2(NO_3)_3(H_2O)_{1.5}$ (M = 838.50 g mol^{-1}) (%): C, 37.24 (37.29); H, 3.00 (2.89); N, 15.03 (14.83). FTIR (ATR, ν_{max}/cm^{-1}): 3194 (w, N–H stretching), 3066 (w), 2972 (w), 2853 (w), 1619 (m, C=O stretching), 1611 (m, C=O stretching), 1601 (m, C=O stretching), 1569 (s, C=N stretching), 1466 (m), 1443 (m), 1404 (m), 1363 (s), 1323 (s), 1294 (s, nitrate stretching), 1226 (m), 1187 (m), 1149 (m), 1114 (m), 1095 (m), 1034 (s), 1007 (m), 948 (m), 919 (m), 813 (m), 776 (m), 688 (s), 633 (m), 541 (m), 522 (m), 422 (m). ^{1}H NMR (400 MHz, MeOH–d_4): 8.67 (s, 1H, H_3), 8.59 (s, 1H, H_8), 8.16 (m, 2H, $H_{10,11}$), 8.03 (s, 2H, $H_{2,6}$), 7.70 (s, 1H, H_{12}), 7.69 (t, J = 7.2 Hz, 1H, H_4), 7.57 (t, J = 7.6 Hz, 2H, $H_{3,5}$). Crystals suitable for single crystal X–ray diffraction were grown from the reaction mixture in MeOH, corresponding to $[Lu^{III}((E)–L^{py})_2(NO_3)_2](NO_3)$.

X–ray Diffraction Data

Table S1. List of RE–*E* compounds and corresponding formula determined by SC–XRD.

Compound	Structure
**La–*E***	$[La^{III}((E)\text{–}L^{py})_2(NO_3)_3]\cdot 2(MeOH)$
**Ce–*E***	$[Ce^{III}((E)\text{–}L^{py})_2(NO_3)_3][Ce^{III}((E)\text{–}L^{py})_2(NO_3)_2](NO_3)\cdot(MeOH)$
**Ce–*E*·(acetone)**	[a]$[Ce^{III}((E)\text{–}L^{py})_2(NO_3)_3]\cdot 2(acetone)\cdot 2(H_2O)$
**Pr–*E***	$[Pr^{III}((E)\text{–}L^{py})_2(NO_3)_2](NO_3)\cdot 0.25(H_2O)$
**Nd–*E***	$[Nd^{III}((E)\text{–}L^{py})_2(NO_3)_2](NO_3)$
**Sm–*E***	$[Sm^{III}((E)\text{–}L^{py})_2(NO_3)_2](NO_3)$
**Eu–*E***	$[Eu^{III}((E)\text{–}L^{py})_2(NO_3)_2](NO_3)$
**Gd–*E***	$[Gd^{III}((E)\text{–}L^{py})_2(NO_3)_2](NO_3)$
**Tb–*E***	$[Tb^{III}((E)\text{–}L^{py})_2(NO_3)_2](NO_3)$
**Dy–*E***	$[Dy^{III}((E)\text{–}L^{py})_2(NO_3)_2](NO_3)$
**Y–*E***	$[Y^{III}((E)\text{–}L^{py})_2(NO_3)_2](NO_3)$
**Ho–*E***	$[Ho^{III}((E)\text{–}L^{py})_2(NO_3)_2](NO_3)$
**Er–*E***	[b]$[Er^{III}((E)\text{–}L^{py})_2(NO_3)_2](NO_3)\cdot 1.5(H_2O)$
**Tm–*E***	$[Tm^{III}((E)\text{–}L^{py})_2(NO_3)_2](NO_3)$
**Yb–*E***	$[Yb^{III}((E)\text{–}L^{py})_2(NO_3)_2](NO_3)\cdot 1.5(H_2O)$
**Lu–*E***	$[Lu^{III}((E)\text{–}L^{py})_2(NO_3)_2](NO_3)$

[a]Structure already deposited in the CSD, ref code 131265. [b]Structure already deposited in the CSD, ref code 141461.

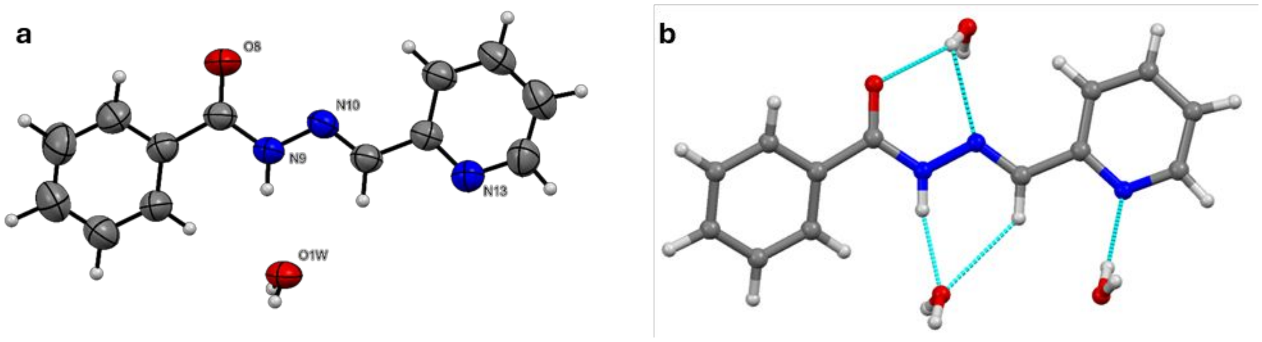

Figure S5. a) Molecular structure of ***(E)*–L^{py}** (already reported in the CSD, ref code 142156).[14] Thermal ellipsoids are depicted at the 30% probability level. Color codes: C, gray; H, white; O, red; N, blue. b) Crystal packing of the structure of ***(E)*–L^{py}** highlighting the intermolecular hydrogen bonds with the crystalized water molecule.

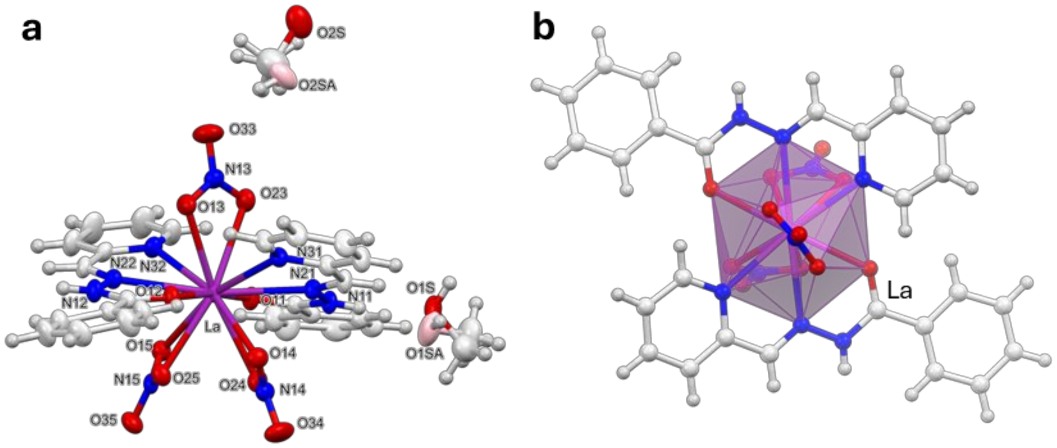

Figure S6. a) Molecular structure of [La^{III}((*E*)–L^{py})$_2$(NO$_3$)$_3$]·2(MeOH). Thermal ellipsoids are drawn at the 30% probability level. O atoms of the MeOH residue are depicted in red and pink to highlight the positional disorder. b) Polyhedral representation of the metal center in the structure of **La–*E***. Color codes: C, gray; H, white; O, red; N, blue; La, purple.

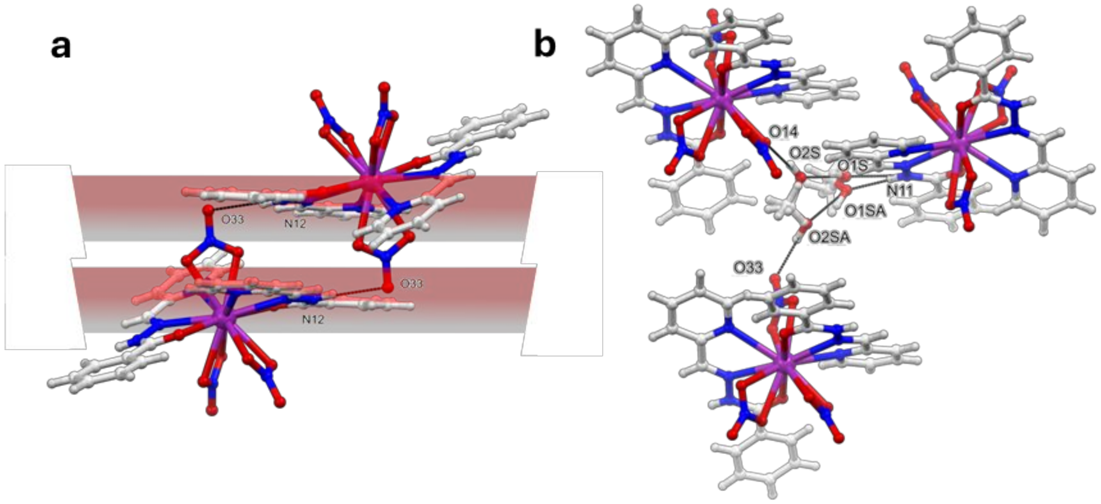

Figure S7. a) Crystal packing in the structure of **La–*E*** highlighting the hydrogen bonds between the hydrazonic N–H and nitrates (dotted lines). b) Stacking interactions are highlighted by the mean planes of adjacent ligands in red. Hydrogen bonds between the ligands and the solvent molecules.

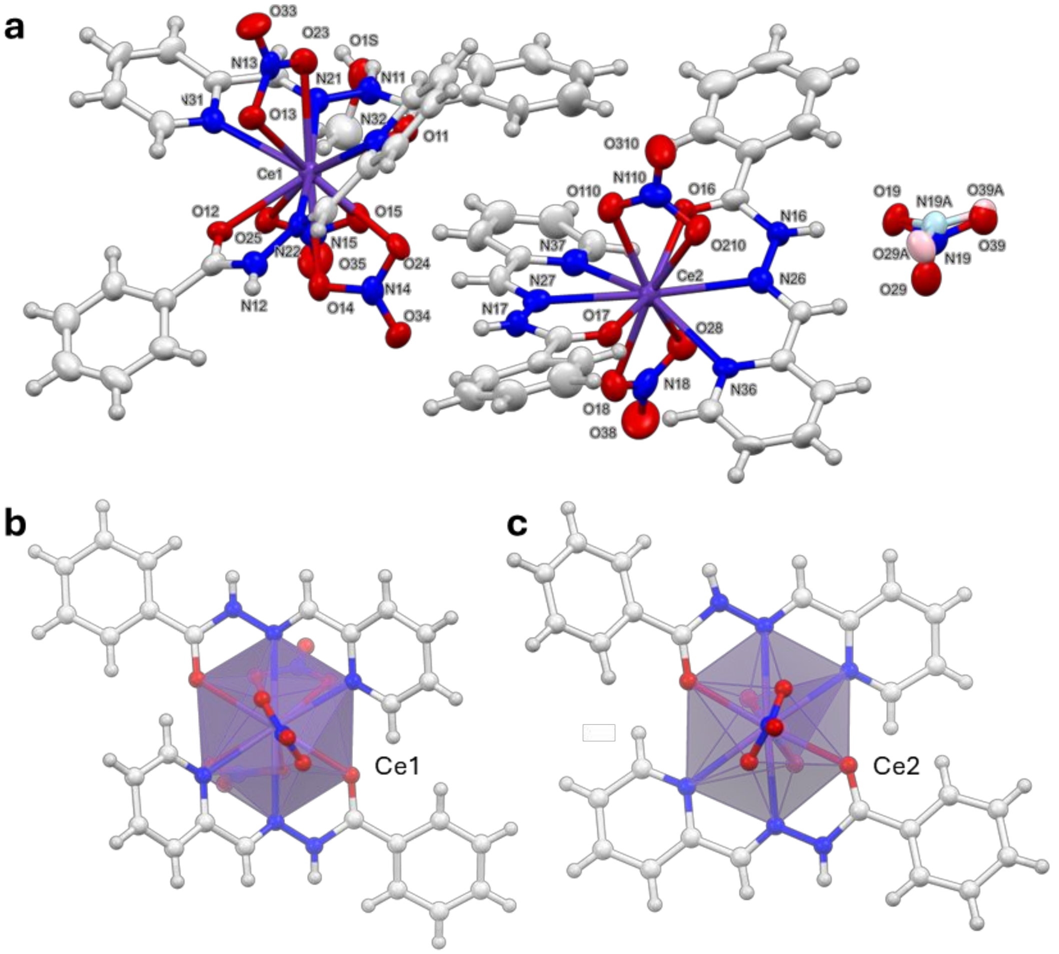

Figure S8. a) Molecular structure of [Ce^{III}((*E*)–L^{py})$_2$(NO$_3$)$_3$][Ce^{III}((*E*)–L^{py})$_2$(NO$_3$)$_2$](NO$_3$)·(MeOH). Thermal ellipsoids are drawn at the 30% probability level. Atoms of unbound nitrate residue are depicted in red and pink for O, and blue and light blue for N, to highlight the positional disorder. Polyhedral representation of the metal center in the structure of **Ce–*E*** for b) Ce1 and c) Ce2 atoms. Color codes: C, gray; H, white; O, red; N, blue; Ce, deep purple.

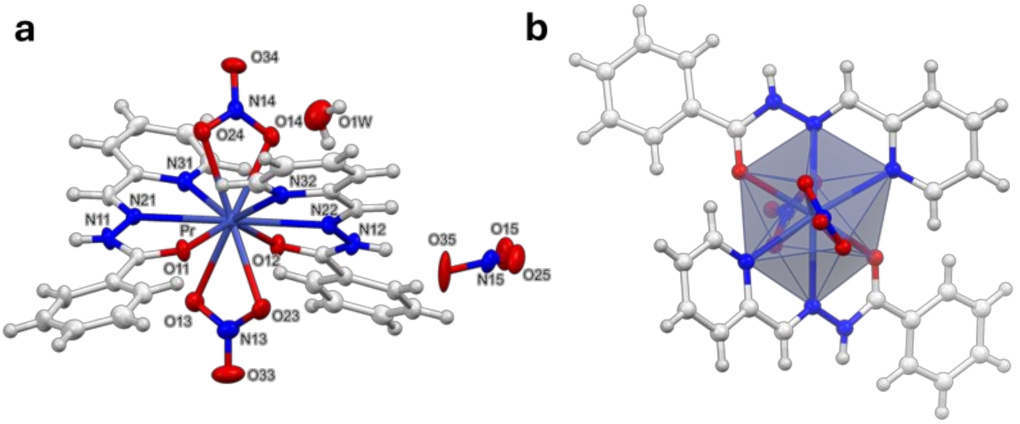

Figure S9. a) Molecular structure of [Pr^{III}((*E*)–L^{py})$_2$(NO$_3$)$_2$](NO$_3$)·0.25(H$_2$O). Thermal ellipsoids are drawn at the 30% probability level. b) Polyhedral representation of the metal center in the structure of **Pr–*E***. Color codes: C, gray; H, white; O, red; N, blue; Pr, indigo.

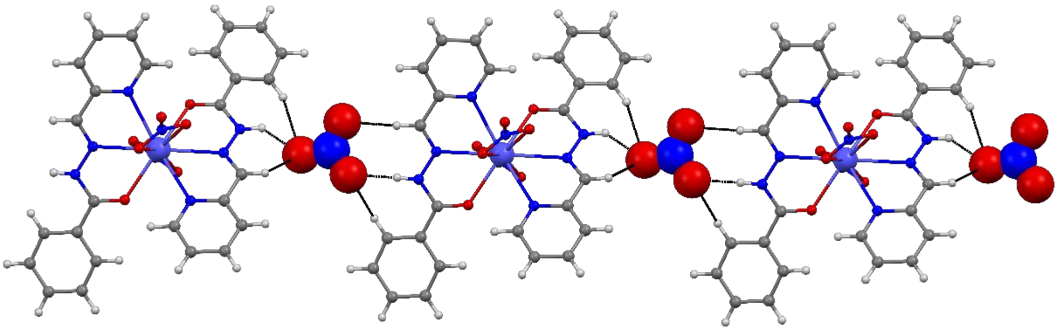

Figure S10. Crystal packing in the structure of **Pr–*E*** highlighting the hydrogen bonds between the hydrazonic N–H and nitrates (dotted lines).

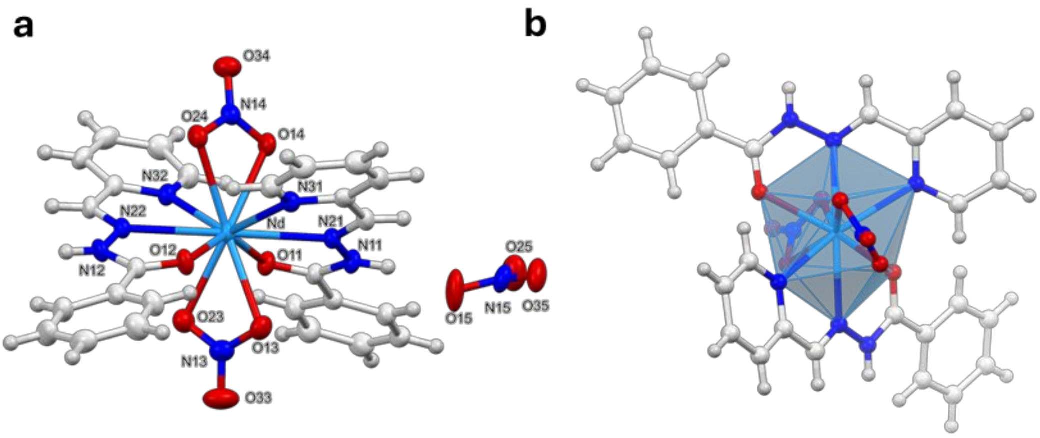

Figure S11. a) Molecular structure of [Nd^{III}((*E*)–L^{py})$_2$(NO$_3$)$_2$](NO$_3$). b) Thermal ellipsoids are drawn at the 30% probability level. Polyhedral representation of the metal center in the structure of **Nd–*E***. Color codes: C, gray; H, white; O, red; N, blue; Nd, blue.

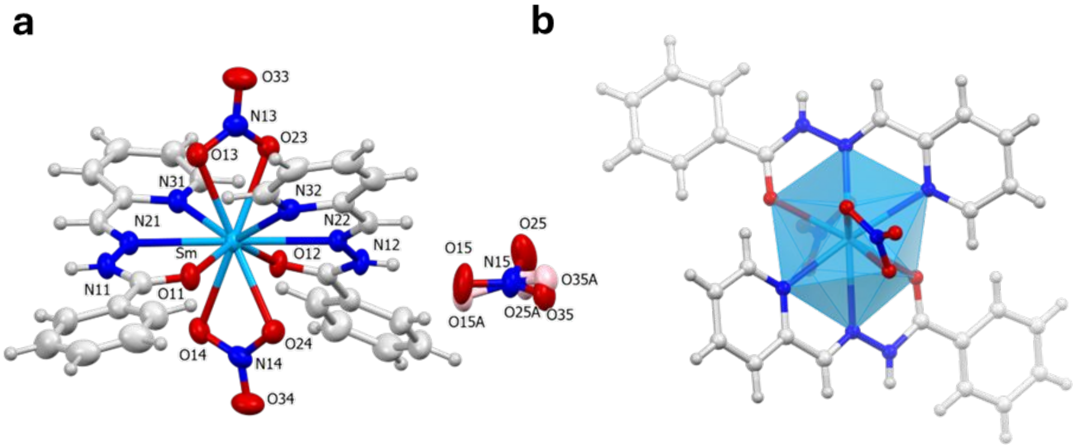

Figure S12. a) Molecular structure of [Sm^{III}((*E*)–L^{py})$_2$(NO$_3$)$_2$](NO$_3$). Thermal ellipsoids are drawn at the 30% probability level. Atoms of unbound nitrate residue are depicted in red and pink for O, and blue and light blue for N, to highlight the positional disorder. b) Polyhedral representation of the metal center in the structure of **Sm–*E***. Color codes: C, gray; H, white; O, red; N, blue; Nd, blue.

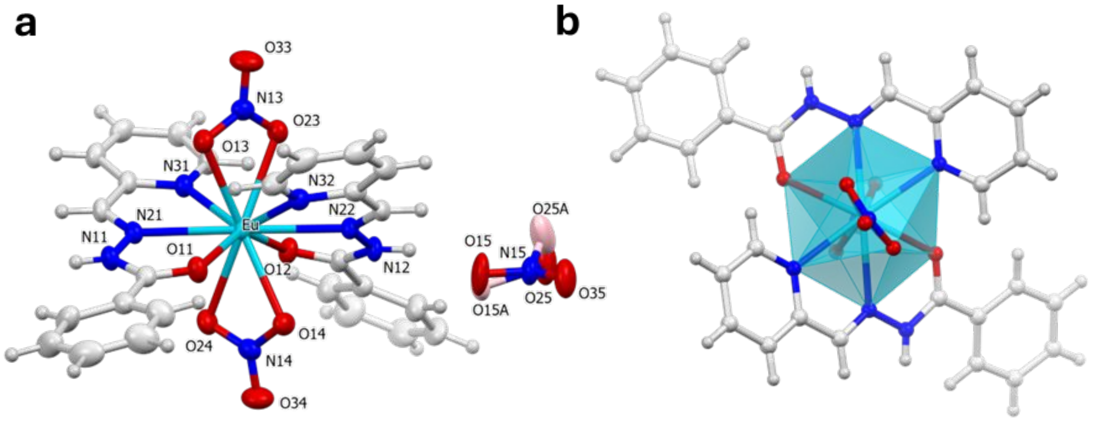

Figure S13. a) Molecular structure of [Eu^{III}((*E*)–L^{py})$_2$(NO$_3$)$_2$](NO$_3$). Thermal ellipsoids are drawn at the 30% probability level. Atoms of unbound nitrate residue are depicted in red and pink for O, and blue and light blue for N, to highlight the positional disorder. b) Polyhedral representation of the metal center in the structure of **Eu–*E***. Color codes: C, gray; H, white; O, red; N, blue; Nd, blue.

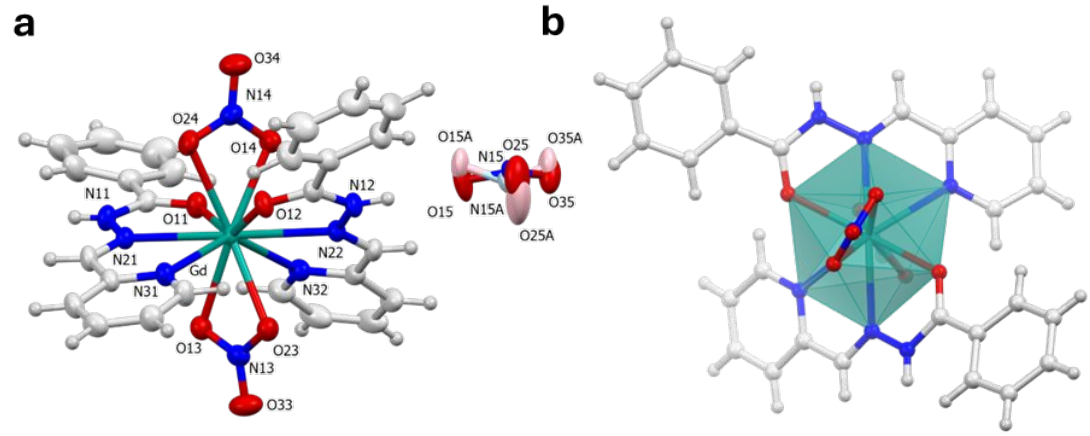

Figure S14. a) Molecular structure of [Gd^{III}((*E*)–L^{py})$_2$(NO$_3$)$_2$](NO$_3$). Thermal ellipsoids are drawn at the 30% probability level. Atoms of unbound nitrate residue are depicted in red and pink for O, and blue and light blue for N, to highlight the positional disorder. b) Polyhedral representation of the metal center in the structure of **Gd–*E*** b). Color codes: C, gray; H, white; O, red; N, blue; Nd, blue.

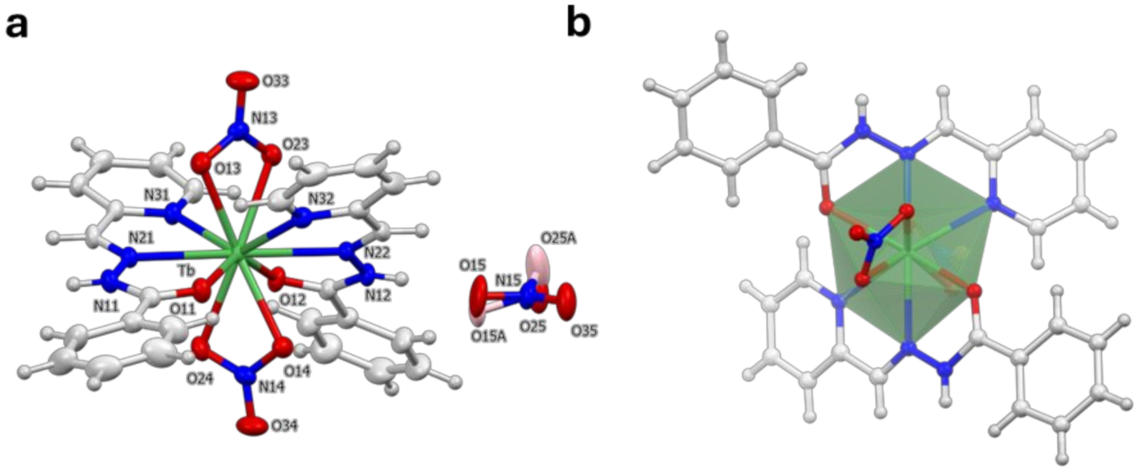

Figure S15. a) Molecular structure of [Tb^{III}((*E*)–L^{py})$_2$(NO$_3$)$_2$](NO$_3$). Thermal ellipsoids are drawn at the 30% probability level. Atoms of unbound nitrate residue are depicted in red and pink for O, and blue and light blue for N, to highlight the positional disorder. b) Polyhedral representation of the metal center in the structure of **Tb–*E***. Color codes: C, gray; H, white; O, red; N, blue; Nd, blue.

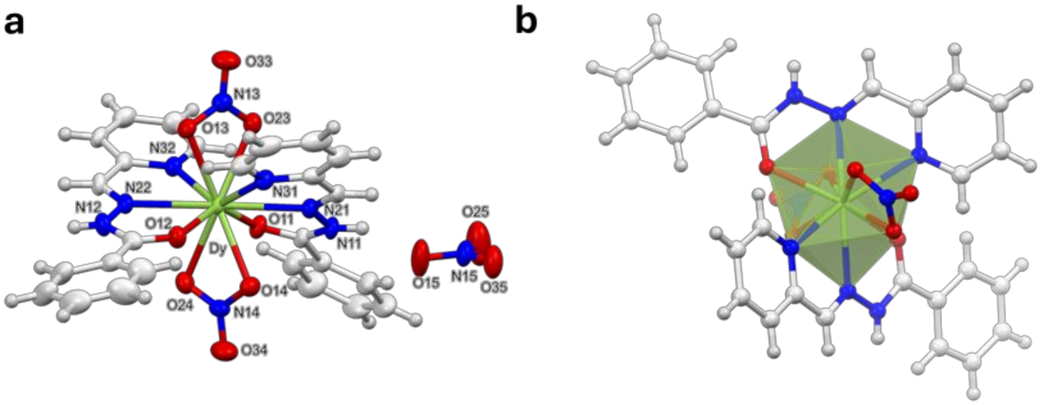

Figure S16. a) Molecular structure of $[Dy^{III}((E)\text{–}L^{py})_2(NO_3)_2](NO_3)$. Thermal ellipsoids are drawn at the 30% probability level. b) Polyhedral representation of the metal center in the structure of **Dy–*E*** b). Color codes: C, gray; H, white; O, red; N, blue; Dy, light green.

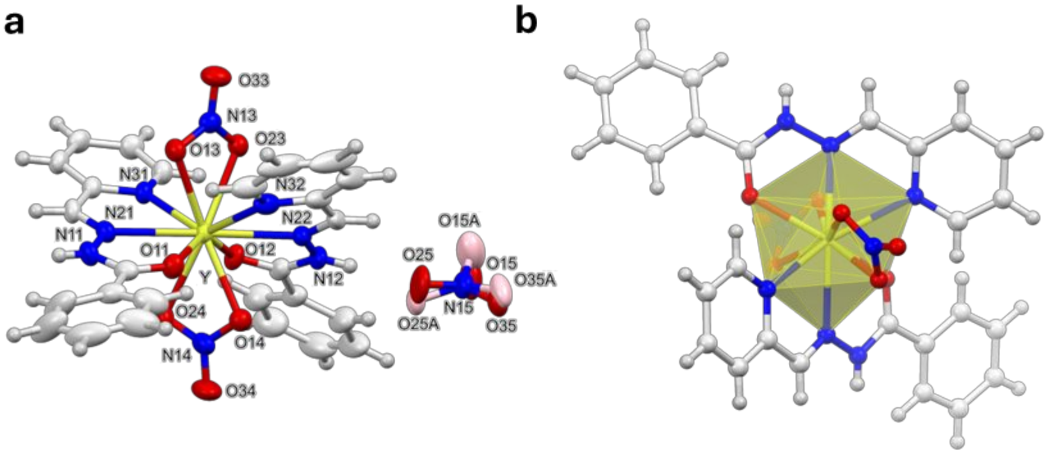

Figure S17. a) Molecular structure of $[Y^{III}((E)\text{–}L^{py})_2(NO_3)_2](NO_3)$. Thermal ellipsoids are drawn at the 30% probability level. b) Polyhedral representation of the metal center in the structure of **Y–*E***. Color codes: C, gray; H, white; O, red; N, blue; Y, lime green.

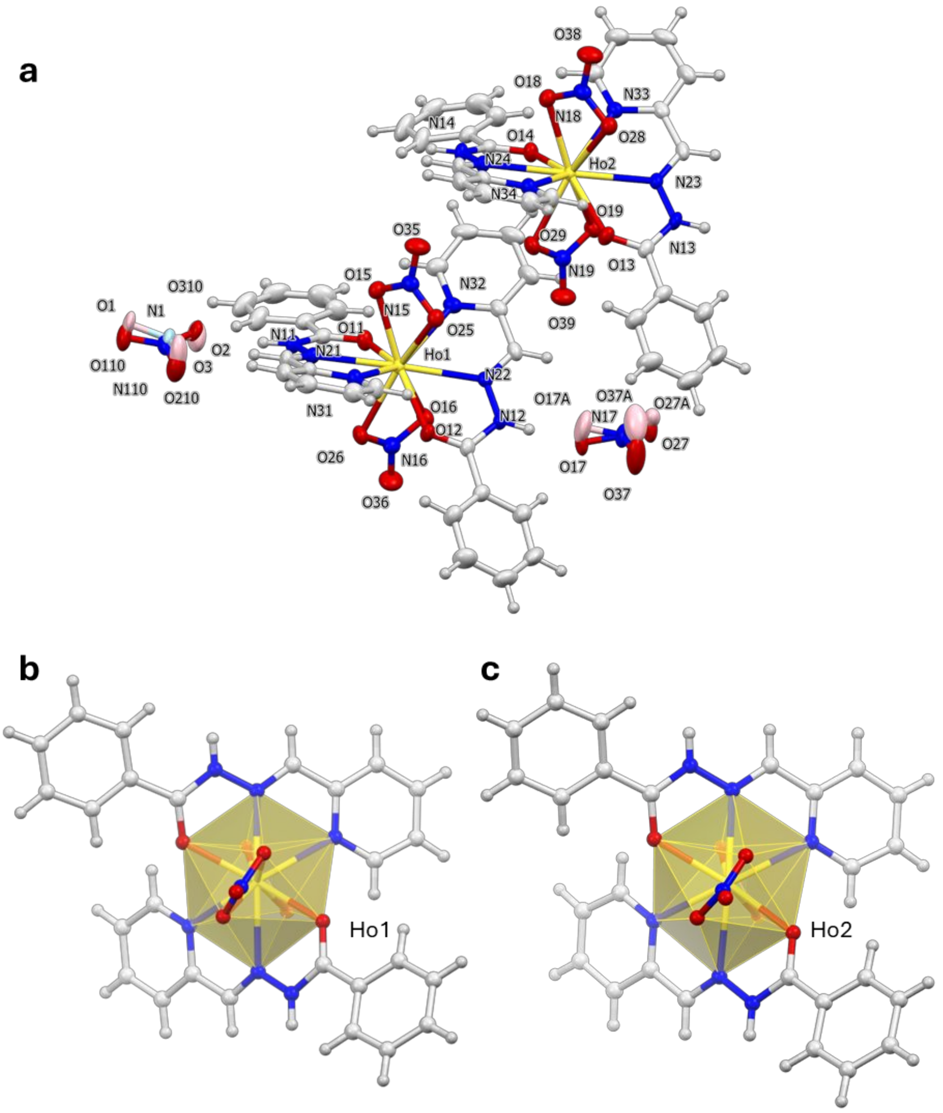

Figure S18. a) Molecular structure of [Ho^{III}((*E*)–L^{py})$_2$(NO_3)$_2$](NO_3). Thermal ellipsoids are drawn at the 30% probability level. Atoms of unbound nitrate residue are depicted in red and pink for O, and blue and light blue for N, to highlight the positional disorder. Polyhedral representation of the metal center in the structure of **Ho–*E*** for Ho1 b) and Ho2 c) atoms. Color codes: C, gray; H, white; O, red; N, blue; Lu, ruby.

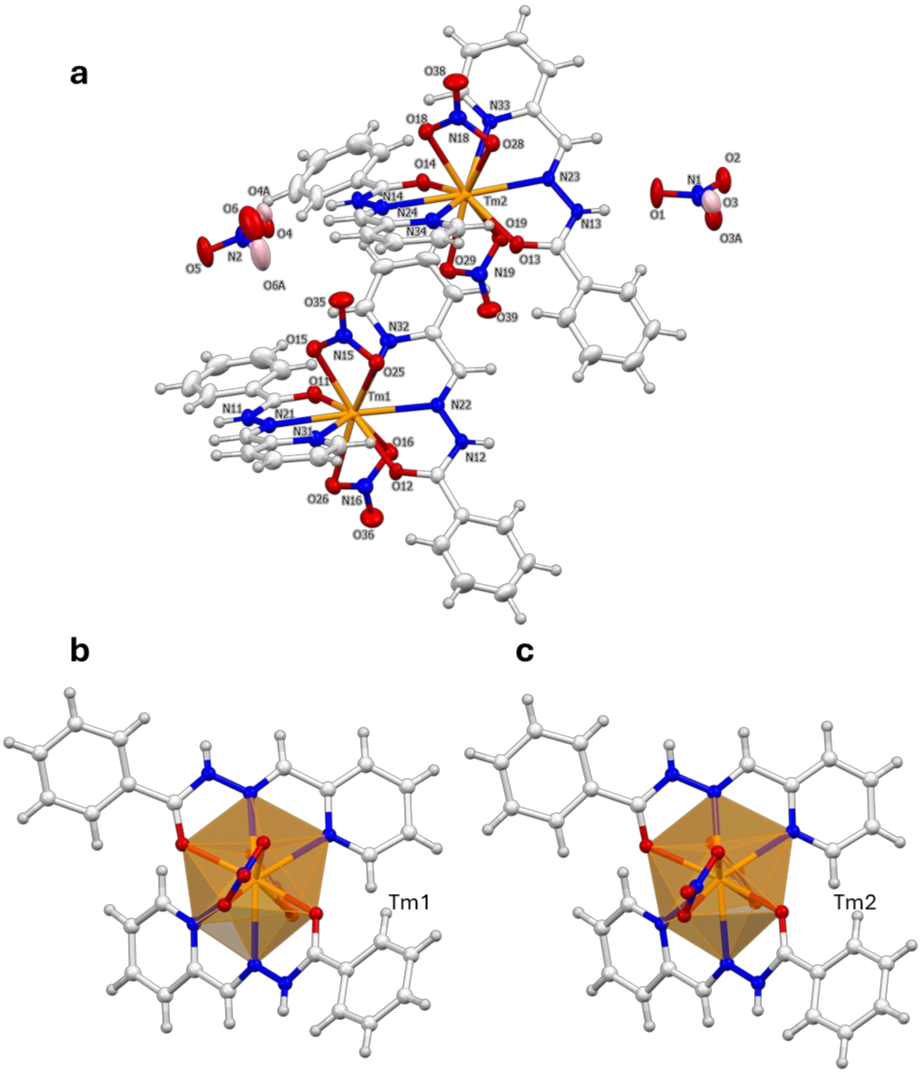

Figure S19. a) Molecular structure [Tm^{III}((*E*)–L^{py})$_2$(NO$_3$)$_2$](NO$_3$). Thermal ellipsoids are drawn at the 30% probability level. Atoms of unbound nitrate residue are depicted in red and pink for O, and blue and light blue for N, to highlight the positional disorder. b) Polyhedral representation of the metal center in the structure of **Yb–*E***. Color codes: C, gray; H, white; O, red; N, blue; Yb, red

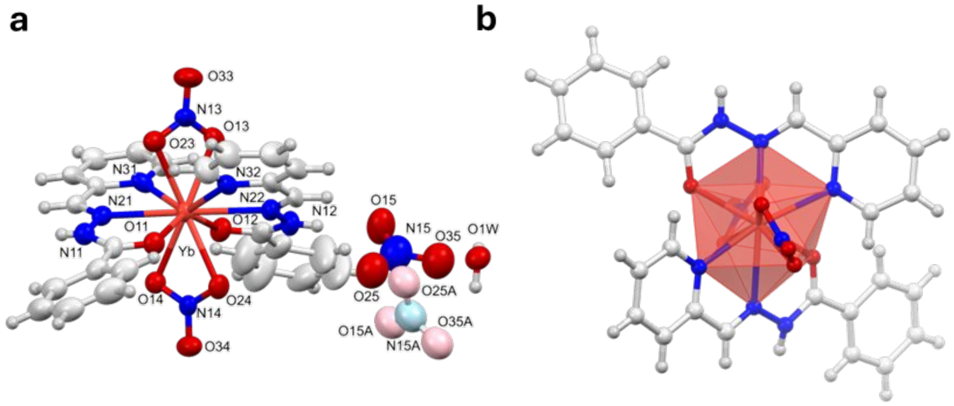

Figure S20. a) Molecular structure [Yb^{III}((*E*)–L^{py})$_2$(NO$_3$)$_2$](NO$_3$)·1.5(H$_2$O). Thermal ellipsoids are drawn at the 30% probability level. Atoms of unbound nitrate residue are depicted in red and pink for O, and blue and light blue for N, to highlight the positional disorder. b) Polyhedral representation of the metal center in the structure of **Yb–*E***. Color codes: C, gray; H, white; O, red; N, blue; Yb, red.

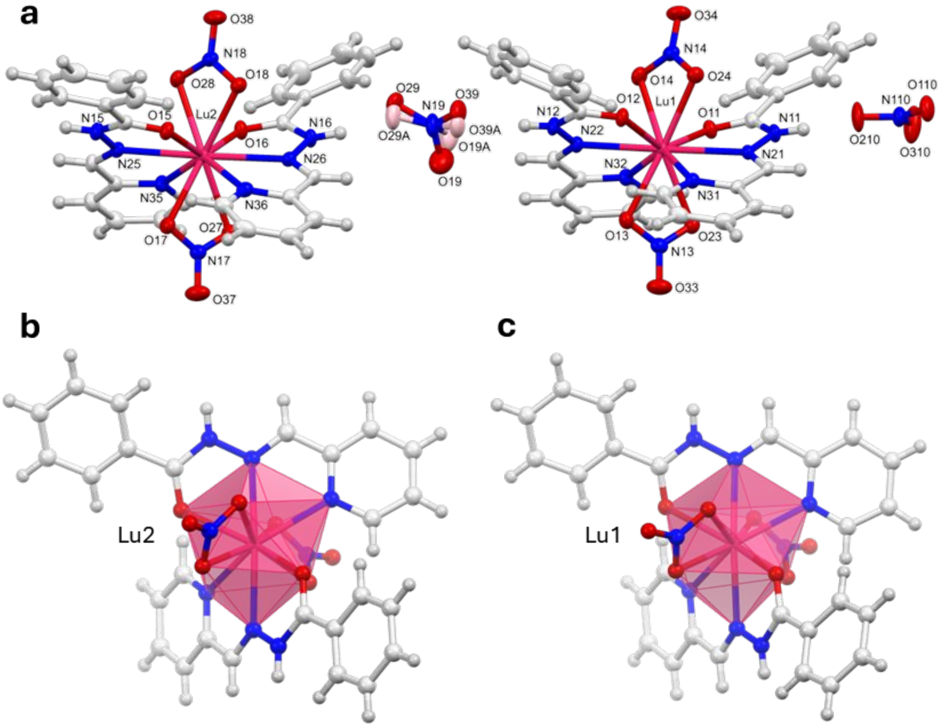

Figure S21. a) Molecular structure of $[Lu^{III}((E)\text{–}L^{py})_2(NO_3)_2](NO_3)$. Thermal ellipsoids are drawn at the 30% probability level. Atoms of unbound nitrate residue are depicted in red and pink for O, and blue and light blue for N, to highlight the positional disorder. Polyhedral representation of the metal center in the structure of **Lu–*E*** for Lu2 b) and Lu1 c) atoms. Color codes: C, gray; H, white; O, red; N, blue; Lu, ruby.

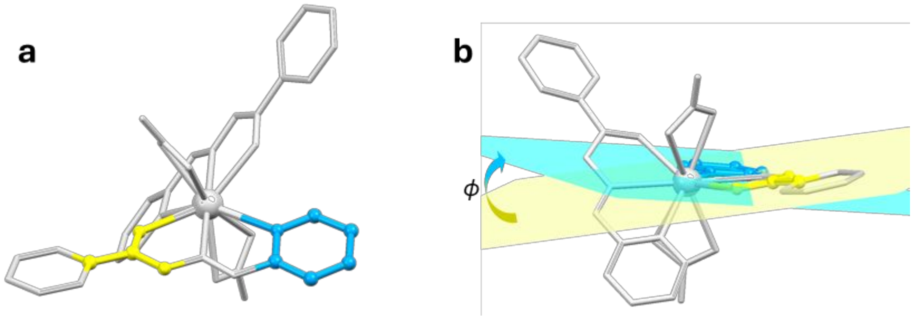

Figure S22. a) General structure of **RE–*E*** complexes (*i.e.,* **Pr–*E***) highlighting the pyridyl ring (in light blue) and the carbonyl group (in yellow). b) Figure highlighting the mean planes of the pyridyl ring (in light blue) and the carbonyl group (in yellow), and the *φ* angle between them for one of the ligands. Solvent molecules, unbound nitrate ions and hydrogen atoms were omitted for clarity.

Crystal Structure of $[La^{III}((E)\text{–}L^{py})_2(NO_3)_3]\cdot(2MeOH)$

The bond lengths for La–N(imine) ranged from 2.73 Å (La–N21) to 2.77 Å (La–N22) and the La–N(pyridyl) bonds ranged from 2.82 Å (La–N31) to 2.85 Å (La–N32). The La–O(carbonyl) bond lengths were slightly shorter, at 2.57 Å (La–O11) and 2.60 Å (La–O12), reflecting the stronger interaction between La^{III} and the carbonyl group. One of the two nitrates coordinated to the same side of the metal complex (defined by the equatorial coordination of the ligands), was bound in a symmetrical fashion, with the La–O(nitrate) distances of 2.66 Å and 2.67 Å. As usually happens for the nitrate anions, the two other nitrates were bound asymmetrically to the metal, with one La–O bond longer than the other, with distances of 2.63 Å and 2.74 Å, and 2.63 Å and 2.72 Å, respectively.

The ligands in the complexes resulted almost planar, with the angle (*φ*) between the pyridyl and carbonyl groups in the range of 5.4° and 8.0°.

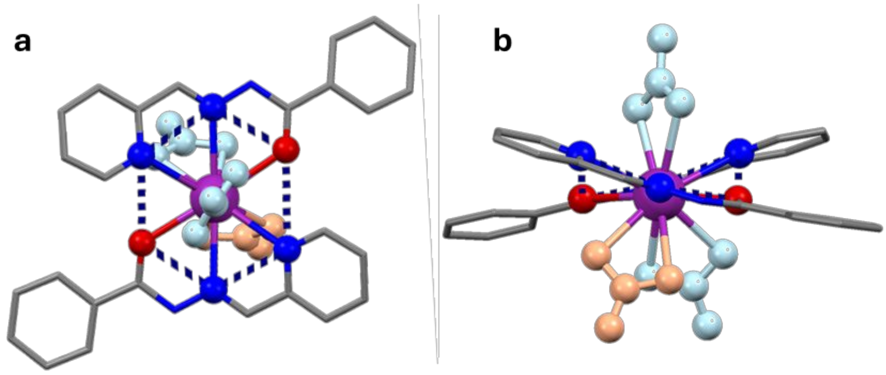

Figure 23. a) Top view of the structure of **La–*E*** highlighting the equatorial coordination of the ligands. b) Side view of the structure of **La–*E*** highlighting the apical coordination of the nitrate ions. Solvent molecules and hydrogen atoms were omitted for clarity. Symmetrically coordinated nitrates were shown in light orange, whereas the asymmetrically coordinated nitrate ion was shown in light blue.

Crystal Structure of [Pr^{III}((E)–L^{py})$_2$(NO_3)$_2$](NO_3)·0.25(H_2O)

One water molecule was present in the structure and was refined with a site occupancy of 0.25. The coordination of the two ligands was almost identical, with bond lengths for Pr–N(imine) of 2.64 Å (Pr–N21) and 2.65 Å (Pr–N22), Pr–N(pyridyl) bond lengths both of 2.69 Å, and Pr–O(carbonyl) bond lengths both of 2.48 Å. The two nitrates were coordinated the metal in a symmetrical fashion, with the Pr–O(nitrate) distances of 2.53 Å and 2.55 Å for the first nitrate, and 2.57 Å and 2.59 Å for the second one.

Crystal Structure of [Ce^{III}((E)–L^{py})$_2$(NO_3)$_3$][Ce^{III}((E)–L^{py})$_2$(NO_3)$_2$](NO_3)·(MeOH)

The two ligands in the first complex had similar bond lengths to the metal center, with the Ce1–N(imine) distances of 2.67 Å and 2.68 Å, the Ce1–N(pyridyl) distances in the range 2.77 Å and 2.80 Å and the Ce1–O(carbonyl) distances of 2.58 Å. The coordination of the ligands in the second complex entity was different from the one in the first complex entity, with the Ce2–O(carbonyl) distances being shorter (2.48 Å), conversely to the Ce2–N(imine) and Ce2–N(pyridyl) distances which were very similar for both ligands, in the range 2.65–2.67 Å and 2.72–2.77 Å, respectively.

As in the case of the [La^{III}((*E*)–L^{py})$_2$(NO_3)$_3$]·2(MeOH) structure, one of the two nitrates coordinated to the same side of the metal complex, was bound in a symmetrical fashion, with the Ce1–O(nitrate) distances of 2.69 Å and 2.71 Å. The other two nitrates were bound asymmetrically to the metal with distances of 2.61 Å and 2.69 Å, and 2.68 Å and 2.73 Å, respectively. Both nitrates in the second complex entity were bound symmetrically to the metal ion, with Ce2–O(nitrate) distances of 2.53 Å and 2.54 Å for the first nitrate, and 2.57 Å for the second one.

Table S2. Selected structural parameters for the structures of RE–L^{py} complexes. Standard deviations are reported in parentheses and refer to the last significant digits.

Compound	Atom label	coordination number	charge of the complex	^{a}metal $\%V_{free}$	$^{b}\varphi$ (°)	Ligand RE–O distance (Å)	Ligand C=O distance (Å)
**La–*E***	La	12	0	1.7	8.0	2.5672(12)	1.240(2)
					5.4	2.5929(12)	1.229(2)
**Ce–*E***	Ce1	12	0	1.7	20.2	2.576(7)	1.254(11)
					23.9	2.585(6)	1.258(10)
	Ce2	10	+1	5.6	7.0	2.486(6)	1.248(12)
					8.9	2.484(6)	1.235(11)
**Pr–*E***	Pr	10	+1	5.3	14.7	2.4784(14)	1.247(2)
					14.4	2.4783(13)	1.246(2)
**Nd–*E***	Nd	10	+1	5.2	14.4	2.465(2)	1.250(4)
					14.5	2.467(2)	1.241(4)
**Sm–*E***	Sm	10	+1	5.1	8.30	2.454(3)	1.250(4)
					17.8	2.471(2)	1.250(4)
**Eu–*E***	Eu	10	+1	5.0	8.4	2.4391(19)	1.245(3)
					17.6	2.4587(18)	1.250(3)
**Gd–*E***	Gd	10	+1	5.0	8.4	2.4258(18)	1.245(3)
					17.3	2.4510(17)	1.246(3)
**Tb–*E***	Tb	10	+1	4.9	8.3	2.4148(11)	1.2482(18)
					17.1	2.4313(11)	1.2484(17)
**Dy–*E***	Dy	10	+1	4.9	8.2	2.428(3)	1.249(6)
					16.9	2.401(3)	1.246(6)
**Y–*E***	Y	10	+1	4.8	8.2	2.3903(18)	1.247(3)
					16.9	2.4103(17)	1.242(3)
**Ho–*E***	Ho1	10	+1	4.8	9.8	2.3799(13)	1.249(2)
					16.7	2.4269(13)	1.241(2)
	Ho2	10	+1	4.8	17.3	2.4058(13)	1.241(2)
					6.3	2.4101(13)	1.247(2)
**Tm–*E***	Tm1	10	+1	4.7	10.2	2.3570(12)	1.248(2)
					16.46	2.4064(12)	1.2432(19)
	Tm2	10	+1	4.7	17.0	2.3793(11)	1.248(2)
					5.5	2.3897(12)	1.246(2)
**Yb–*E***	Yb	10	+1	4.6	6.7	2.352(5)	1.249(9)
					4.4	2.343(6)	1.241(10)
**Lu–*E***	Lu1	10	+1	4.7	16.8	2.3643(14)	1.246(2)
					5.3	2.3774(14)	1.244(2)
	Lu2	10	+1	4.7	16.3	2.3448(15)	1.249(2)
					10.3	2.3991(14)	1.246(2)

$^{a}\%V_{free} = 100–\%V_{bur}$, calculated with the SambVca program.[6] ^{b}angle between the planes generated by the C–C=O–N and pyridine ring, respectively, for the same ligand.

Table S3. Selected structural parameters for the structures of RE–*E* complexes highlighting the different coordination geometries so of the RE cations calculated with the SHAPE program.

Compound	Atom label	[a]JBCSAPR–10	[b]JSPC–10	[c]IC–12
**La–*E***	La	–	–	1.850
**Ce–*E***	Ce1	–	–	1.680
	Ce2	3.741	4.253	–
**Pr–*E***	Pr	3.443	3.400	–
**Nd–*E***	Nd	3.395	3.295	–
**Sm–*E***	Sm	3.238	3.739	–
**Eu–*E***	Eu	3.217	3.690	–
**Gd–*E***	Gd	3.194	3.656	–
**Tb–*E***	Tb	3.158	3.572	–
**Dy–*E***	Dy	3.113	3.520	–
**Y–*E***	Y	3.083	3.553	–
**Er–*E***	Er	3.105	3.191	–
**Tm–*E***	Tm1	3.144	2.963	–
	Tm2	3.132	2.795	–
**Yb–*E***	Yb	3.054	3.820	–
**Ho–*E***	Ho1	3.170	3.125	–
	Ho2	3.148	3.038	–
**Lu–*E***	Lu1	3.109	2.882	–
	Lu2	3.098	2.711	–

[a]Bicapped square antiprism, c.n. 10. [b]Sphenocorona, c.n. 10. [c]Icosahedron, c.n. 10.

Table S4. Selected geometric parameters for La–*E* and Ce–*E*. Standard deviations are reported in parentheses and refer to the last significant digit.

**La–*E***		**Ce–*E***			
Bond	Bond length (Å)	Bond	Bond length (Å)	Bond	Bond length (Å)
La–O11	2.5672(12)	Ce1–O11	2.576(7)	Ce2–O16	2.486(6)
La–O12	2.5929(12)	Ce1–O12	2.585(6)	Ce2–O17	2.484(6)
La–N21	2.7343(15)	Ce1–N21	2.676(8)	Ce2–N26	2.652(8)
La–N22	2.7724(15)	Ce1–N22	2.686(7)	Ce2–N27	2.673(8)
La–N32	2.8486(15)	Ce1–N31	2.776(8)	Ce2–N36	2.716(8)
La–N31	2.8222(15)	Ce1–N32	2.797(8)	Ce2–N37	2.770(8)
La–O13	2.6285(14)	Ce1–O13	2.613(6)	Ce2–O18	2.541(7)
La–O23	2.7442(14)	Ce1–O23	2.687(6)	Ce2–O28	2.533(7)
La–O14	2.6305(13)	Ce1–O14	2.675(6)	Ce2–O210	2.570(7)
La–O24	2.7217(14)	Ce1–O24	2.731(6)	Ce2–O310	2.566(7)
La–O15	2.6580(13)	Ce1–O15	2.690(7)		
La–O25	2.6772(13)	Ce1–O25	2.709(7)		

Table S5. Selected geometric parameters for Pr–*E*, Nd–*E* and Sm–*E*. Standard deviations are reported in parentheses and refer to the last significant digit.

**Pr–*E***		**Nd–*E***		**Sm–*E***	
Bond	Bond length (Å)	Bond	Bond length (Å)	Bond	Bond length (Å)
Pr–O11	2.4784(14)	Nd–O11	2.465(2)	Sm–O11	2.454(3)
Pr–O12	2.4783(13)	Nd–O12	2.467(2)	Sm–N21	2.604(3)
Pr–N21	2.6463(16)	Nd–N21	2.621(3)	Sm–N31	2.628(3)
Pr–N22	2.6424(16)	Nd–N22	2.631(3)	Sm–O12	2.471(2)
Pr–N31	2.6887(16)	Nd–N31	2.674(3)	Sm–N22	2.597(3)
Pr–N32	2.6888(16)	Nd–N32	2.672(3)	Sm–N32	2.657(3)
Pr–O13	2.5338(15)	Nd–O13	2.542(3)	Sm–O13	2.483(3)
Pr–O23	2.5592(15)	Nd–O23	2.515(3)	Sm–O23	2.522(3)
Pr–O14	2.5692(14)	Nd–O14	2.546(3)	Sm–O14	2.528(3)
Pr–O24	2.5839(14)	Nd–O24	2.569(3)	Sm–O24	2.489(3)

Table S6. Selected geometric parameters for Eu–*E*, Gd–*E* and Tb–*E*. Standard deviations are reported in parentheses and refer to the last significant digit.

**Eu–*E***		**Gd–*E***		**Tb–*E***	
Bond	Bond length (Å)	Bond	Bond length (Å)	Bond	Bond length (Å)
Eu–O11	2.4391(19)	Gd–O11	2.4258(18)	Tb–O11	2.4148(11)
Eu–N21	2.590(2)	Gd–N21	2.580(2)	Tb–N21	2.5697(12)
Eu–N31	2.619(2)	Gd–N31	2.609(2)	Tb–N31	2.5987(12)
Eu–O12	2.4587(18)	Gd–O12	2.4510(17)	Tb–O12	2.4313(11)
Eu–N22	2.585(2)	Gd–N22	2.575(2)	Tb–N22	2.5614(12)
Eu–N32	2.642(2)	Gd–N32	2.632(2)	Tb–N32	2.6214(13)
Eu–O13	2.4691(19)	Gd–O13	2.4602(18)	Tb–O13	2.4403(12)
Eu–O23	2.5099(19)	Gd–O23	2.4978(18)	Tb–O23	2.4859(12)
Eu–O14	2.4780(19)	Gd–O14	2.4665(18)	Tb–O14	2.4533(11)
Eu–O24	2.509(2)	Gd–O24	2.4953(19)	Tb–O24	2.4838(12)

Table S7. Selected geometric parameters for Dy–*E* and Y–*E*. Standard deviations are reported in parentheses and refer to the last significant digit.

**Dy–*E***		**Y–*E***	
Bond	Bond length (Å)	Bond	Bond length (Å)
Dy–O11	2.428(3)	Y–O11	2.3903(18)
Dy–O12	2.401(3)	Y–O12	2.4103(17)
Dy–N21	2.549(4)	Y–N21	2.557(2)
Dy–N22	2.568(4)	Y–N22	2.543(2)
Dy–N31	2.609(4)	Y–N31	2.585(2)
Dy–N32	2.588(4)	Y–N32	2.609(2)
Dy–O13	2.433(3)	Y–O13	2.4170(18)
Dy–O23	2.477(4)	Y–O23	2.4588(18)
Dy–O14	2.445(3)	Y–O14	2.4292(19)
Dy–O24	2.475(3)	Y–O24	2.4508(19)

Table S8. Selected geometric parameters Ho–*E*. Standard deviations are reported in parentheses and refer to the last significant digit.

**Ho–*E***			
Bond	Bond length (Å)	Bond	Bond length (Å)
Ho1–O11	2.3799(13)	Ho2–O13	2.4058(13)
Ho1–N21	2.5470(16)	Ho2–N23	2.5433(16)
Ho1–N31	2.5791(16)	Ho2–N33	2.5932(16)
Ho1–O12	2.4269(13)	Ho2–O14	2.4101(13)
Ho1–N22	2.5429(16)	Ho2–N24	2.5533(16)
Ho1–N32	2.6207(16)	Ho2–N34	2.5806(16)
Ho1–O15	2.4161(14)	Ho2–O18	2.4248(14)
Ho1–O25	2.4671(14)	Ho2–O28	2.4608(14)
Ho1–O16	2.4246(14)	Ho2–O19	2.4407(14)
Ho1–O26	2.4655(15)	Ho2–O29	2.4503(14)

Table S9. Selected geometric parameters for Tm–*E*. Standard deviations are reported in parentheses and refer to the last significant digit.

**Tm–*E***			
Bond	Bond length (Å)	Bond	Bond length (Å)
Tm1–O11	2.3570(12)	Tm2–O13	2.3793(11)
Tm1–N21	2.5292(14)	Tm2–N23	2.5271(14)
Tm1–N31	2.5662(14)	Tm2–N33	2.5778(14)
Tm1–O12	2.4064(12)	Tm2–O14	2.3897(12)
Tm1–N22	2.5244(14)	Tm2–N24	2.5356(14)
Tm1–N32	2.6095(14)	Tm2–N34	2.5654(14)
Tm1–O15	2.3913(12)	Tm2–O18	2.4059(12)
Tm1–O25	2.4435(12)	Tm2–O28	2.4351(12)
Tm1–O16	2.4047(12)	Tm2–O19	2.4265(12)
Tm1–O26	2.4464(13)	Tm2–O29	2.4272(13)

Table S10. Selected geometric parameters for Yb–*E* and Lu–*E*. Standard deviations are reported in parentheses and refer to the last significant digit.

**Yb–*E***		**Lu–*E***			
Bond	Bond length (Å)	Bond	Bond length (Å)	Bond	Bond length (Å)
Yb–O11	2.354(5)	Lu1–O11	2.3643(14)	Lu2–O15	2.3448(15)
Yb–O12	2.345(6)	Lu1–O12	2.3774(14)	Lu2–O16	2.3991(14)
Yb–N21	2.516(7)	Lu1–N21	2.5190(17)	Lu2–N25	2.5198(17)
Yb–N22	2.551(7)	Lu1–N22	2.5262(17)	Lu2–N26	2.5139(18)
Yb–N31	2.529(7)	Lu1–N31	2.5667(17)	Lu2–N35	2.5563(18)
Yb–N32	2.584(7)	Lu1–N32	2.5513(17)	Lu2–N36	2.5957(17)
Yb–O13	2.401(6)	Lu1–O13	2.3907(14)	Lu2–O17	2.3771(15)
Yb–O23	2.418(6)	Lu1–O23	2.4182(15)	Lu2–O27	2.4288(15)
Yb–O14	2.417(6)	Lu1–O14	2.4078(15)	Lu2–O18	2.3915(15)
Yb–O24	2.456(6)	Lu1–O24	2.4150(15)	Lu2–O28	2.4264(15)

Table S11. Selected geometric parameters for Ce–*E*·(acetone) and Er–*E*. Standard deviations are reported in parentheses and refer to the last significant digit.

**Ce–*E*·(acetone)**				**Er–*E***	
Bond	Bond length (Å)	Bond	Bond length (Å)	Bond	Bond length (Å)
Ce1–O1A	2.585(3)	Ce1–O1B	2.530(3)	Er–O8B	2.361(3)
Ce1–O2A	2.528(3)	Ce1–O2B	2.583(4)	Er–O8A	2.366(3)
Ce1–N2A	2.725(4)	Ce1–N2B	2.731(4)	Er–N10B	2.535(3)
Ce1–N5A*	2.744(4)	Ce1–N5B	2.750(4)	Er–N10A	2.574(3)
Ce1–N3A	2.800(4)	Ce1–N3B	2.838(4)	Er–N13B	2.556(3)
Ce1–N6A	2.848(4)	Ce1–N6B	2.790(4)	Er–N13A	2.603(3)
Ce1–O9A	2.607(4)	Ce1–O9B	2.650(4)	Er–O2A	2.447(3)
Ce1–O10A	2.657(4)	Ce1–O10B	2.601(4)	Er–O3A	2.420(3)
Ce1–O3A	2.634(4)	Ce1–O3B	2.661(4)	Er–O2B	2.450(3)
Ce1–O4A	2.759(4)	Ce1–O4B	2.717(4)	Er–O3B	2.465(3)
Ce1–O6A	2.674(4)	Ce1–O6B	2.740(4)	C7A–O8A	1.238(5)
Ce1–O7A	2.724(4)	Ce1–O7B	2.673(4)	C7B–O8B	1.242(4)

*reported as Ce1–C5A in the structure due to an element misassignment.

Table S12. Summary of X–ray crystallographic data for La–*E* and Ce–E.

Compound	**La–*E***	**Ce–*E***
Structure	$[La^{III}((E)–L^{py})_2(NO_3)_3]·2(MeOH)$	$[Ce^{III}((E)–L^{py})_2(NO_3)_3][Ce^{III}((E)–L^{py})_2(NO_3)_2](NO_3)·(MeOH)$
Empirical formula	$LaC_{28}H_{30}N_9O_{13}$	$CeC_{26.5}H_{24}N_9O_{11.5}$
Formula weight	839.52	1585.33
Temperature/K	200.00	200.00
Crystal system	triclinic	triclinic
Space group	$P\overline{1}$	$P\overline{1}$
a/Å	9.4893(2)	13.872(2)
b/Å	11.6036(3)	14.420(2)
c/Å	16.5535(4)	17.415(3)
α/°	71.1560(10)	112.388(5)
β/°	82.5520(10)	98.204(5)
γ/°	89.7590(10)	96.032(5)
Volume/Å^3	1709.08(7)	3140.3(9)
Z	2	2
ρ_{calc}g/cm^3	1.631	1.677
μ/mm^{-1}	1.327	1.525
F(000)	844.0	1584.0
Crystal size/mm^3	0.07 × 0.06 × 0.03	0.23 × 0.22 × 0.2
Radiation	MoKα (λ = 0.71073)	MoKα (λ = 0.71073)
2Θ range for data collection/°	4.332 to 53.22	3.93 to 51.364
Index ranges	$-11 \leq h \leq 11$, $-14 \leq k \leq 14$, $-20 \leq l \leq 20$	$-16 \leq h \leq 16$, $-17 \leq k \leq 17$, $-21 \leq l \leq 21$
Reflections collected	75500	56234
Independent reflections	7083 [R_{int} = 0.0540, R_{sigma} = 0.0233]	11772 [R_{int} = 0.1653, R_{sigma} = 0.1585]
Data/restraints/parameters	7083/98/502	11772/154/900
Goodness–of–fit on F^2	1.072	1.003
Final R indexes [I>=2σ (I)]	R_1 = 0.0198, wR_2 = 0.0466	R_1 = 0.0699, wR_2 = 0.1513
Final R indexes [all data]	R_1 = 0.0225, wR_2 = 0.0480	R_1 = 0.1491, wR_2 = 0.1875
Largest diff. peak/hole / e Å^{-3}	0.57/-0.46	1.78/-0.90

Table S13. Summary of X–ray crystallographic data for Pr–*E* and Nd–*E*.

Compound	**Pr–*E***	**Nd–*E***
Structure	$[Pr^{III}((E)–L^{py})_2(NO_3)_2](NO_3)·0.25(H_2O)$	$[Nd^{III}((E)–L^{py})_2(NO_3)_2](NO_3)$
Empirical formula	$PrC_{26}H_{22.6}N_9O_{11.3}$	$NdC_{26}H_{22}N_9O_{11}$
Formula weight	782.84	780.76
Temperature/K	200.00	200.00
Crystal system	triclinic	triclinic
Space group	$P\bar{1}$	$P\bar{1}$
a/Å	10.1671(6)	10.1526(3)
b/Å	11.2187(6)	11.2243(4)
c/Å	13.8772(9)	13.8363(4)
α/°	74.062(2)	73.940(2)
β/°	86.810(2)	86.771(2)
γ/°	89.7420(10)	89.851(2)
Volume/Å^3	1519.56(16)	1512.65(8)
Z	2	2
ρ_{calc}g/cm^3	1.711	1.714
μ/mm^{-1}	1.679	13.758
F(000)	782.0	778.0
Crystal size/mm^3	0.08 × 0.06 × 0.04	0.08 × 0.07 × 0.05
Radiation	MoKα (λ = 0.71073)	CuKα (λ = 1.54178)
2Θ range for data collection/°	3.776 to 56.564	6.658 to 140.134
Index ranges	-13 ≤ h ≤ 13, -14 ≤ k ≤ 14 -18 ≤ l ≤ 18	-12 ≤ h ≤ 12, -13 ≤ k ≤ 13, -16 ≤ l ≤ 16
Reflections collected	15082	11324
Independent reflections	7541 [R_{int} = 0.0200, R_{sigma} = 0.0263]	5715 [R_{int} = 0.0379, R_{sigma} = 0.0513]
Data/restraints/parameters	7541/639/465	5715/50/432
Goodness–of–fit on F^2	1.095	1.049
Final R indexes [I>=2σ (I)]	R_1 = 0.0220, wR_2 = 0.0502	R_1 = 0.0342, wR_2 = 0.0761
Final R indexes [all data]	R_1 = 0.0243, wR_2 = 0.0513	R_1 = 0.0398, wR_2 = 0.0781
Largest diff. peak/hole / e Å^{-3}	0.70/-0.95	1.37/-0.47

Table S14. Summary of X–ray crystallographic data for Sm–*E* and Eu–*E*.

Compound	**Sm–*E***	**Eu–*E***
Structure	$[Sm^{III}((E)–L^{py})_2(NO_3)_2](NO_3)$	$[Eu^{III}((E)–L^{py})_2(NO_3)_2](NO_3)$
Empirical formula	$SmC_{26}H_{22}N_9O_{11}$	$EuC_{26}H_{22}N_9O_{11}$
Formula weight	786.87	788.48
Temperature/K	200.00	200.00
Crystal system	Triclinic	triclinic
Space group	$P\bar{1}$	$P\bar{1}$
a/Å	8.345(3)	8.3283(19)
b/Å	12.316(5)	12.287(3)
c/Å	15.337(4)	15.307(2)
α/°	75.885(8)	75.947(4)
β/°	86.338(13)	86.457(5)
γ/°	81.587(7)	81.468(5)
Volume/Å^3	1511.6(9)	1502.1(5)
Z	2	2
ρ_{calc}g/cm^3	1.729	1.743
μ/mm^{-1}	2.017	2.163
F(000)	782.0	784.0
Crystal size/mm^3	0.08 × 0.04 × 0.03	0.07 × 0.05 × 0.03
Radiation	MoKα (λ = 0.71073)	MoKα (λ = 0.71073)
2Θ range for data collection/°	3.856 to 52.044	3.866 to 53.464
Index ranges	$-10 \le h \le 10$, $-15 \le k \le 15$, $-18 \le l \le 17$	$-10 \le h \le 10$, $-15 \le k \le 15$, $-19 \le l \le 19$
Reflections collected	10898	11457
Independent reflections	5938 [R_{int} = 0.0351, R_{sigma} = 0.0564]	6379 [R_{int} = 0.0247, R_{sigma} = 0.0400]
Data/restraints/parameters	5938/137/460	6379/127/451
Goodness–of–fit on F^2	1.043	1.055
Final R indexes [I>=2σ (I)]	R_1 = 0.0403, wR_2 = 0.0875	R_1 = 0.0279, wR_2 = 0.0651
Final R indexes [all data]	R_1 = 0.0455, wR_2 = 0.0906	R_1 = 0.0305, wR_2 = 0.0663
Largest diff. peak/hole / e Å^{-3}	1.18/-0.57	0.91/-0.38

Table S15. Summary of X–ray crystallographic data for Gd–*E* and Tb–*E*.

Compound	**Gd–*E***	**Tb–*E***
Structure	$[Gd^{III}((E)–L^{py})_2(NO_3)_2](NO_3)$	$[Tb^{III}((E)–L^{py})_2(NO_3)_2](NO_3)$
Empirical formula	$GdC_{26}H_{22}N_9O_{11}$	$TbC_{26}H_{22}N_9O_{11}$
Formula weight	793.77	795.44
Temperature/K	200.00	200.00
Crystal system	triclinic	triclinic
Space group	$P\overline{1}$	$P\overline{1}$
a/Å	8.319(2)	8.3123(8)
b/Å	12.272(4)	12.2554(13)
c/Å	15.306(5)	15.2944(15)
α/°	76.031(9)	76.121(3)
β/°	86.527(8)	86.601(3)
γ/°	81.402(13)	81.235(3)
Volume/Å^3	1498.8(8)	1494.5(3)
Z	2	2
ρ_{calc}g/cm^3	1.759	1.768
μ/mm^{-1}	2.288	2.442
F(000)	786.0	788.0
Crystal size/mm^3	0.18 × 0.08 × 0.06	0.12 × 0.07 × 0.07
Radiation	MoKα (λ = 0.71073)	MoKα (λ = 0.71073)
2Θ range for data collection/°	3.872 to 53.464	3.88 to 65.054
Index ranges	-10 ≤ h ≤ 10, -15 ≤ k ≤ 15, -19 ≤ l ≤ 19	-12 ≤ h ≤ 12, -18 ≤ k ≤ 18, -22 ≤ l ≤ 22
Reflections collected	12744	62380
Independent reflections	6385 [R_{int} = 0.0285, R_{sigma} = 0.0350]	10399 [R_{int} = 0.0482, R_{sigma} = 0.0336]
Data/restraints/parameters	6385/166/472	10399/93/451
Goodness–of–fit on F^2	1.026	1.036
Final R indexes [I>=2σ (I)]	R_1 = 0.0255, wR_2 = 0.0620	R_1 = 0.0208, wR_2 = 0.0485
Final R indexes [all data]	R_1 = 0.0275, wR_2 = 0.0627	R_1 = 0.0231, wR_2 = 0.0497
Largest diff. peak/hole / e Å^{-3}	0.84/-0.72	1.08/-1.01

Table S16. Summary of X–ray crystallographic data for Dy–*E* and Y–*E*.

Compound	**Dy–*E***	**Y–*E***
Structure	[Dy^{III}((*E*)–L^{py})$_2$(NO$_3$)$_2$](NO$_3$)	[Y^{III}((*E*)–L^{py})$_2$(NO$_3$)$_2$](NO$_3$)
Empirical formula	$DyC_{26}H_{22}N_9O_{11}$	$YC_{26}H_{22}N_9O_{11}$
Formula weight	799.02	725.43
Temperature/K	200.00	200.00
Crystal system	triclinic	triclinic
Space group	P$\bar{1}$	P$\bar{1}$
a/Å	8.3234(5)	8.2945(5)
b/Å	12.2706(7)	12.2783(7)
c/Å	15.2848(9)	15.2634(9)
α/°	76.137(2)	76.278(2)
β/°	86.742(2)	86.702(2)
γ/°	81.144(2)	80.787(2)
Volume/Å^3	1497.22(15)	1490.30(15)
Z	2	2
ρ_{calc}g/cm^3	1.772	1.617
μ/mm^{-1}	2.571	2.033
F(000)	790.0	736.0
Crystal size/mm^3	0.7 × 0.7 × 0.6	0.5 × 0.5 × 0.4
Radiation	MoKα (λ = 0.71073)	MoKα (λ = 0.71073)
2Θ range for data collection/°	3.878 to 51.362	3.884 to 51.364
Index ranges	-10 ≤ h ≤ 10, -14 ≤ k ≤ 14, -18 ≤ l ≤ 18	-9 ≤ h ≤ 10, -14 ≤ k ≤ 14, -18 ≤ l ≤ 18
Reflections collected	27198	27402
Independent reflections	5672 [R_{int} = 0.0745, R_{sigma} = 0.0504]	5647 [R_{int} = 0.0629, R_{sigma} = 0.0467]
Data/restraints/parameters	5672/48/432	5647/696/583
Goodness–of–fit on F^2	1.063	1.027
Final R indexes [I>=2σ (I)]	R_1 = 0.0381, wR_2 = 0.0908	R_1 = 0.0358, wR_2 = 0.0729
Final R indexes [all data]	R_1 = 0.0441, wR_2 = 0.0939	R_1 = 0.0470, wR_2 = 0.0777
Largest diff. peak/hole / e Å^{-3}	2.17/-0.89	0.42/-0.23

Table S17. Summary of X–ray crystallographic data for Ho–*E* and Tm–*E*.

Compound	**Ho–*E***	**Tm–*E***
Structure	$[Ho^{III}((E)–L^{py})_2(NO_3)_2](NO_3)$	$[Tm^{III}((E)–L^{py})_2(NO_3)_2](NO_3)$
Empirical formula	$HoC_{26}H_{22}N_9O_{11}$	$C_{26}H_{22}N_9O_{11}Tm$
Formula weight	801.45	805.45
Temperature/K	200.00	200.00
Crystal system	triclinic	triclinic
Space group	$P\overline{1}$	$P\overline{1}$
a/Å	12.2803(5)	12.2746(9)
b/Å	15.2506(6)	15.2156(6)
c/Å	16.5746(7)	16.5223(12)
α/°	86.6940(10)	86.757(2)
β/°	80.7030(10)	80.365(2)
γ/°	76.2610(10)	76.424(2)
Volume/$Å^3$	2975.1(2)	2956.8(3)
Z	4	4
$\rho_{calc}g/cm^3$	1.789	1.809
μ/mm^{-1}	2.736	3.077
F(000)	1584.0	1592.0
Crystal size/mm^3	0.1 × 0.05 × 0.04	0.22 × 0.16 × 0.12
Radiation	MoKα (λ = 0.71073)	MoKα (λ = 0.71073)
2Θ range for data collection/°	3.672 to 54.968	3.686 to 62.036
Index ranges	$-15 \leq h \leq 15, -19 \leq k \leq 19, -21 \leq l \leq 21$	$-17 \leq h \leq 17, -21 \leq k \leq 22, -23 \leq l \leq 23$
Reflections collected	27033	242196
Independent reflections	13618 [R_{int} = 0.0397, R_{sigma} = 0.0491]	18622 [R_{int} = 0.0567, R_{sigma} = 0.0293]
Data/restraints/parameters	13618/411/912	18622/146/892
Goodness–of–fit on F^2	1.051	1.027
Final R indexes [I>=2σ (I)]	R_1 = 0.0239, wR_2 = 0.0497	R_1 = 0.0231, wR_2 = 0.0491
Final R indexes [all data]	R_1 = 0.0364, wR_2 = 0.0524	R_1 = 0.0319, wR_2 = 0.0536
Largest diff. peak/hole / e $Å^{-3}$	0.79/-0.61	0.58/-1.42

Table S18. Summary of X–ray crystallographic data for Yb–*E* and Lu–*E*.

Compound	**Yb–*E***	**Lu–*E***
Structure	$[Yb^{III}((E)–L^{py})_2(NO_3)_2](NO_3)\cdot1.5(H_2O)$	$[Lu^{III}((E)–L^{py})_2(NO_3)_2](NO_3)$
Empirical formula	$YbC_{26}H_{25}N_9O_{12.5}$	$LuC_{26}H_{22}N_9O_{11}$
Formula weight	836.59	811.49
Temperature/K	200.00	200.00
Crystal system	monoclinic	triclinic
Space group	C2/c	$P\bar{1}$
a/Å	26.962(2)	12.2717(3)
b/Å	18.2125(14)	15.2167(4)
c/Å	15.0989(13)	16.5112(3)
α/°	90	86.8530(10)
β/°	122.026(2)	80.1840(10)
γ/°	90	76.5480(10)
Volume/Å^3	6285.9(9)	2954.36(12)
Z	8	4
ρ_{calc}g/cm^3	1.768	1.824
μ/mm^{-1}	3.054	3.418
F(000)	3312.0	1600.0
Crystal size/mm^3	0.3 × 0.3 × 0.25	0.12 × 0.04 × 0.04
Radiation	MoKα (λ = 0.71073)	MoKα (λ = 0.71073)
2Θ range for data collection/°	4.472 to 51.364	3.75 to 56.74
Index ranges	-32 ≤ h ≤ 32, -22 ≤ k ≤ 22, -18 ≤ l ≤ 18	-16 ≤ h ≤ 16, -20 ≤ k ≤ 20, -19 ≤ l ≤ 22
Reflections collected	55519	95686
Independent reflections	5958 [R_{int} = 0.0978, R_{sigma} = 0.0606]	14767 [R_{int} = 0.0541, R_{sigma} = 0.0331]
Data/restraints/parameters	5958/856/493	14767/56/891
Goodness–of–fit on F^2	1.082	1.037
Final R indexes [I>=2σ (I)]	R_1 = 0.0520, wR_2 = 0.1256	R_1 = 0.0223, wR_2 = 0.0506
Final R indexes [all data]	R_1 = 0.0787, wR_2 = 0.1431	R_1 = 0.0313, wR_2 = 0.0544
Largest diff. peak/hole / e Å^{-3}	1.95/-1.34	0.54/-1.30

Table S19. Summary of X–ray crystallographic data for Ce–*E*·(acetone) and Er–*E*.

Compound	[a]**Ce–*E*·(acetone)**	[b]**Er–*E***
Structure	$[Ce^{III}((E)–L^{py})_2(NO_3)_3]\cdot 2(acetone)\cdot 2(H_2O)$	$[Er^{III}((E)–L^{py})_2(NO_3)_2](NO_3)\cdot 1.5(H_2O)$
Empirical formula	$CeC_{29}H_{32}N_9O_{14}$	$ErC_{26}H_{25}N_9O_{12.5}$
Formula weight	870.76	830.81
Temperature/K	293(2)	293(2)
Crystal system	monoclinic	monoclinic
Space group	P21/n	C2/c
a/Å	18.6342(19)	26.902(1)
b/Å	11.1941(8)	18.390(1)
c/Å	34.734(2)	15.259(1)
α/°	90.00	90.00
β/°	99.188(9)	123.06(1)
γ/°	90.00	90.00
Volume/Å^3	7152.3(10)	6326.9(6)
Z	8	8
ρ_{calc}g/cm^3	1.617	1.744
μ/mm^{-1}	1.352	2.731
F(000)	3512.0	3296.0
Crystal size/mm^3	0.6 × 0.36 × 0.27	0.25 × 0.25 × 0.15
Radiation	MoKα (λ = 0.71070)	MoKα (λ = 0.71073)
2Θ range for data collection/°	3.82 to 50.02	8.32 to 56.56
Index ranges	$-22 \leq h \leq 21$, $-5 \leq k \leq 13$, $-16 \leq l \leq 41$	$-35 \leq h \leq 35$, $-24 \leq k \leq 24$, $-17 \leq l \leq 20$
Reflections collected	13582	18947
Independent reflections	12484 [R_{int} = 0.0183, R_{sigma} = 0.0683]	7722 [R_{int} = 0.0283, R_{sigma} = 0.0523]
Data/restraints/parameters	12484/0/959	7722/14/433
Goodness–of–fit on F^2	0.945	0.928
Final R indexes [I>=2σ (I)]	R_1 = 0.0462, wR2 = 0.1005	R_1 = 0.0366, wR_2 = 0.0885
Final R indexes [all data]	R_1 = 0.1077, wR2 = 0.1162	R_1 = 0.0665, wR_2 = 0.0943
Largest diff. peak/hole / e Å^{-3}	1.13/-0.94	1.99/-0.81

[a]Structure already deposited in the CSD, ref code 131265.[15] [b]Structure already deposited in the CSD, ref code 141461.[14]

Ligand Isomerization

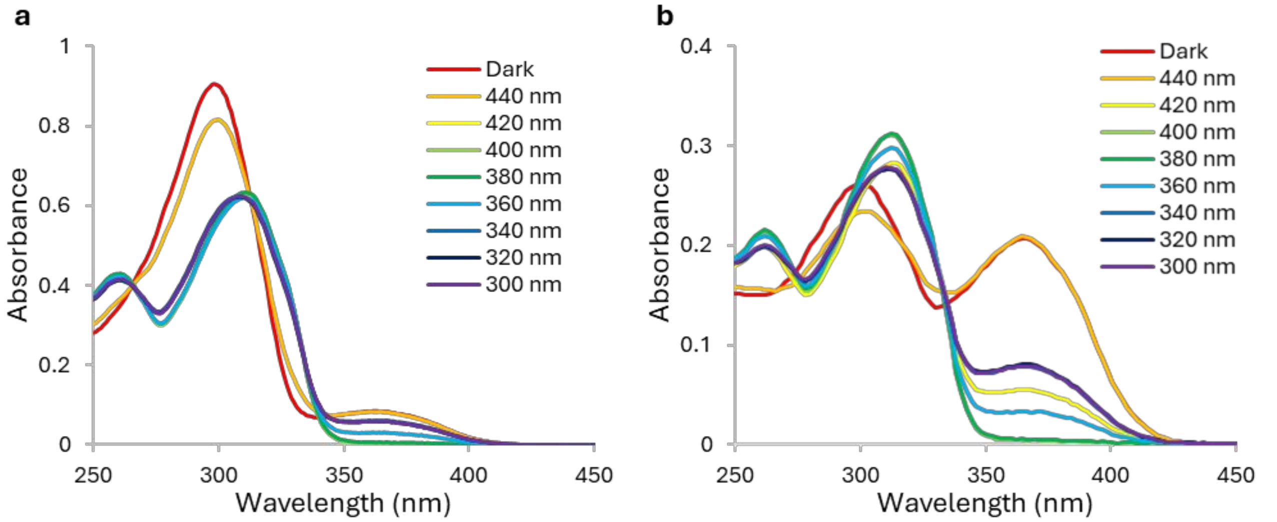

Figure S24. Wavelength scans performed at room temperature for a) **(*E*)–L**^{py} (30 µM in MeOH) and b) **Nd–*E*** (10 µM in MeOH) solutions changing the monochromatic radiation used for illumination from 440 to 300 nm with 20 nm steps for 10 minutes for each wavelength. The spectra of the dark were recorded under illumination at 600 nm, at which no isomerization is observed.

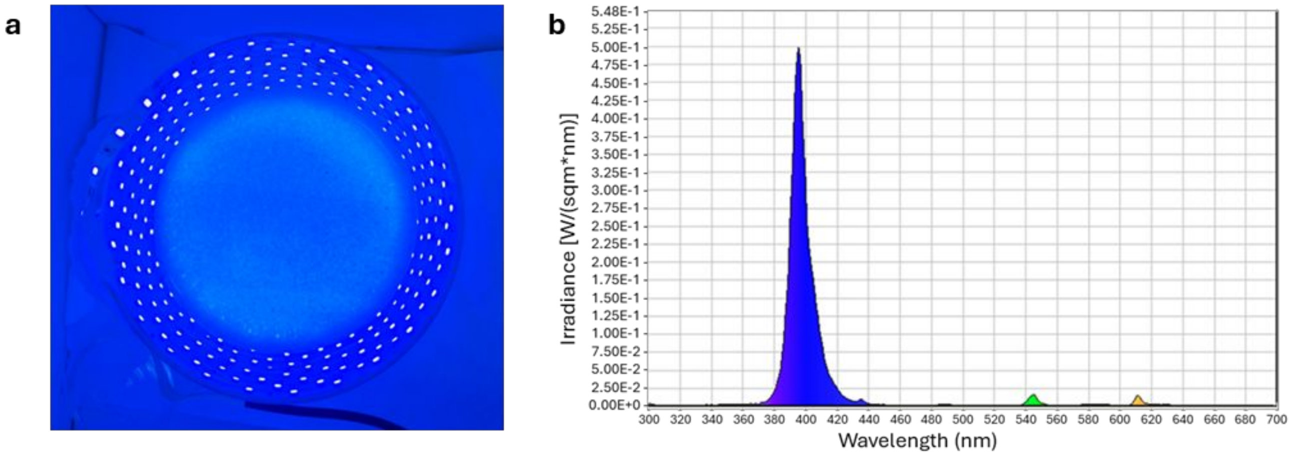

Figure S25. a) Photo of the photoreactor setup obtained by wrapping a 395 nm LED strip on the inside of a crystallizer dish. b) Emission spectra of the LED photoreactor. The measurement was acquired at 15 cm from the center of the crystallizer. The maximum emission is centered at 395 nm.

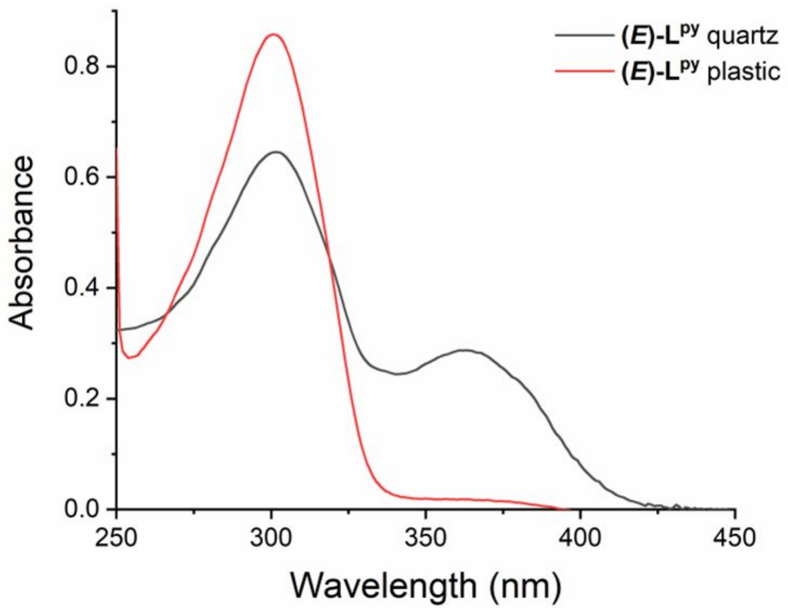

Figure S26. UV–Visible spectra of a 30 μM solution of ***(E)*–L**^{py} in MeOH using a quartz (black line) or plastic (red line) cuvette, recorded at room temperature.

The use of plastic containers for the preparation of the solutions, and plastic cuvettes for the analysis showed the disappearance of the band at 365 nm in the spectra of ***(E)*–L**^{py}, due to the lack of metal ions (mainly Na^+) in solution. Thus, the UV–Visible photochemical study was repeated using plastic containers to determine the behavior of the free ligands (for all ligands).

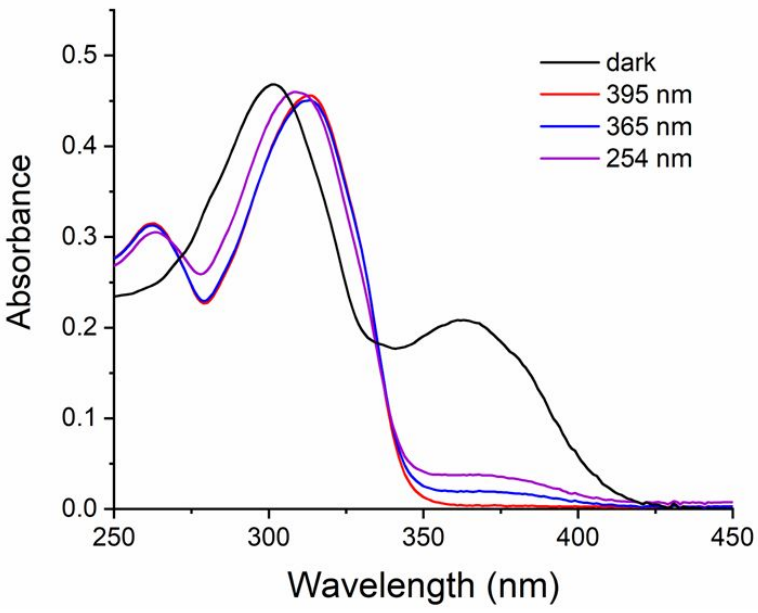

Figure S27. UV–Visible spectra of a 30 μM MeOH solution of ***(E)*–L**^{py} before (black line) and after illumination for 10 minutes with three common light sources (254 nm from the UV–B emission of a TLC lamp, purple line; 365 nm from the UV–A emission of a TLC lamp, blue line; 395 nm LED photoreactor, red line) at room temperature.

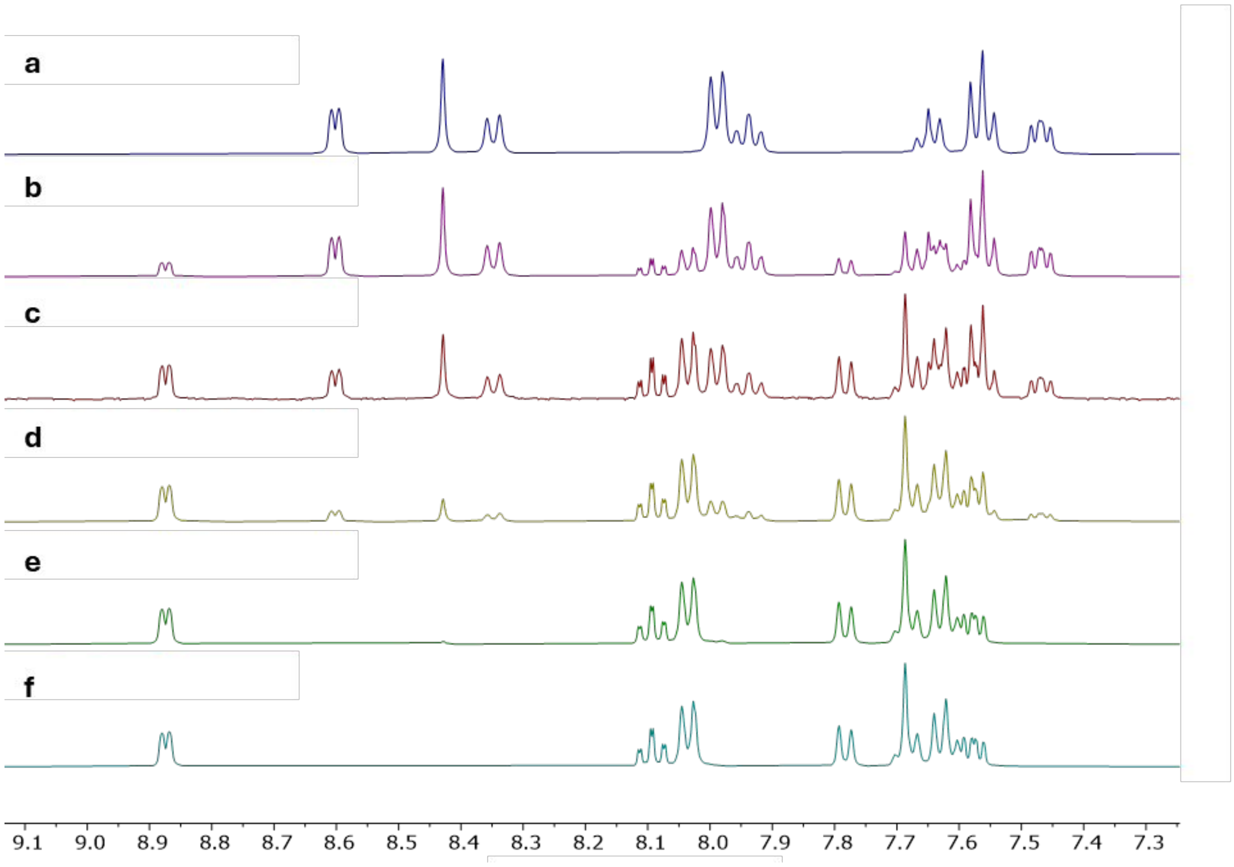

Figure S28. ^{1}H NMR (400 MHz, MeOH–d_4) of a 3.0 mM solution of **(*E*)–L^{py}** a) before illumination, after b) 10 minutes, c) 20 minutes, d) 30 minutes, e) 40 minutes and f) 50 minutes of illumination with a 395 nm LED photoreactor.

Table S20. Molar fractions (χ) of (*E*)–L^{py} and (*Z*)–L^{py} upon illumination of a 3.0 mM MeOH-d_4 solution of (*E*)–L^{py} using a 395 nm photoreactor. The molar fraction values were calculated from the integration of the H_{13} peak at 8.58 and 8.87 ppm for the *E*– and *Z*–isomers, respectively.

Illumination time (min)	χ_E	χ_Z
0	1	0
10	0.74	0.26
20	0.47	0.53
30	0.23	0.77
40	0.04	0.96
50	0.00	1

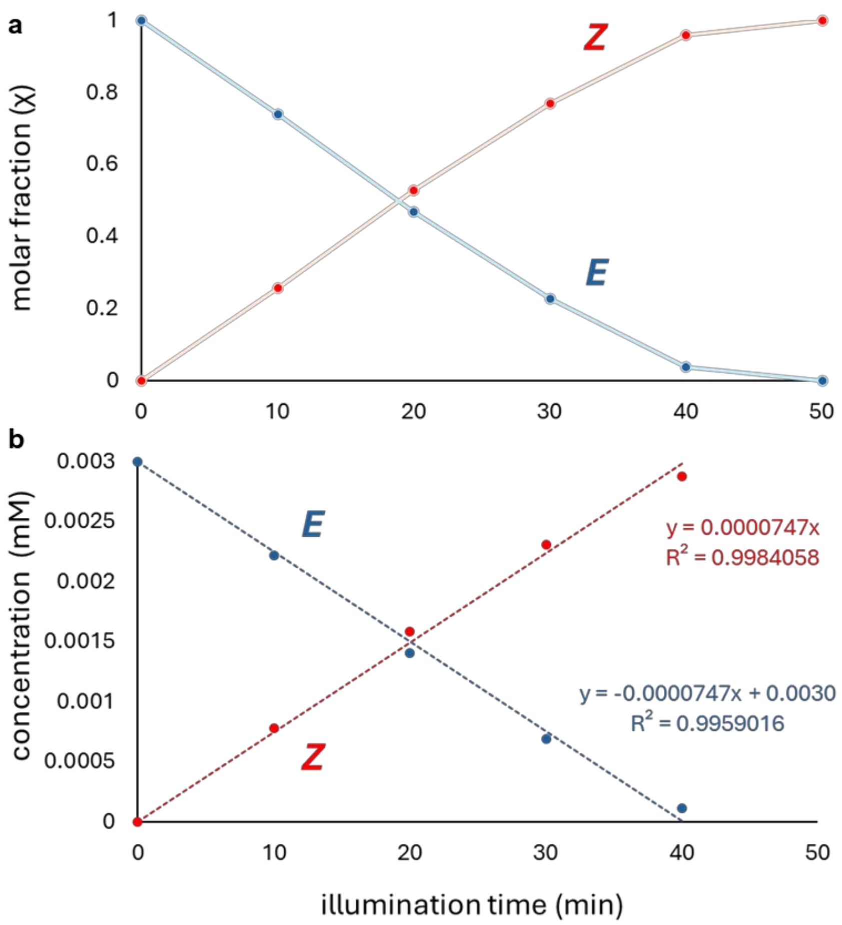

Figure S29. a) Molar fractions (χ) of ***(E)*–L**^{py} and ***(Z)*–L**^{py} in MeOH-d_4 *vs* illumination time using a 395 nm photoreactor. The molar fraction values were calculated from the integration of the H_{13} peak at 8.58 and 8.87 ppm for the *E*– and *Z*–isomers, respectively. b) Concentration of ***(E)*–L**^{py} and ***(Z)*–L**^{py} *vs* illumination time using a 395 photoreactor. Linear fits of the data points are reported along with the slopes obtained from the fitting curves.

UV–Vis Titrations

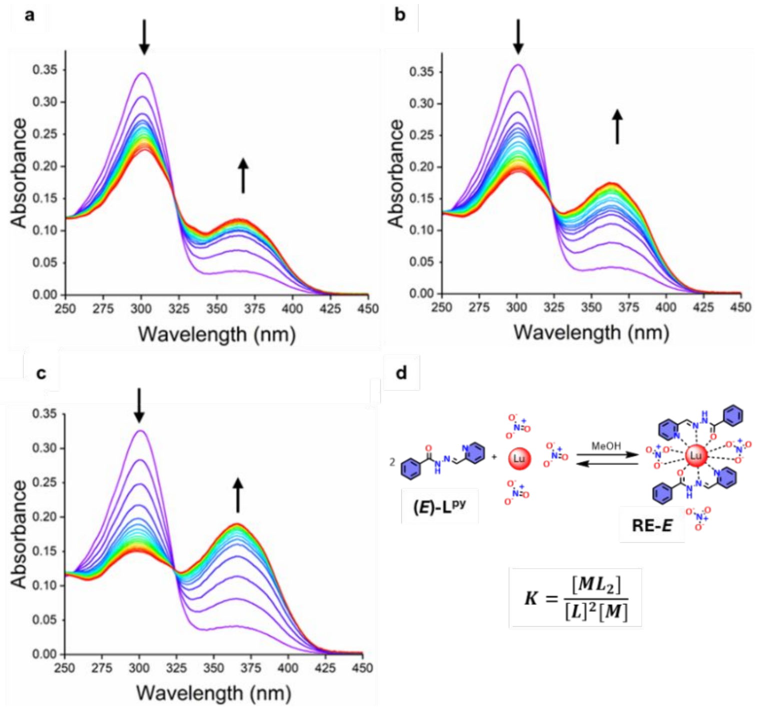

Figure S30. UV–Visible spectra depicting the titration of 15 µM ***(E)*–L**^{py} with a) $La(NO_3)_3$, b) $Y(NO_3)_3$ and c) $Lu(NO_3)_3$ in MeOH at room temperature. Spectra are color coded based on the addition of the titrant, ranging from 0 (***(E)*–L**^{py}, purple line) to 3.44 M:L ratio (red line). Arrows highlight the trend associated with incremental changes in the spectra. d) Chemical equation for the formation of **RE-*E*** complexes and associated reaction constant formula.

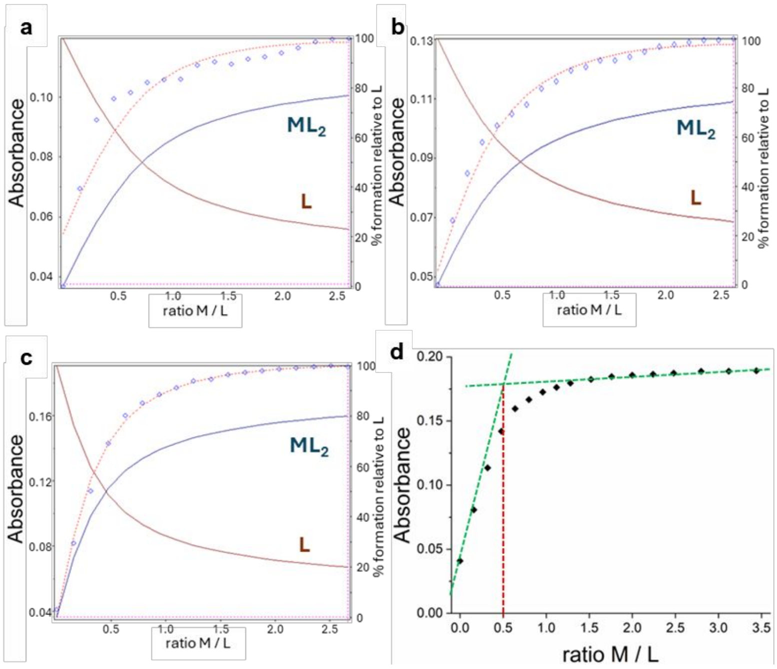

Figure S31. Fitting plots of UV–Visible titrations of 15 µM ***(E)*–L**^{py} with a) $La(NO_3)_3$, b) $Y(NO_3)_3$ and c) $Lu(NO_3)_3$ in MeOH monitoring the absorbance at 365 nm at room temperature. Brown lines indicate the percentage of free ***(E)*–L**^{py} in solution and blue lines indicate the percentage ML_2 complex. Blue diamonds indicate experimental values, and red dotted lines indicate the absorbance derived from the fitting process. d) Determination of the M:L ratio for the titration ***(E)*–L**^{py} with $Lu(NO_3)_3$ in MeOH by interpolation.

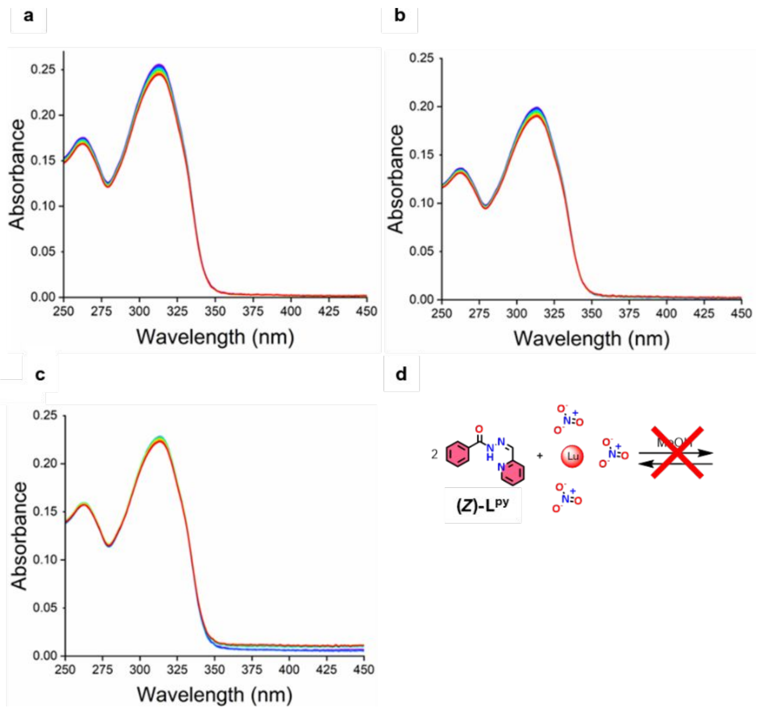

Figure S32. UV–Visible spectra depicting the titration of 15 µM ***(Z)*–L^{py}** with a) La$(NO_3)_3$, b) Y$(NO_3)_3$ and c) Lu$(NO_3)_3$ in MeOH at room temperature. Spectra are color coded based on the addition of the titrant, ranging from 0 (***(Z)*–L^{py}**, purple line) to 0.88 M:L ratio (red line). Arrows highlight the trend associated with incremental changes in the spectra. d) Chemical equation for the formation of RE-complexes with ***(Z)*–L^{py}**.

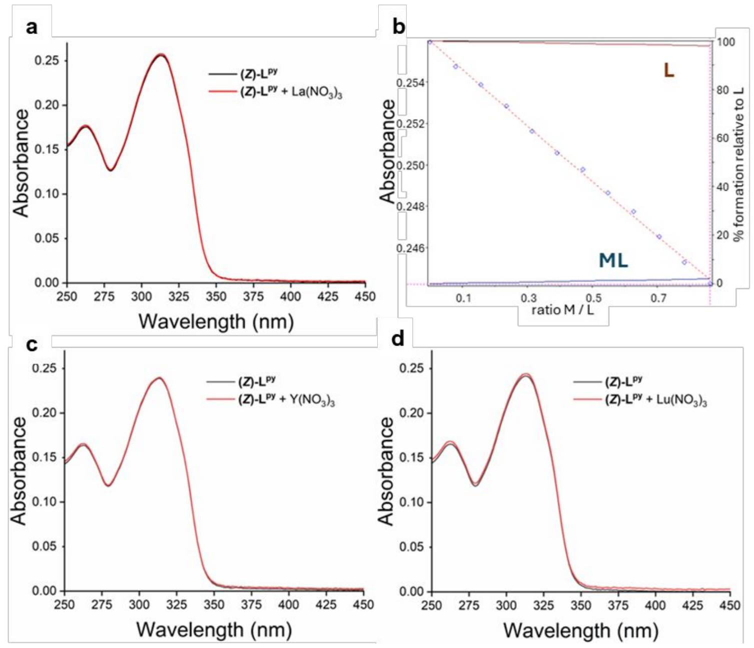

Figure S33. UV–Visible spectra of 15 µM ***(Z)*–L^{py}** (black line) and spectra of the last addition of the titration with La$(NO_3)_3$ a), Y$(NO_3)_3$ c) and Lu$(NO_3)_3$ d) in MeOH (red line), corrected for dilution (dilution factor: 1.055). b) Fitting plots of UV–Visible titrations of 15 µM ***(E)*–L^{py}** with La$(NO_3)_3$ in MeOH monitoring the absorbance at 313 nm at room temperature. Brown lines indicate the percentage of free ***(E)*–L^{py}** in solution and blue lines indicate the percentage ML complex. Blue diamonds indicate experimental values, and red dotted lines indicate the absorbance derived from the fitting process.

Table S21. Calculated log(K) values for the formation of RE-E complexes. Standard deviations are reported in parentheses and refer to the last significant digit (2σ). Free ligand percentages at equilibrium were calculated using the log(K) values for a 15 μM solution of complexes in MeOH at room temperature. Molar extinction coefficients were calculated from the absorbance spectra of 15 μM solution of complexes in methanol before illumination using the previously calculated speciation percentages, and effective quantum yield of isomerization ($\Phi^{390}_{E\rightarrow Z}$) calculated from change in absorption at 390 nm when irradiated with 390 nm LEDs.

**RE-*E***	**(*E*)-L^{py}**	**La-*E***	**Ce-*E***	**Pr-*E***	**Nd-*E***	**Sm-*E***	**Eu-*E***	**Gd-*E***
log(K)	-	10.9(5)	11.5(2)	11.6(2)	12.4(2)	12.3(5)	12.14(7)	12.0(4)
% free ligand	-	22.0	14.1	13.1	7.4	7.9	9.0	10.1
ε^{390} (L mol^{-1} cm^{-1})	1790	10647	9938	10347	10504	11707	11574	11712
ε^{365} (L mol^{-1} cm^{-1})	3202	18857	17368	17902	17888	20086	20098	20749
$\Phi^{390}_{E\rightarrow Z}$	2.33×10^{-3}	-	-	9.6×10^{-6}	-	-	-	2.81×10^{-5}
	**Tb-*E***	**Dy-*E***	**Y-*E***	**Er-*E***	**Ho-*E***	**Tm-*E***	**Yb-*E***	**Lu-*E***
log(K)	12.3(4)	12.41(3)	12.36(7)	12.4(2)	12.4(1)	12.19(7)	12.2(2)	11.4(4)
% free ligand	7.8	7.4	7.1	7.7	7.0	8.7	8.9	16.1
ε^{390} (L mol^{-1} cm^{-1})	12080	11179	12268	12263	11864	12623	13207	14131
ε^{365} (L mol^{-1} cm^{-1})	21555	20413	23662	21707	20978	21559	21782	22788
$\Phi^{390}_{E\rightarrow Z}$	-	-	-	-	-	-	1.21×10^{-5}	1.87×10^{-4}

Complexes Isomerization

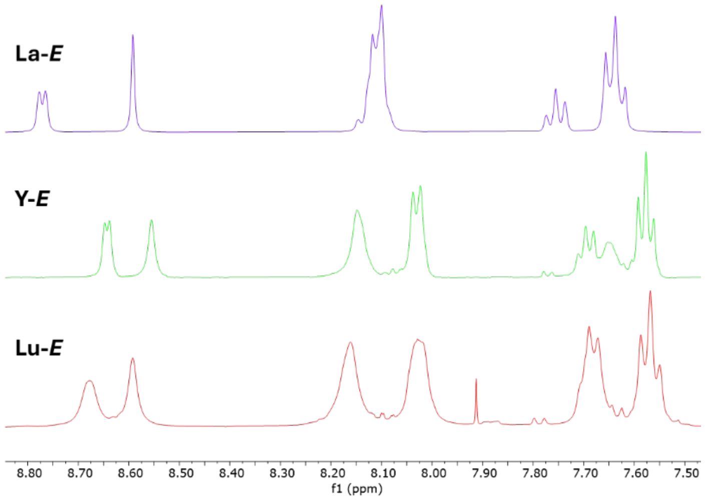

Figure S34. From top to bottom, ^{1}H–NMR spectra (400 MHz, MeOH-d_4) of **La-*E***, **Y-*E*** and **Lu-*E***. 500 MHz in the case of **Y-*E***. Impurities in the spectra were attributed to a small fraction of **(*Z*)-L^{py}** and residual solvents.

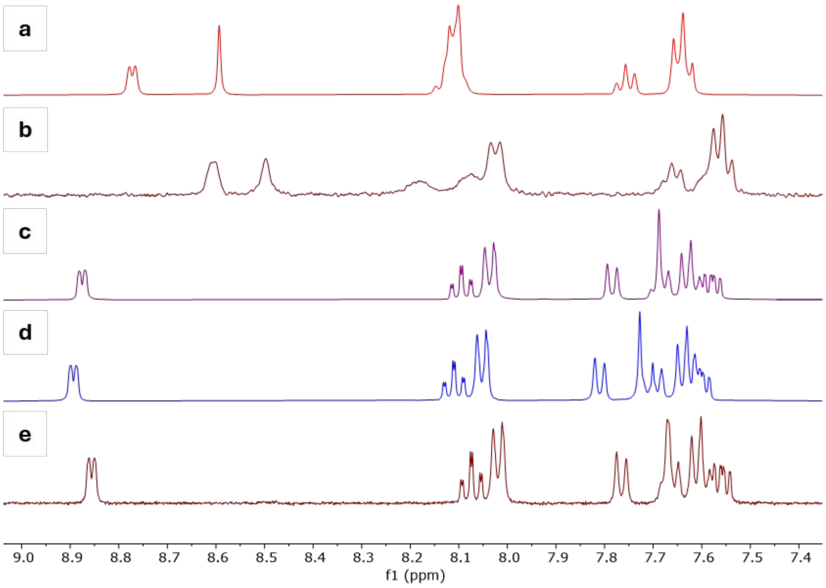

Figure S35. ^{1}H NMR (400 MHz, MeOH–d_4) of a 5.0 mM solution of a) **La–*E*** and b) **Y–*E*** before illumination. ^{1}H NMR (400 MHz, MeOH–d_4) of a 5.0 mM solution of c) **(*E*)–L^{py}**, d) **La–*E*** and e) **Y–*E*** after illumination with a 395 nm photoreactor for 60 minutes (100% conversion to **(*Z*)–L^{py}**).

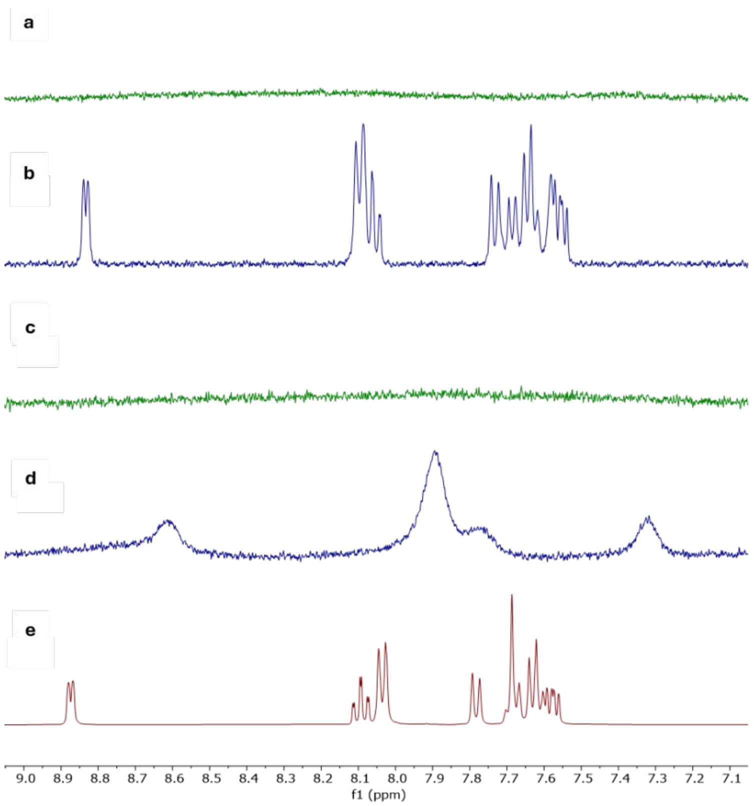

Figure S36. ^{1}H NMR (400 MHz, MeOH–d_4) of **Nd–*E*** (5.0 mM) a) before and b) after illumination with a 395 nm photoreactor for 60 minutes. ^{1}H NMR (400 MHz, MeOH–d_4) of **Dy–*E*** (5.0 mM) c) before and d) after illumination with a 395 nm photoreactor for 60 minutes. e) ^{1}H NMR (400 MHz, MeOH–d_4) of **(*E*)–L^{py}** after illumination with a 395 nm photoreactor for 60 minutes (100% conversion to **(*Z*)–L^{py}**).

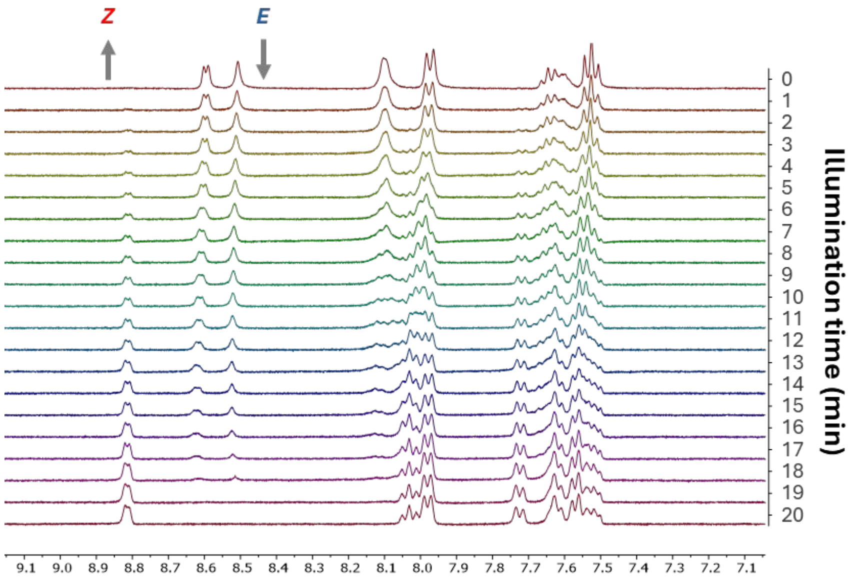

Figure S37. ^{1}H NMR (400 MHz, MeOH–d_4) of a 5.0 mM solution of **Y–*E*** after intervals of 60 seconds of illumination with a 390 nm Kessil lamp (from 0 on the top, to 20 minutes at the bottom). 100% conversion to **(*Z*)–L^{py}**.

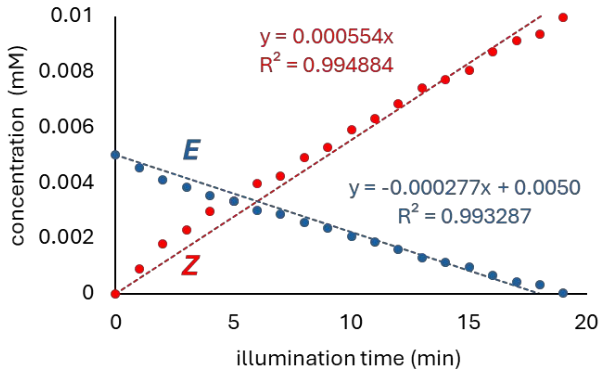

Figure S38. Concentration of **Y–*E*** and **(*Z*)–L**^{py} *vs* illumination time using a 390 nm Kessil lamp. The concentration values were calculated from the molar fractions obtained by the integration of the H_{13} peak at 8.60 and 8.86 ppm for the *E*–isomer in **Y–*E*** and *Z*–isomers, respectively. Linear fits of the data points are reported along with the slopes obtained from the fitting curves.

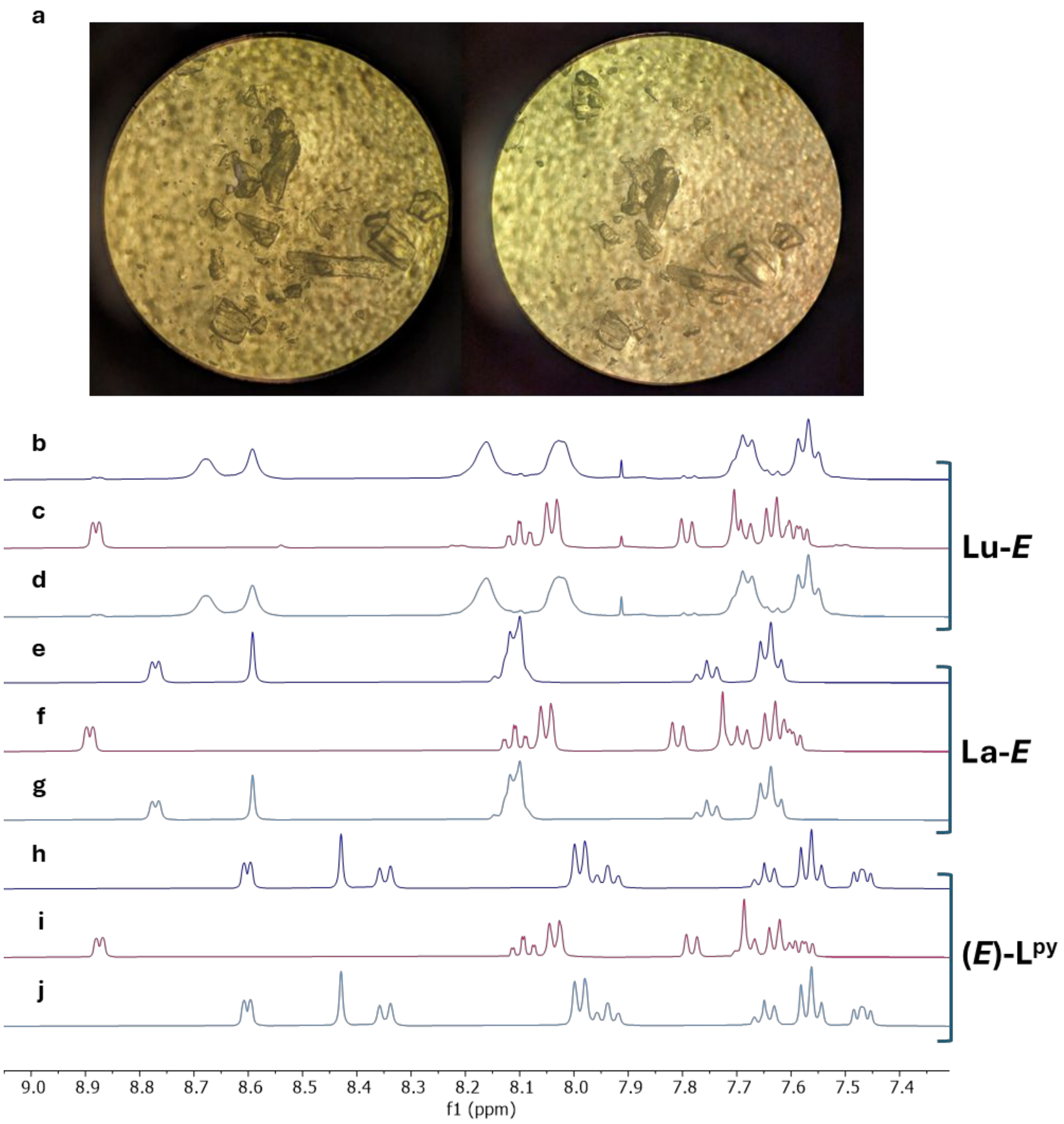

Figure S39. a) Optical microscope photo (40x magnification) of the crystals of **(*E*)–L^{py}** before (right) and after (left) illumination inside the 395 nm LED photoreactor. ^{1}H NMR (400 MHz, MeOH–d_4) spectra of **Lu–*E*** c) before illumination, d) after illumination of the solution, and d) after illumination of the solid prior to NMR sample preparation. ^{1}H NMR (400 MHz, MeOH–d_4) spectra of **La–*E*** e) before illumination, f) after illumination of the solution, and g) after illumination of the solid prior to NMR sample preparation. ^{1}H NMR (400 MHz, MeOH–d_4) spectra of **(*E*)–L^{py}** h) before illumination, i) after illumination of the solution, and j) after illumination of the solid prior to NMR sample preparation, respectively.

Bistability Study

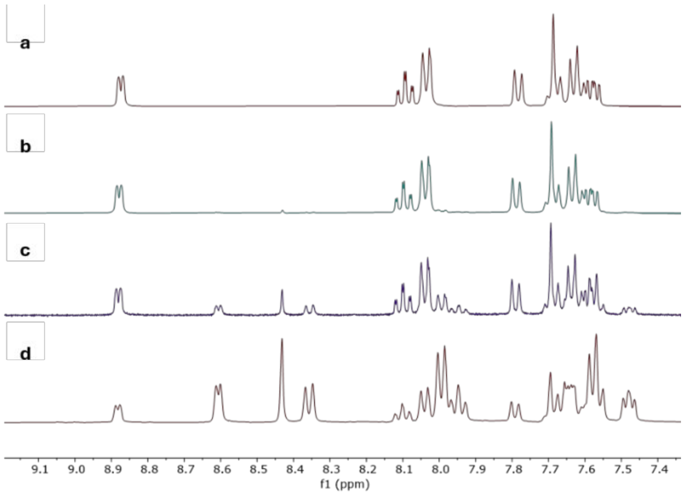

Figure S40. ^{1}H NMR (400 MHz, MeOH–d_4) of a 3.0 mM solution of **(*E*)–L^{py}** after illumination with a 395 nm wavelength a) for 1 hr, b) for 1 hr followed by 14 days at room temperature in the dark, c) for 1 hr followed by 24 hrs at 60°C and d) for 1 hr followed by 5 days at 60°C.

Table S22. *E*:*Z* ratio obtained by integration of the ^{1}H NMR peaks of a 3.0 mM solution of (*E*)–L^{py} in MeOH-d_4 after illumination and heating.

Treatment	(*E*:*Z*) ratio
395 nm, 1 hr	0:100
1) 395 nm, 1 hr; 2) rt, 14 days	4:96
1) 395 nm, 1 hr; 2) 60°C, 24 hrs	25:75
1) 395 nm, 1 hr; 2) 60°C, 5 days	69:31

Ratio attributed by integration of the peaks attributed to H_{13} at 8.88 ppm (*Z*–isomer) and 8.77 ppm (*E*–isomer).

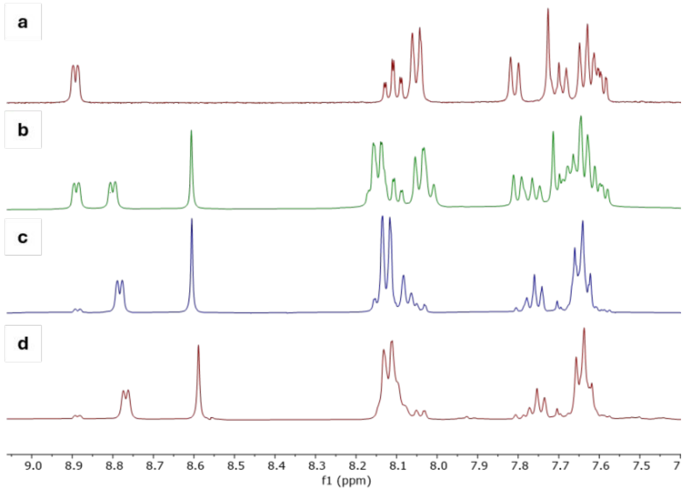

Figure S41. ^{1}H NMR (400 MHz, MeOH–d_4) of a 1.5 mM solution of **La–*E*** after illumination with a 395 nm wavelength a) for 1 hr, b) for 1 hr followed by 14 days at room temperature in the dark, c) for 1 hr followed by 24 hrs at 60°C and d) for 1 hr followed by 5 days at 60°C.

Table S23. ***E*:*Z* ratio obtained by integration of the ^{1}H NMR peaks of a 1.5 mM solution of La–*E* in MeOH-d_4 after illumination and heating.**

Treatment	(*E*:*Z*) ratio
395 nm, 1 hr	0:100
1) 395 nm, 1 hr; 2) rt, 14 days	51:49
1) 395 nm, 1 hr; 2) 60°C, 24 hrs	90:10
1) 395 nm, 1 hr; 2) 60°C, 5 days	90:10

Ratio attributed by integration of the peaks attributed to H_{13} at 8.88 ppm (*Z*–isomer) and 8.77 ppm (*E*–isomer in the **La–L^{py}** complex).

UV–Visible Illumination Kinetics Data

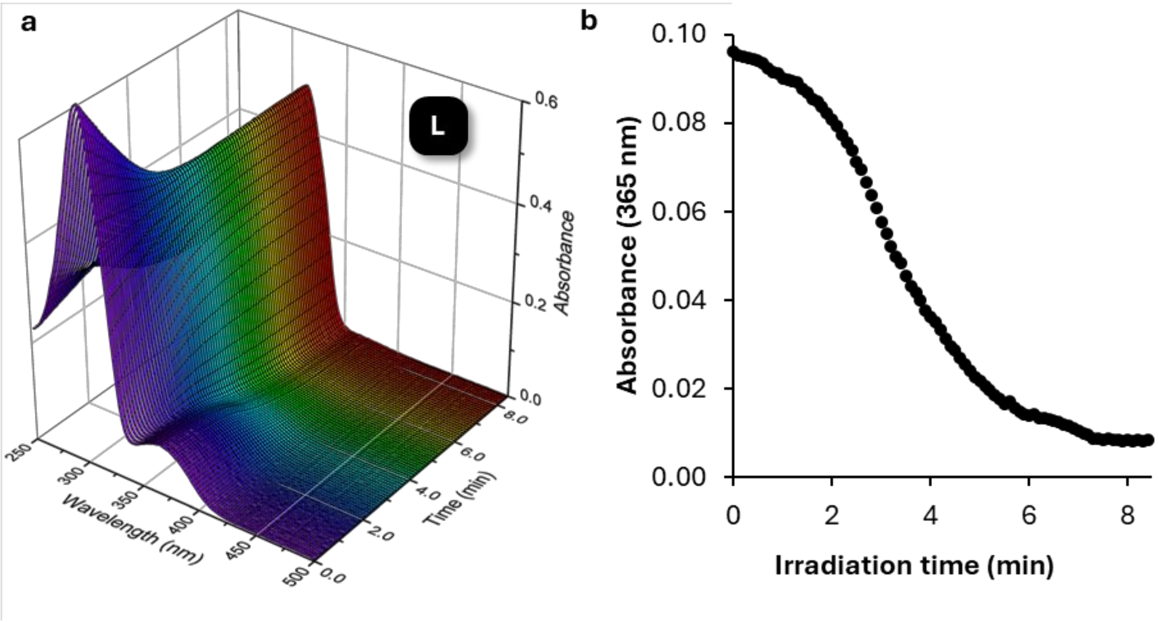

Figure S42. a) 3D plot of the illumination kinetic experiment upon illumination using a 390 nm monochromatic wavelength for **(*E*)–L**^{py} (30 µM in MeOH) reporting absorbance *vs* wavelength (250 nm – 500 nm) *vs* illumination time. b) Profile of absorbance at 365 nm *vs* illumination time for **(*E*)–L**^{py} (black trace).

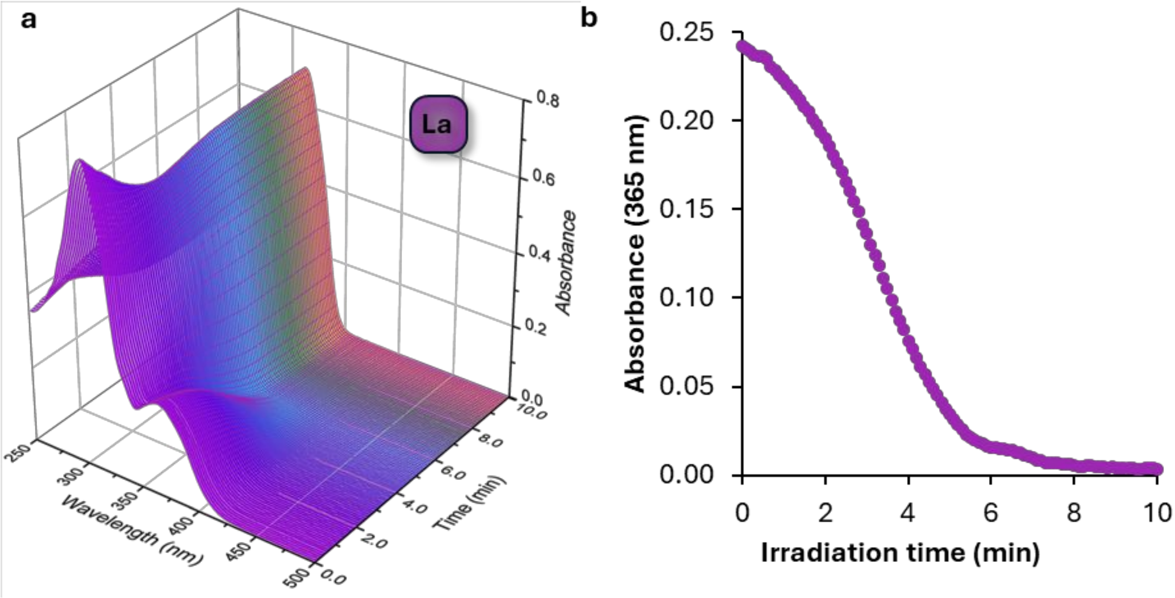

Figure S43. a) 3D plot of the illumination kinetic experiment upon illumination using a 390 nm monochromatic wavelength for **La–*E*** (15 µM in MeOH) reporting absorbance *vs* wavelength (250 nm – 500 nm) *vs* illumination time. b) Profile of absorbance at 365 nm *vs* illumination time for **La–*E*** (purple trace).

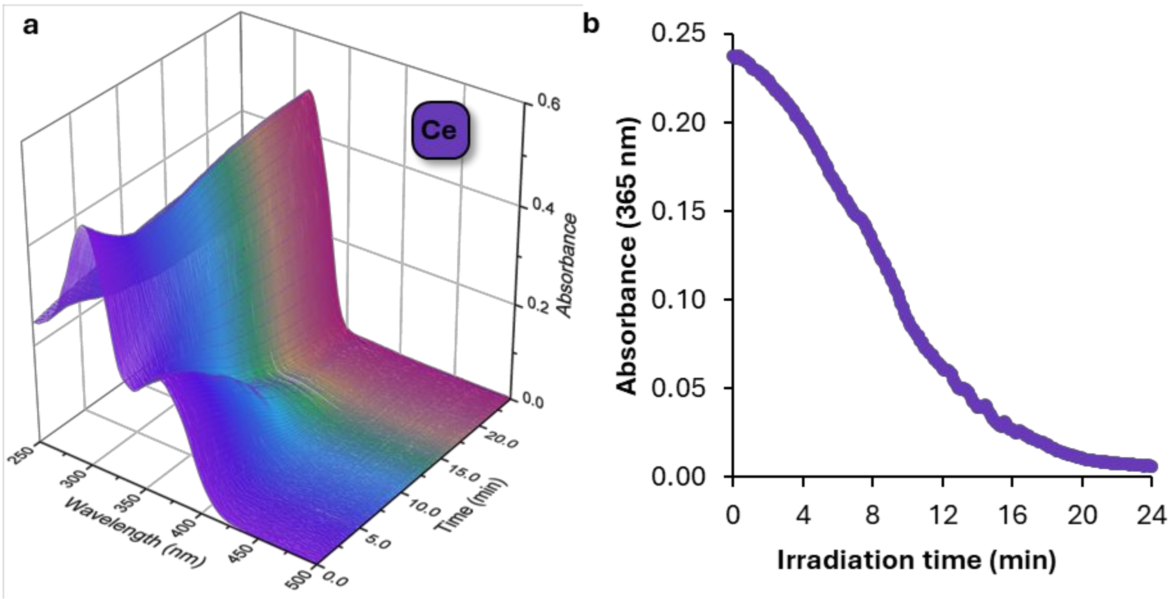

Figure S44. a) 3D plot of the illumination kinetic experiment upon illumination using a 390 nm monochromatic wavelength for **Ce–*E*** (15 µM in MeOH) reporting absorbance *vs* wavelength (250 nm – 500 nm) *vs* illumination time. b) Profile of absorbance at 365 nm *vs* illumination time for **Ce–*E*** (deep purple trace).

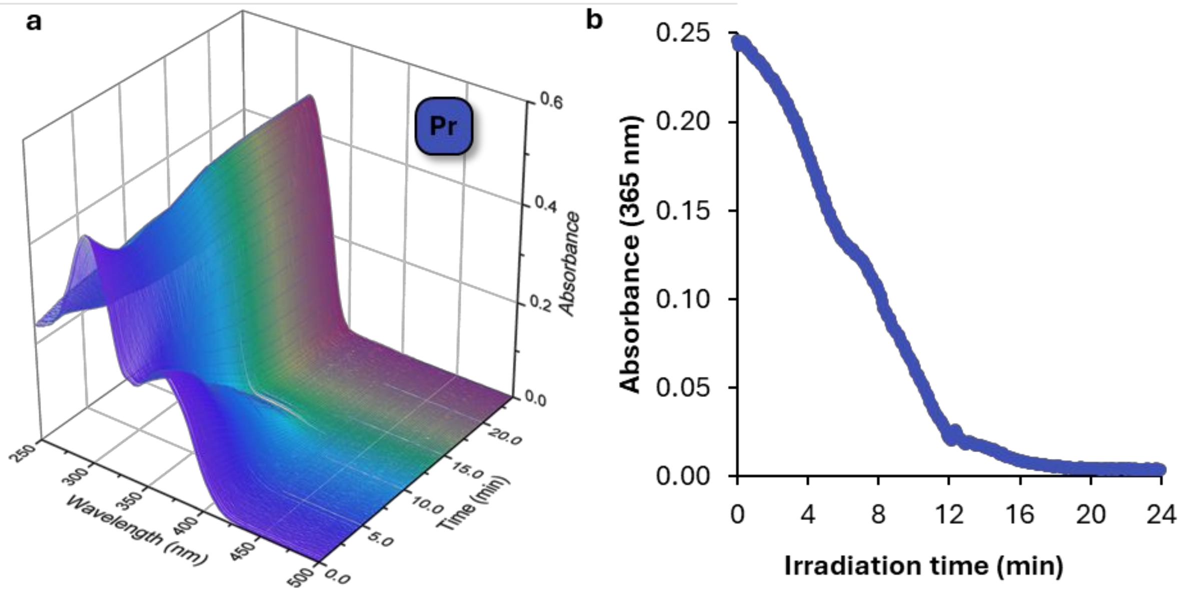

Figure S45. a) 3D plot of the illumination kinetic experiment upon illumination using a 390 nm monochromatic wavelength for **Pr–*E*** (15 µM in MeOH) reporting absorbance *vs* wavelength (250 nm – 500 nm) *vs* illumination time. b) Profile of absorbance at 365 nm *vs* illumination time for **Pr–*E*** (indigo trace).

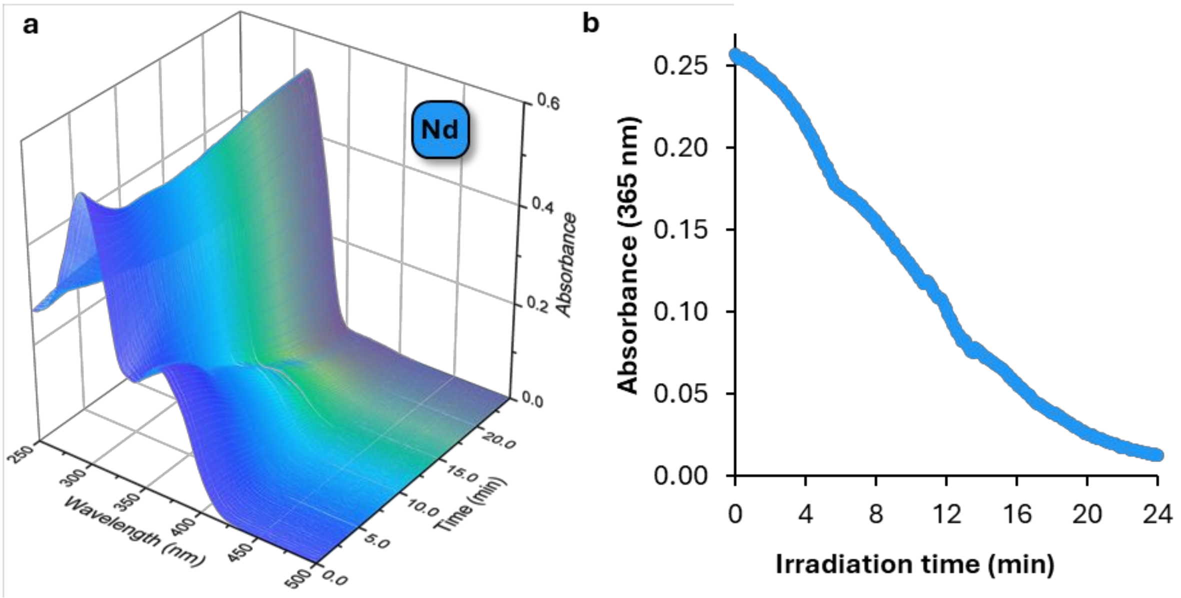

Figure S46. a) 3D plot of the illumination kinetic experiment upon illumination using a 390 nm monochromatic wavelength for **Nd–*E*** (15 µM in MeOH) reporting absorbance *vs* wavelength (250 nm – 500 nm) *vs* illumination time. b) Profile of absorbance at 365 nm *vs* illumination time for **Nd–*E*** (blue trace).

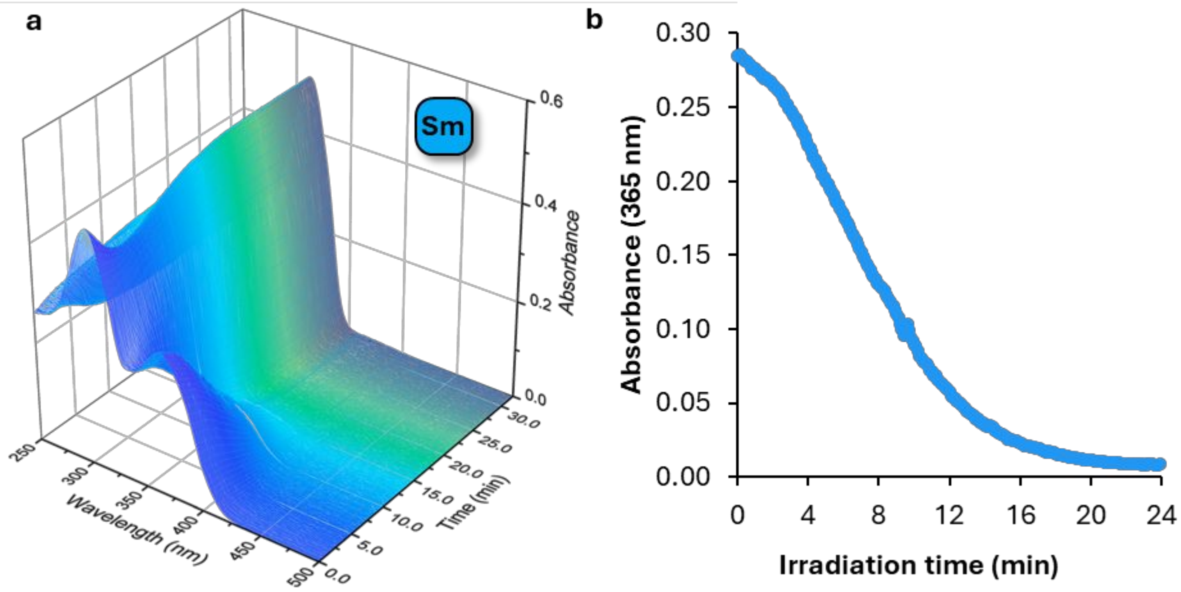

Figure S47. a) 3D plot of the illumination kinetic experiment upon illumination using a 390 nm monochromatic wavelength for **Sm–*E*** (15 µM in MeOH) reporting absorbance *vs* wavelength (250 nm – 500 nm) *vs* illumination time. b) Profile of absorbance at 365 nm *vs* illumination time for **Sm–*E*** (light blue trace).

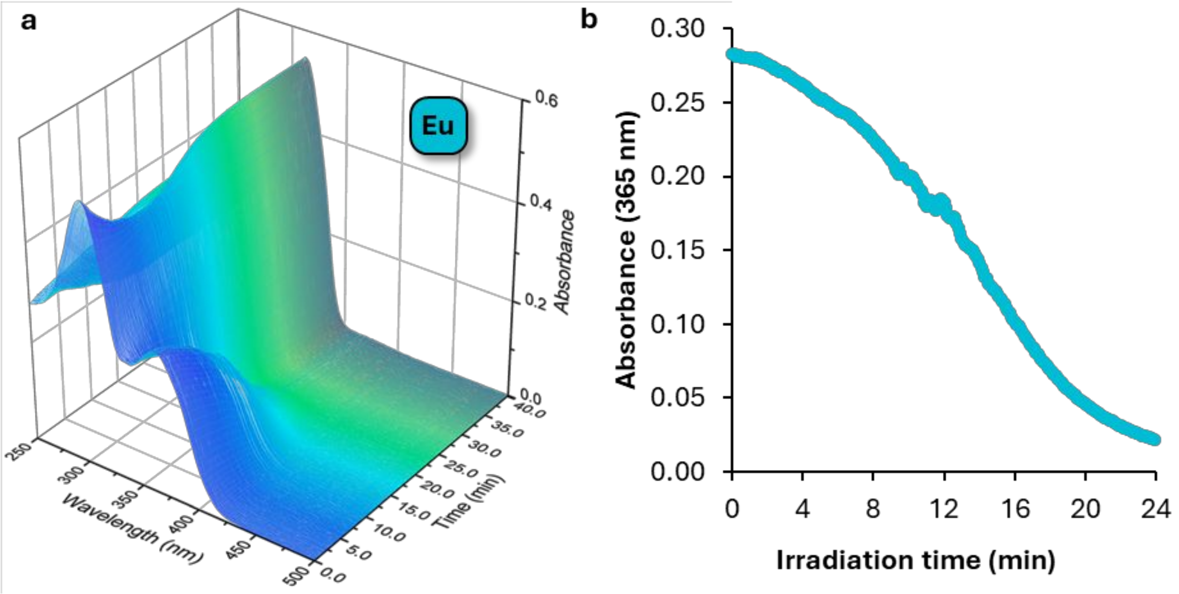

Figure S48. a) 3D plot of the illumination kinetic experiment upon illumination using a 390 nm monochromatic wavelength for **Eu–*E*** (15 µM in MeOH) reporting absorbance *vs* wavelength (250 nm – 500 nm) *vs* illumination time. b) Profile of absorbance at 365 nm *vs* illumination time for **Eu–*E*** (cyan trace).

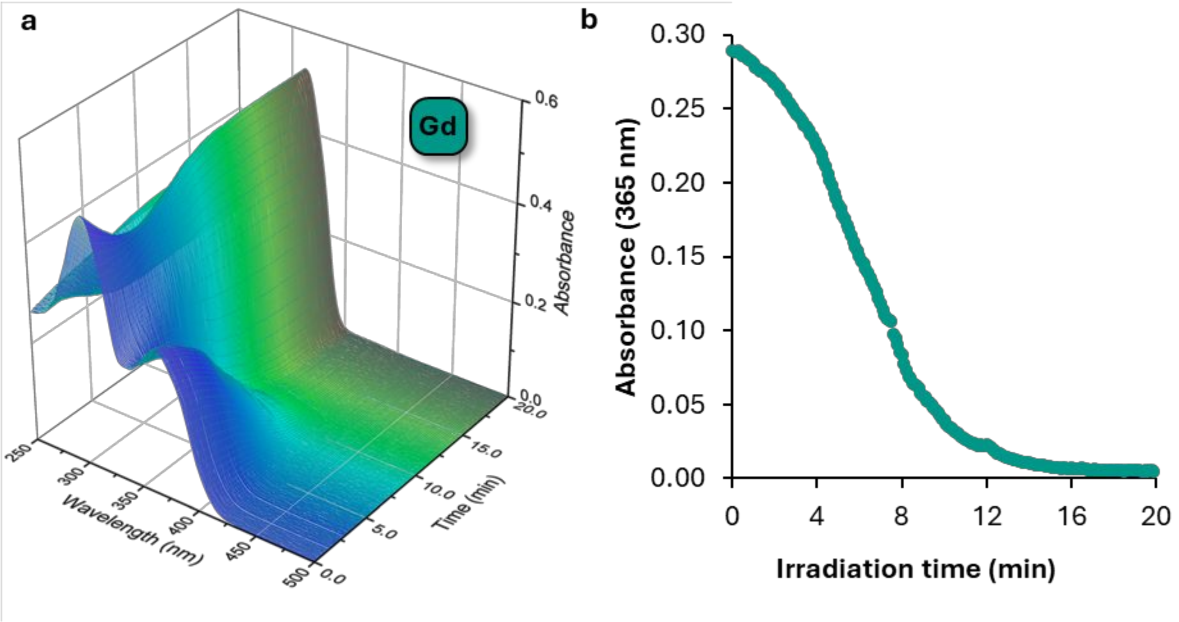

Figure S49. a) 3D plot of the illumination kinetic experiment upon illumination using a 390 nm monochromatic wavelength for **Gd–*E*** (15 µM in MeOH) reporting absorbance *vs* wavelength (250 nm – 500 nm) *vs* illumination time. b) Profile of absorbance at 365 nm *vs* illumination time for **Gd–*E*** (teal trace).

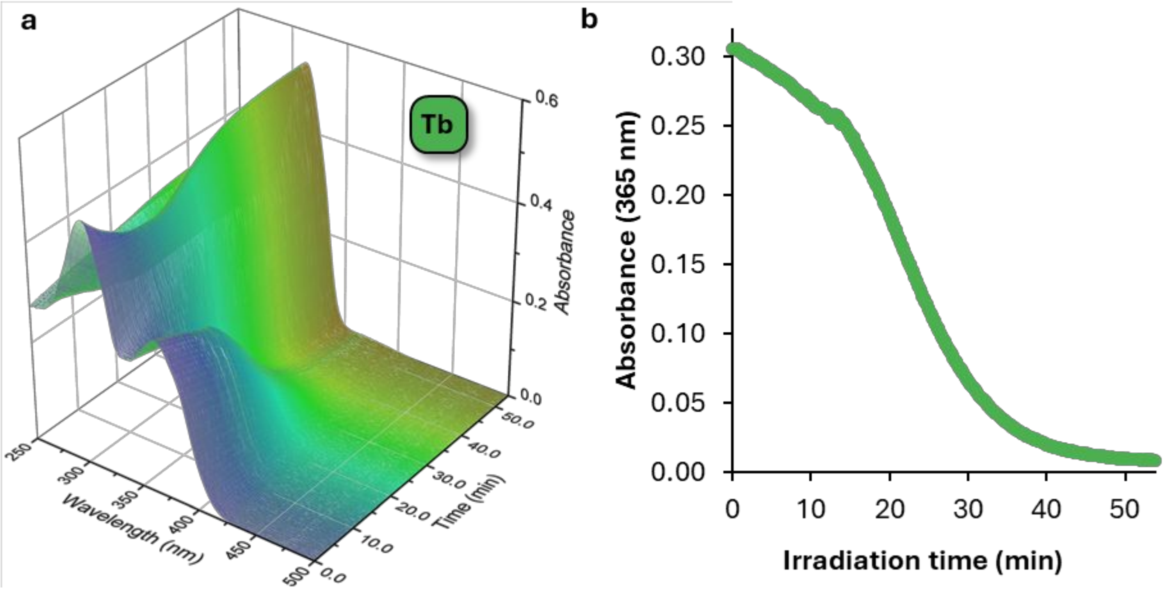

Figure S50. a) 3D plot of the illumination kinetic experiment upon illumination using a 390 nm monochromatic wavelength for **Tb–*E*** (15 µM in MeOH) reporting absorbance *vs* wavelength (250 nm – 500 nm) *vs* illumination time. b) Profile of absorbance at 365 nm *vs* illumination time for **Tb–*E*** (green trace).

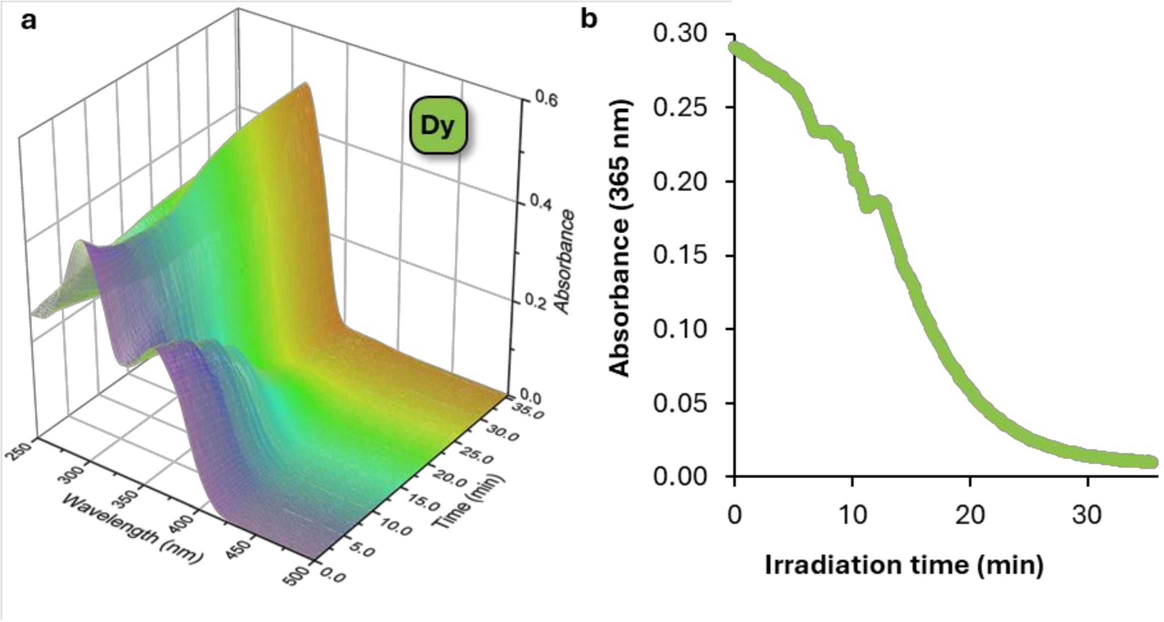

Figure S51. a) 3D plot of the illumination kinetic experiment upon illumination using a 390 nm monochromatic wavelength for **Dy–*E*** (15 µM in MeOH) reporting absorbance *vs* wavelength (250 nm – 500 nm) *vs* illumination time. b) Profile of absorbance at 365 nm *vs* illumination time for **Dy–*E*** (light green trace).

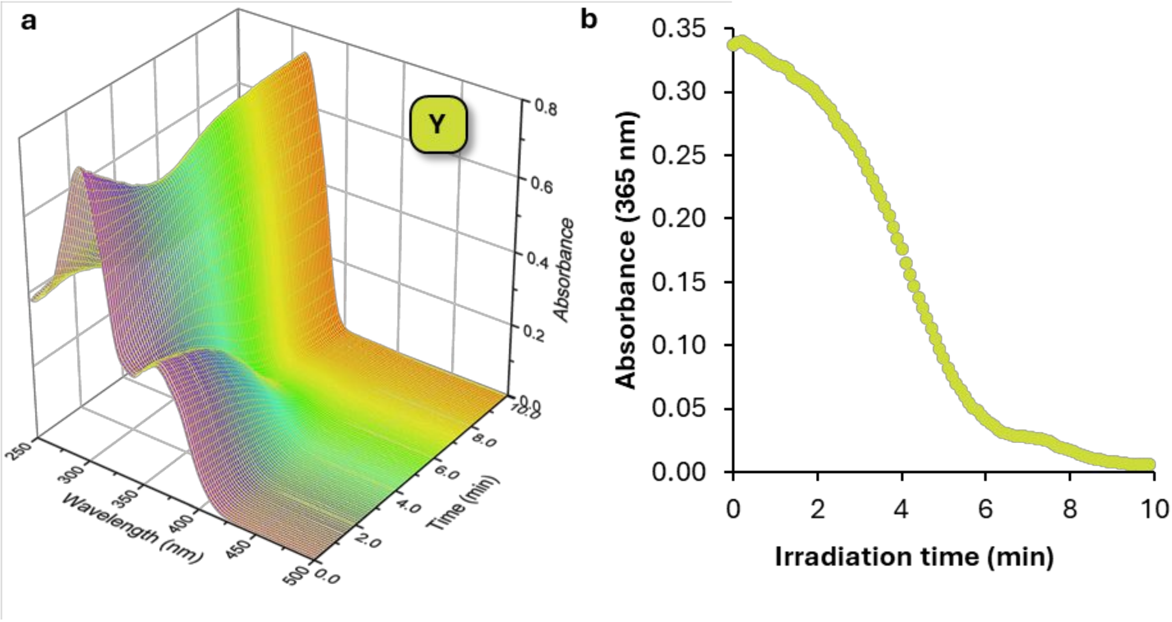

Figure S52. a) 3D plot of the illumination kinetic experiment upon illumination using a 390 nm monochromatic wavelength for **Y–*E*** (15 µM in MeOH) reporting absorbance *vs* wavelength (250 nm – 500 nm) *vs* illumination time. b) Profile of absorbance at 365 nm *vs* illumination time for **Y–*E*** (lime green trace).

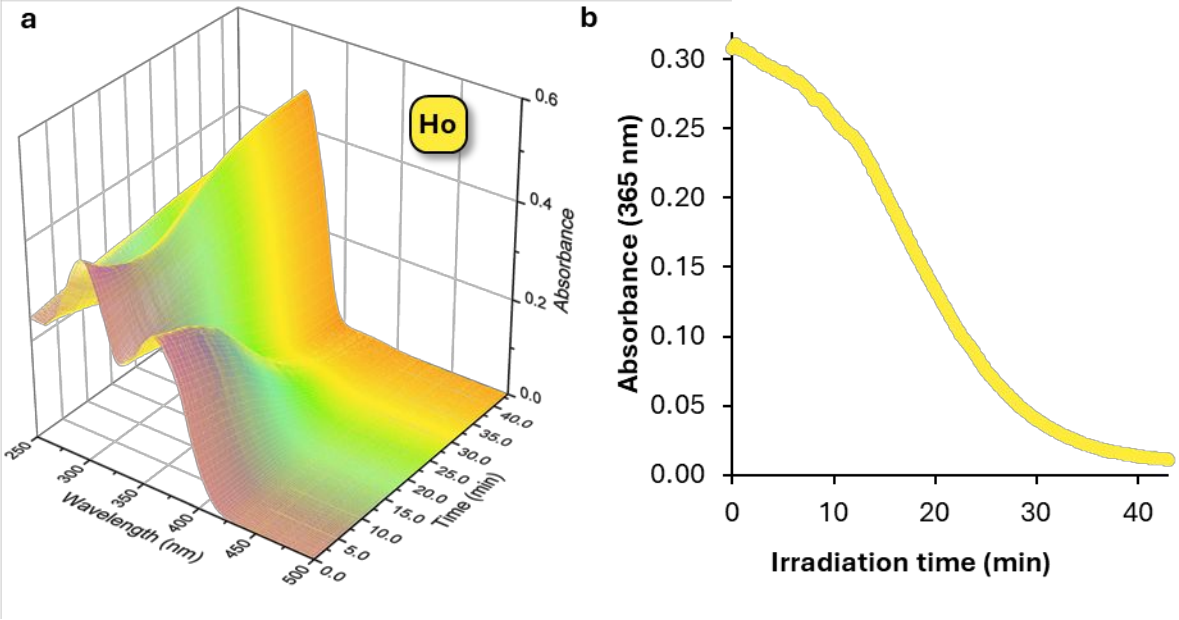

Figure S53. a) 3D plot of the illumination kinetic experiment upon illumination using a 390 nm monochromatic wavelength for **Ho–*E*** (15 µM in MeOH) reporting absorbance *vs* wavelength (250 nm – 500 nm) *vs* illumination time. b) Profile of absorbance at 365 nm *vs* illumination time for **Ho–*E*** (yellow trace).

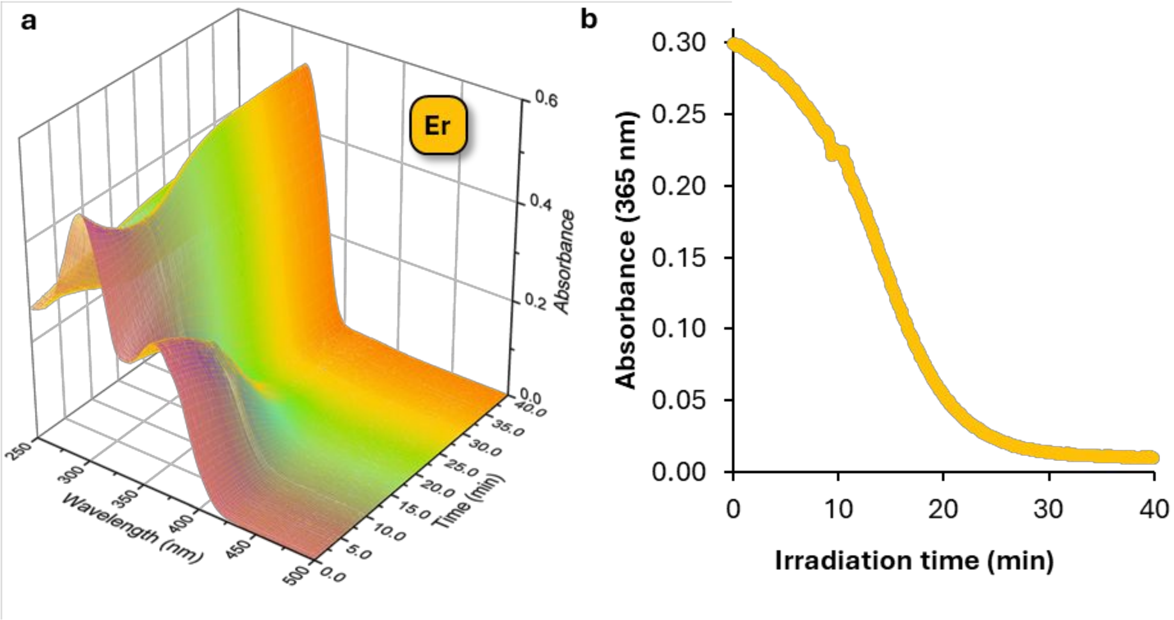

Figure S54. a) 3D plot of the illumination kinetic experiment upon illumination using a 390 nm monochromatic wavelength for **Er–*E*** (15 µM in MeOH) reporting absorbance *vs* wavelength (250 nm – 500 nm) *vs* illumination time. b) Profile of absorbance at 365 nm *vs* illumination time for **Er–*E*** (amber trace).

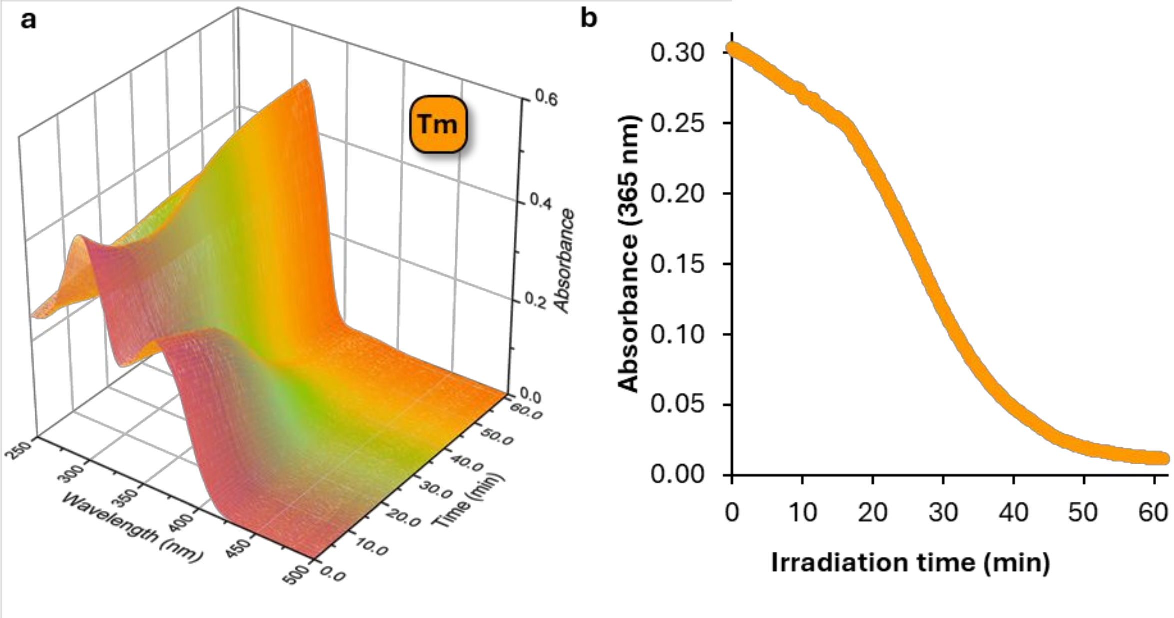

Figure S55. a) 3D plot of the illumination kinetic experiment upon illumination using a 390 nm monochromatic wavelength for **Tm–*E*** (15 µM in MeOH) reporting absorbance *vs* wavelength (250 nm – 500 nm) *vs* illumination time. b) Profile of absorbance at 365 nm *vs* illumination time for **Tm–*E*** (orange trace).

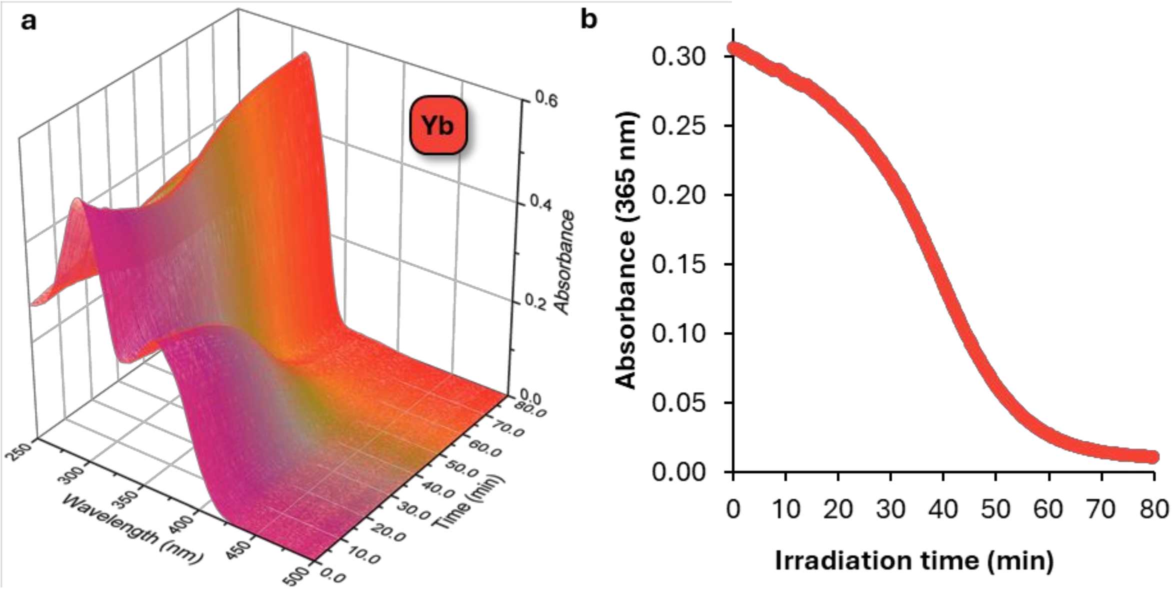

Figure S56. a) 3D plot of the illumination kinetic experiment upon illumination using a 390 nm monochromatic wavelength for **Yb–*E*** (15 µM in MeOH) reporting absorbance *vs* wavelength (250 nm–500 nm) *vs* illumination time. b) Profile of absorbance at 365 nm *vs* illumination time for **Yb–*E*** (red trace).

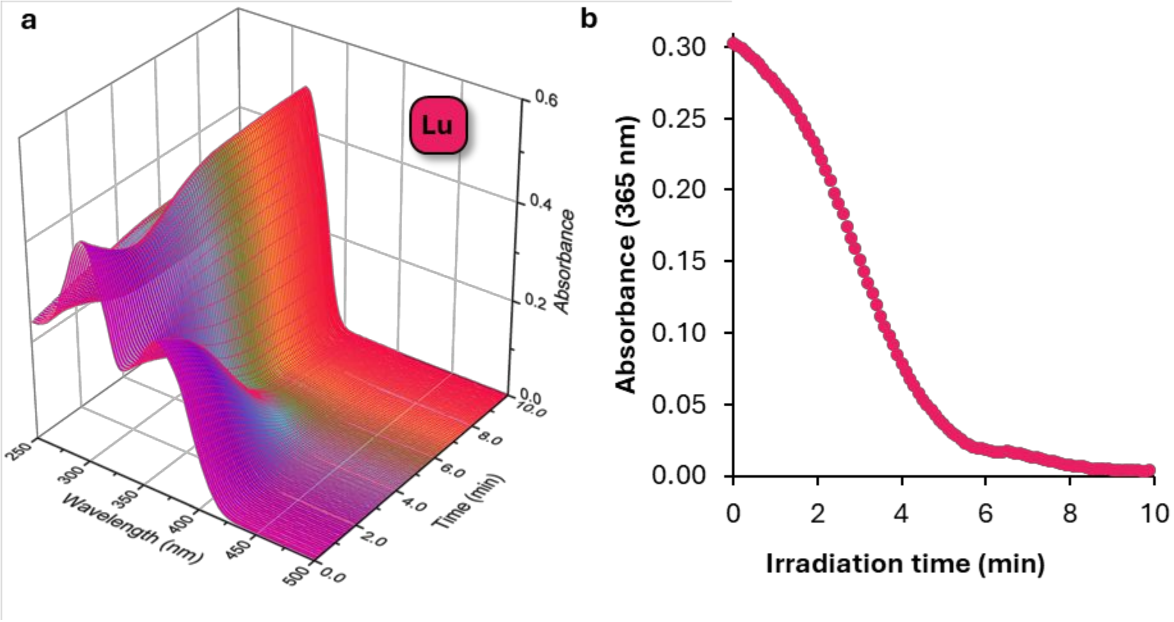

Figure S57. a) 3D plot of the illumination kinetic experiment upon illumination using a 390 nm monochromatic wavelength for **Lu–*E*** (15 µM in MeOH) reporting absorbance *vs* wavelength (250 nm – 500 nm) *vs* illumination time. b) Profile of absorbance at 365 nm *vs* illumination time for **Lu–*E*** (ruby trace).

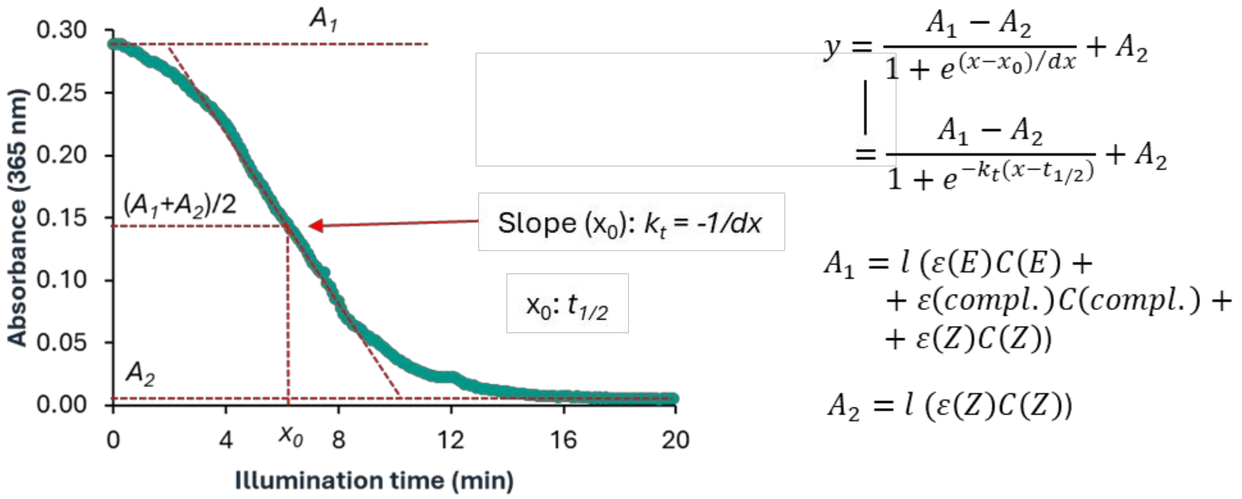

Figure S58. Boltzmann Sigmoidal Fitting for the photokinetic experiment with **Gd–*E*** (15 µM in MeOH). The fitting equation is reported along with the fitting parameters: with A_1 being the absorbance at the start of the experiment, A_2 the absorbance at the end of the experiment, x_0 ($t_{1/2}$) the time after which $y = (A_1+A_2)/2$, and k_t the photodissociation parameter defining the slope of the curve at x_0.

Table S24. Fitting parameters for the illumination kinetics of (*E*)–L^{py} and RE–*E* using a 390 nm monochromatic wavelength and fitted using the Boltzmann Sigmoidal curve. Standard deviations are reported in parentheses and refer to the last significant digit.

compound	A_1	A_2	$t_{1/2}$ (min)	dx (min)	k_t (min^{-1})	R^2
**La–*E***	0.250(4)	0.004(1)	3.1(1)	0.98(4)	-1.02(4)	0.9786
**Ce–*E***	0.265(3)	0.0043(8)	7.70(1)	3.46(6)	-0.289(5)	0.9942
**Pr–*E***	0.280(4)	0.0008(8)	6.0(1)	2.97(7)	-0.336(8)	0.9842
**Nd–*E***	0.32(1)	-0.002(2)	6.3(3)	4.3(2)	-0.23(1)	0.9684
**Sm–*E***	0.29(3)	0.004(7)	9.2(1)	3.70(7)	-0.271(5)	0.9903
**Eu–*E***	0.286(2)	0.0063(7)	13.4(1)	3.89(7)	-0.257(5)	0.9815
**Gd–*E***	0.304(2)	0.0052(7)	5.9(1)	1.99(4)	-0.50(1)	0.9936
**Tb–*E***	0.3031(5)	0.0080(4)	22.2(1)	5.57(3)	-0.180(1)	0.9981
**Dy–*E***	0.295(2)	0.0081(9)	13.7(1)	4.20(8)	-0.238(5)	0.9888
**Y–*E***	0.339(3)	0.009(2)	4.0(1)	0.96(3)	-1.04(3)	0.9895
**Ho–*E***	0.3183(9)	0.0083(5)	18.0(1)	5.49(5)	-0.182(2)	0.9961
**Er–*E***	0.301(1)	0.0097(5)	13.7(1)	3.74(5)	-0.268(4)	0.9915
**Tm–*E***	0.3028(8)	0.0095(5)	26.3(1)	7.15(6)	-0.140(1)	0.9960
**Yb–*E***	0.3010(8)	0.0060(6)	37.5(1)	8.98(9)	-0.111(1)	0.9930
**Lu–*E***	0.316(1)	0.0060(3)	2.9(1)	0.97(7)	-1.03(7)	0.9992
**(*E*)–L^{py}**	0.0993(6)	0.0087(4)	3.2(1)	0.96(2)	-1.04(2)	0.9964

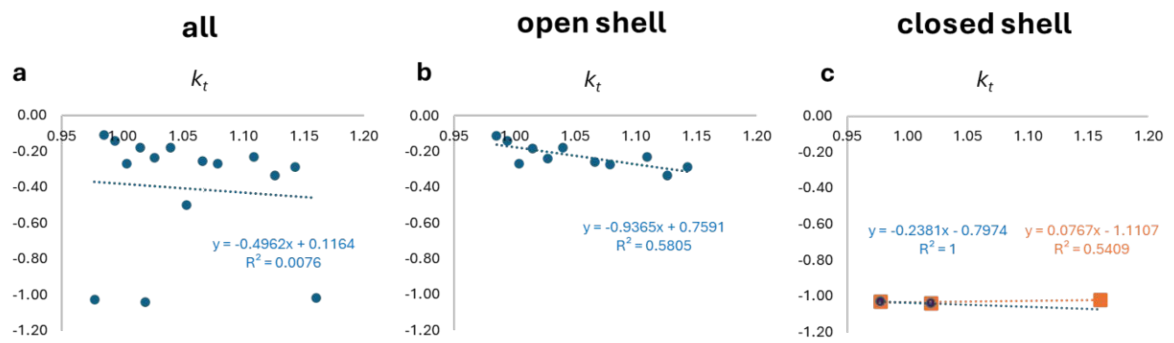

Figure S59. k_t (min^{-1}) plotted against RE ionic radii (Å) for a) the entire series of REs, b) open shell REs, c) closed shell REs. Trend lines are reported on the graphs along with the respective equations and R^2 values. In the case of section c), orange trend lines refer to La^{3+}, Y^{3+} and Lu^{3+}, while blue trend

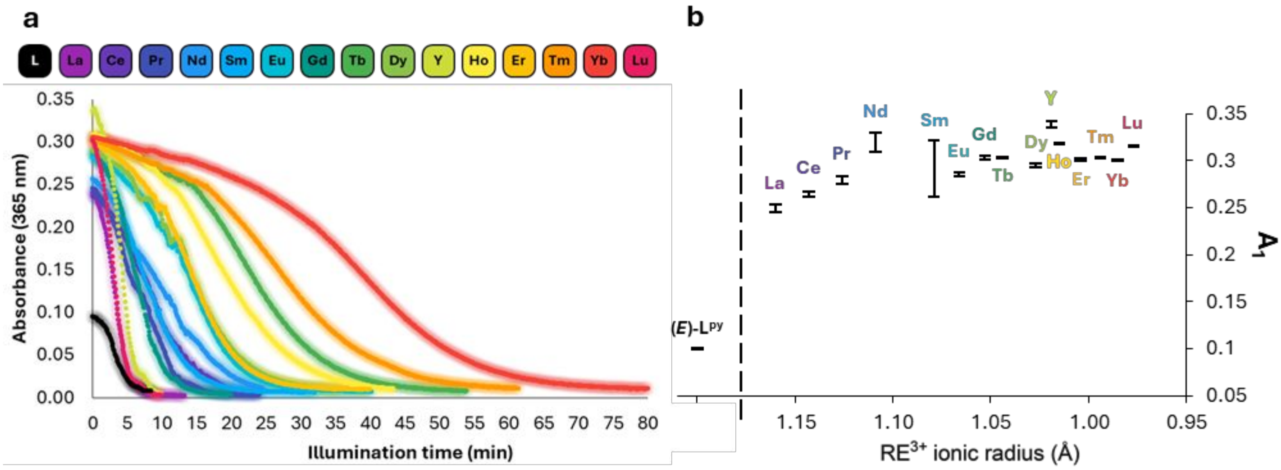

Figure S60. a) Experimental absorbances at 365 nm during the photokinetic experiments for **RE–*E*** complexes (15 µM) and **(*E*)–L^{py}** (30 µM) upon irradiation with a 390 nm monochromatic wavelength in MeOH at room temperature. b) $\boldsymbol{A_1}$ fitting parameter obtained for the photokinetic experiments versus RE^{III} ionic radii.

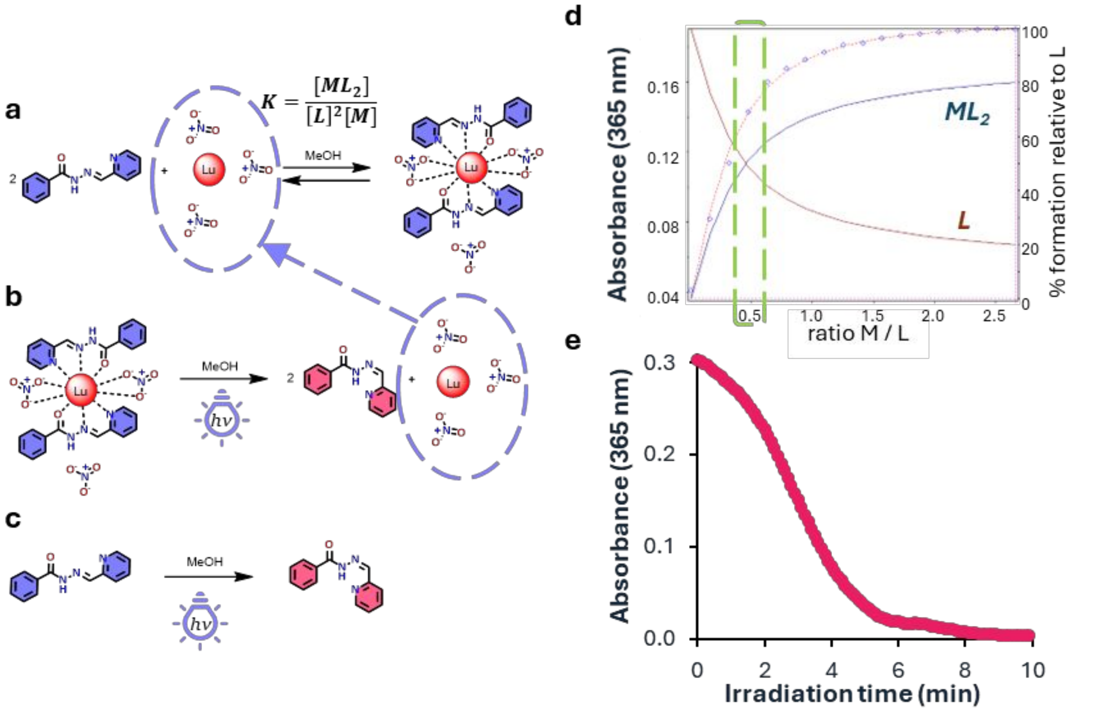

Figure S61. a) Equilibrium reaction for the formation of **Lu-*E*** complex. b) Photodissociation of **Lu-*E*** complex upon irradiation using a 390 nm monochromatic radiation. c) Isomerization of the **(*E*)-L^{py}** ligand upon irradiation using a 390 nm monochromatic radiation. d) Fitting plots of UV–Visible titrations of 15 µM **(*E*)–L^{py}** with $Lu(NO_3)_3$ in MeOH monitoring the absorbance at 365 nm. Blue diamonds indicate experimental values, and red dotted lines indicate the absorbance derived from the fitting process. The M:L ratio present in the photokinetic experiments was circled in green, highlighting the present of a large amount of residual free ligand in solution. d) Profile of absorbance at 365 nm *vs* illumination time for **Lu–*E*** 15 µM in MeOH (ruby trace).

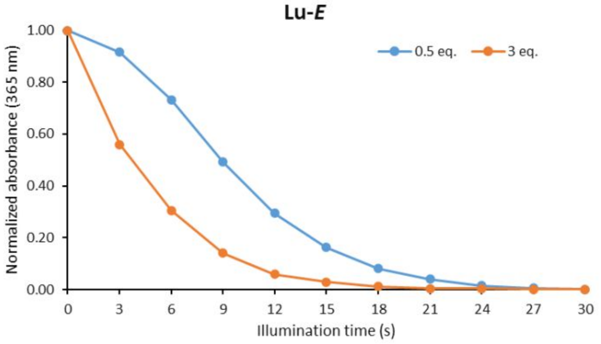

Figure S62. Normalized absorbance at 365 nm *vs* illumination time using the 395 nm photoreactor for two 15 µM solution of **(*E*)-L^{py}** in MeOH, after addition of 0.5 (stoichiometric amount) or 3 eq. (excess) of $Lu(NO_3)_3$. In the experiment with the stoichiometric amount of $Lu(NO_3)_3$, the sigmoidal shape of the decrease in absorbance is still visible, whereas in the case of the experiment conducted in excess of $Lu(NO_3)_3$, the decrease follows a more traditional zero-order reaction kinetic.

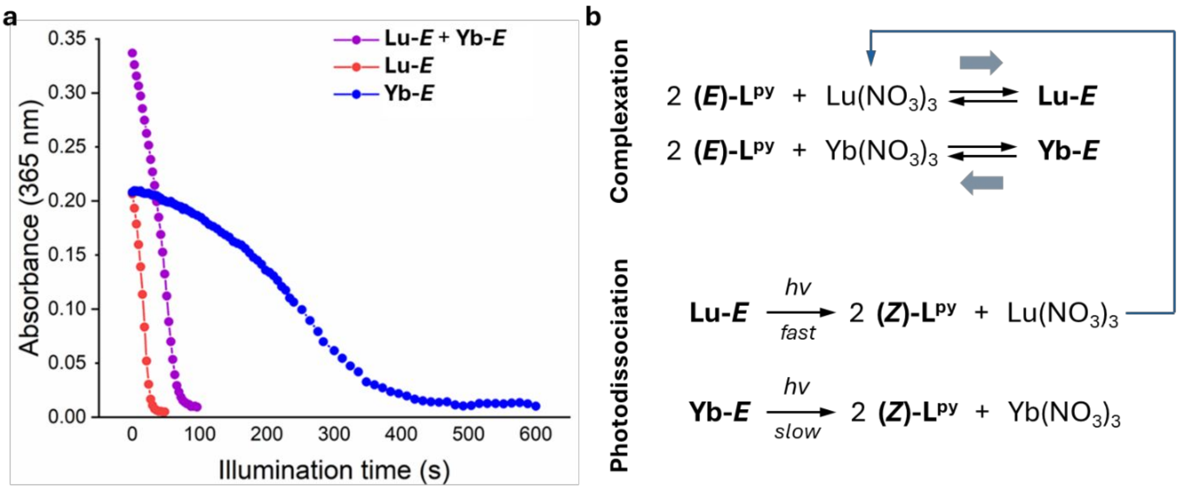

Figure S63. a) Absorbance at 365 nm for the illumination of **Yb-*E*** (15 µM in MeOH, blue dots), **Lu-*E*** (15 µM in MeOH, red dots), and 1:1 mixture of **Yb-*E*** of **Lu-*E*** (both 15 µM in MeOH, purple dots) using the 395 nm photoreactor. b) Proposed reaction schemes for the illumination of a mixture of **Yb-*E*** and **Lu-*E*** highlighting the complexation and photodissociation reactions, as well as the effect produced by the photodissociation of the **Lu-*E*** complex (fast photodissociation) on the system. The isomerization of the free **(*E*)-L**^{py} ligand was omitted for clarity.

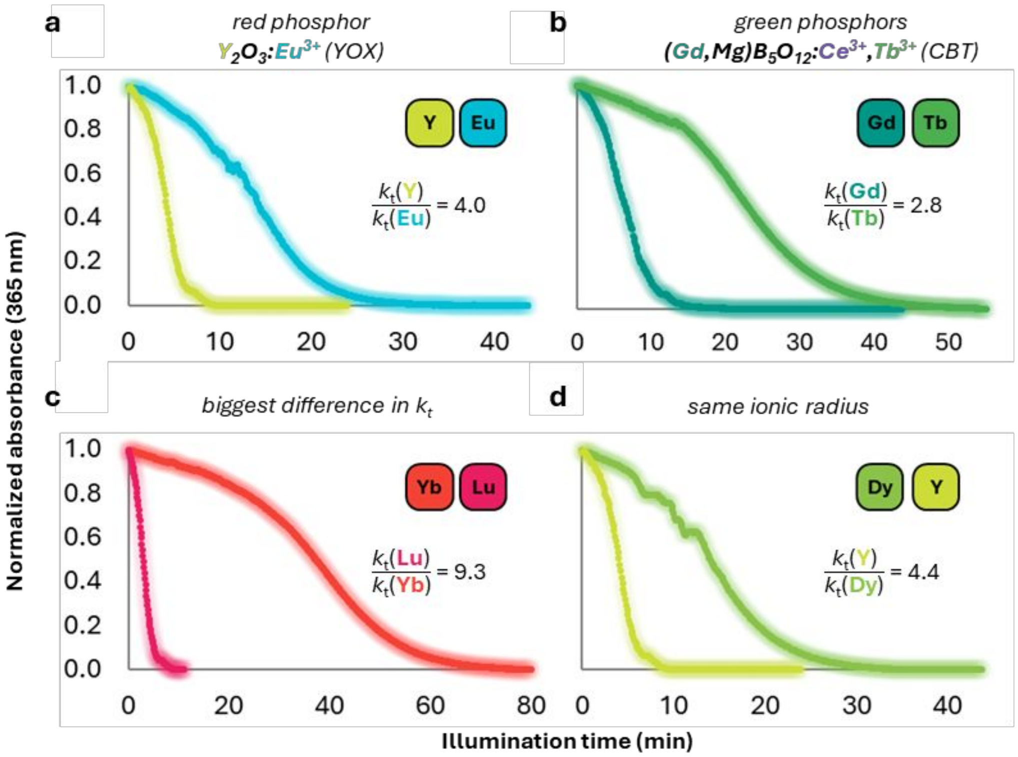

Figure S64. Photokinetic traces at 365 nm for selected **RE–*E*** pairs (15 µM in MeOH): a) Y/Eu, b) Gd/Tb, c) Yb/Lu and d) Dy/Y. The ratio between the photodissociation parameters (k_t) and the industrial application of mixtures of these ions are highlighted.

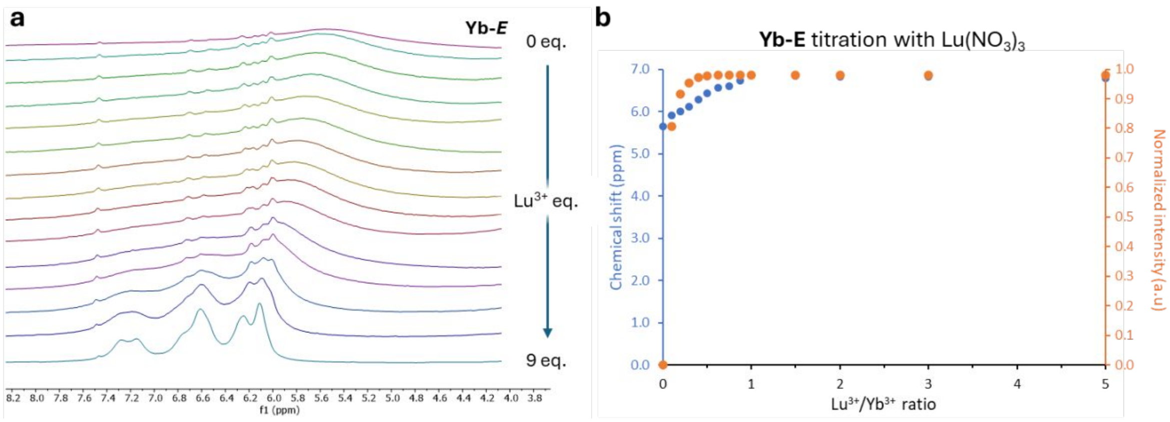

Figure S65. a) ^{1}H NMR (400 MHz, MeOH-d_4) titration of 5.5 mM **Yb-*E*** using $Lu(NO_3)_3$ with focus on the region 4.0–8.2 ppm showing the increase in intensity and sharpening of the **Lu-*E*** resonances. b) Chemical shift of the maximum of the broad resonance at 5.6 ppm in the spectra of **Yb-*E*** highlighting the change in its chemical shift and increase in intensity (obtained by integration of the ±1 ppm region around the peak).

Photophysical Characterization of RE–complexes

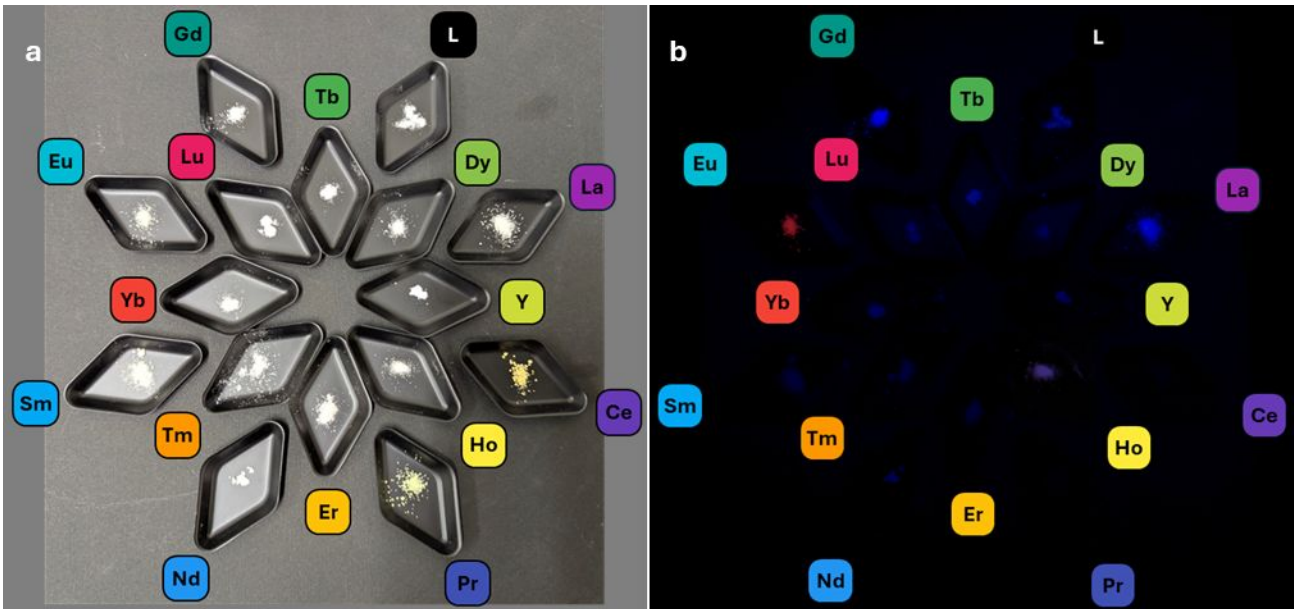

Figure S66. Picture of **RE–*E*** complexes under a) ambient light and b) under illumination with a 365 nm TLC lamp. The red emission of **Eu–*E*** and the blue ligand residual fluorescence for **La–*E***, **Gd–*E***, **Y–*E*** and **Lu–*E*** are clearly visible.

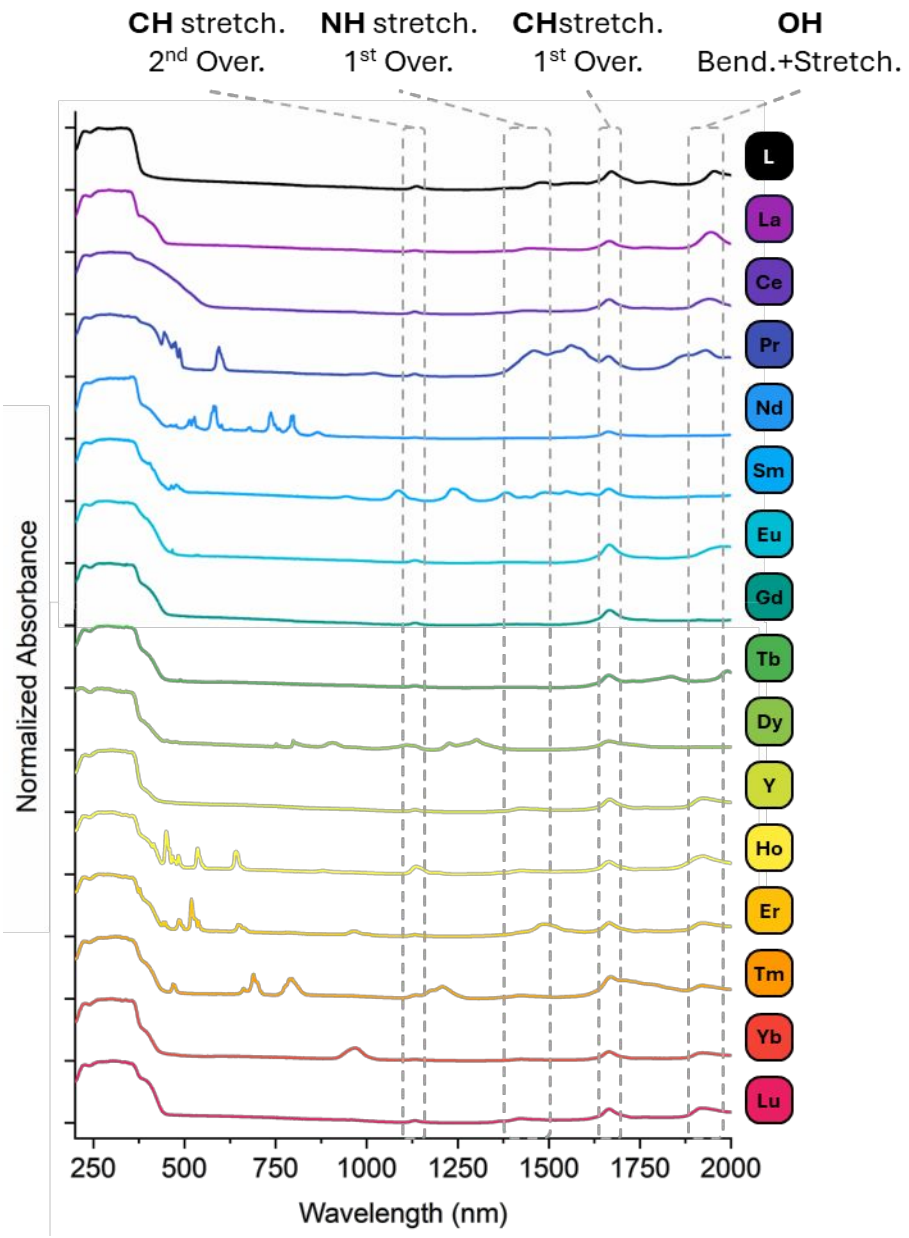

Figure S67. Normalized solid state absorbances of **(*E*)–L**^{py} and the entire **RE–*E*** series of complexes. The regions containing the overtone and combination stretching resonances are highlighted by dotted lines. [16]

Table S25. Energies of overtone stretching bands, combination stretching + bending resonances for RE–*E* complexes.

**Vibrational mode**	**Energy (cm^{-1})**	**Wavelength (nm)**
**CH** 2nd Overtone Stretch.	8826	1133
**NH** 1st Overtone Stretch.	6878	1454
**CH** 1st Overtone Stretch.	6002	1666
**OH** Bend. + Stretch.	5123	1952

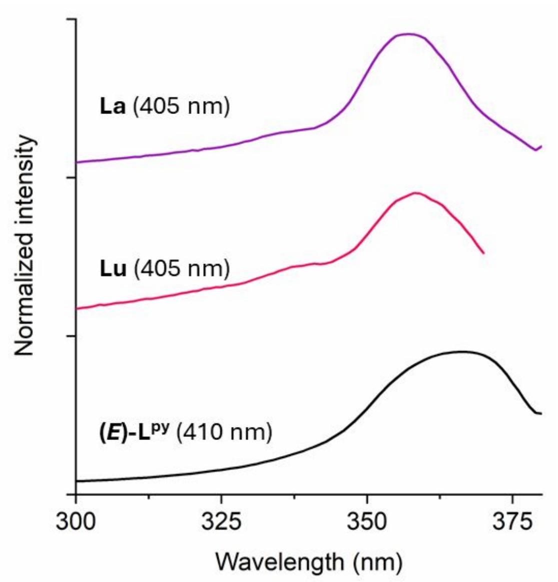

Figure S68. Normalized excitation spectra of diamagnetic **RE–*E*** complexes and **(*E*)–L**^{py} in the solid state at room temperature. The emission wavelengths used to monitor the experiments were selected based on the most intense main emission bands found experimentally and are reported in parentheses.

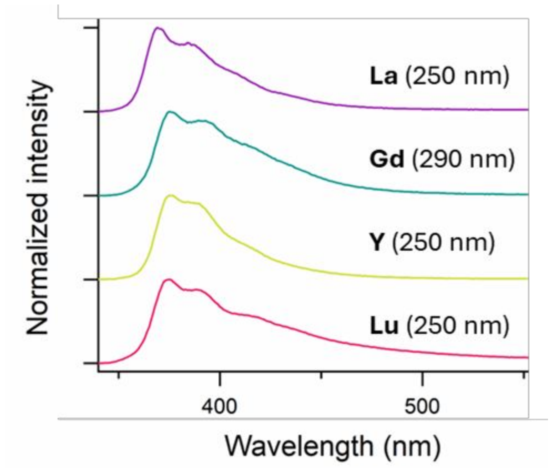

Figure S69. Normalized emission spectra of diamagnetic **RE–*E*** complexes and **(*E*)–L**^{py} in the solid state at room temperature. Excitation wavelengths used to monitor the experiments are reported in parentheses.

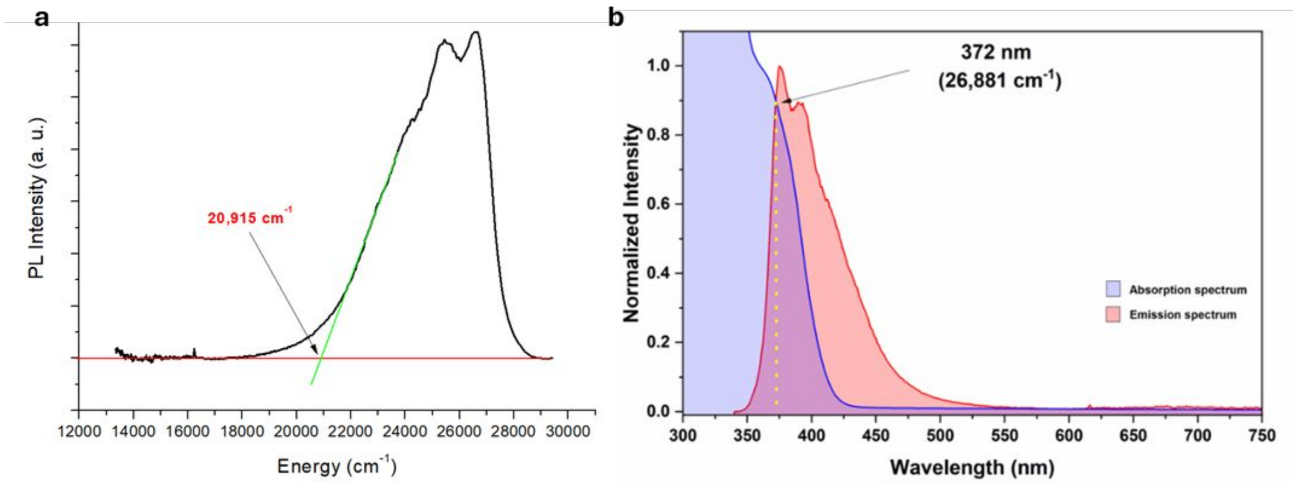

Figure S70. a) Estimation of ligand's triplet energy from the fluorescence spectrum of **Gd–*E*** in the solid state at room temperature. b) S_1 excited state energy for the **Gd-*E*** complex obtained by the intersection of the solid state absorption and emission spectra.

Table S26. Assignment of observed 4f PL transitions in the investigated **RE–*E*** complexes.

RE ion	Transition (nm, cm^{-1})			
Pr^{III}	$^1D_2 \rightarrow {}^3H_4$ (605, 16500)	$^3P_0 \rightarrow {}^1G_4$ (weak) (883, 11325)	$^1D_2 \rightarrow {}^3F_{3,4}$ (1025, 9756)	$^1D_2 \rightarrow {}^1G_4$ (1428, 7002)
Nd^{III}	$^4F_{3/2} \rightarrow {}^4I_{9/2}$ (880, 11360)	$^4F_{3/2} \rightarrow {}^4I_{11/2}$ (1064, 9430)	$^4F_{3/2} \rightarrow {}^4I_{13/2}$ (1330, 7520)	
Sm^{III}	$^4G_{5/2} \rightarrow {}^6H_{5/2}$ (565, 17700)	$^4G_{5/2} \rightarrow {}^6H_{7/2}$ (606, 16500)	$^4G_{5/2} \rightarrow {}^6H_{9/2}$ (658, 15200)	$^4G_{5/2} \rightarrow {}^6H_{11/2}$ (720, 13900)
Eu^{III}	$^5D_0 \rightarrow {}^7F_1$ (593, 16850)	$^5D_0 \rightarrow {}^7F_2$ (616, 16200)	$^5D_0 \rightarrow {}^7F_3$ (654, 15300)	$^5D_0 \rightarrow {}^7F_4$ (704, 14200)
Ho^{III}	$^5F_5 \rightarrow {}^5I_8$ (645, 15503)	$^5F_5 \rightarrow {}^5I_7$ (990, 10100)	$^5I_6 \rightarrow {}^5I_8$ (1150, 8700)	$^5F_4 \rightarrow {}^5I_4$ (1481, 6750)
Er^{III}	$^4I_{11/2} \rightarrow {}^4I_{15/2}$ (weak) (980, 10200)	$^4I_{13/2} \rightarrow {}^4I_{15/2}$ (1520, 6578)		
Tm^{III}	$^3H_4 \rightarrow {}^3H_6$ (800, 12500)	$^3H_5 \rightarrow {}^3H_6$ (980, 10200)	$^3H_4 \rightarrow {}^3F_4$ (1470, 6800)	
Yb^{III}	$^2F_{5/2} \rightarrow {}^2F_{7/2}$ (980, 10200)			

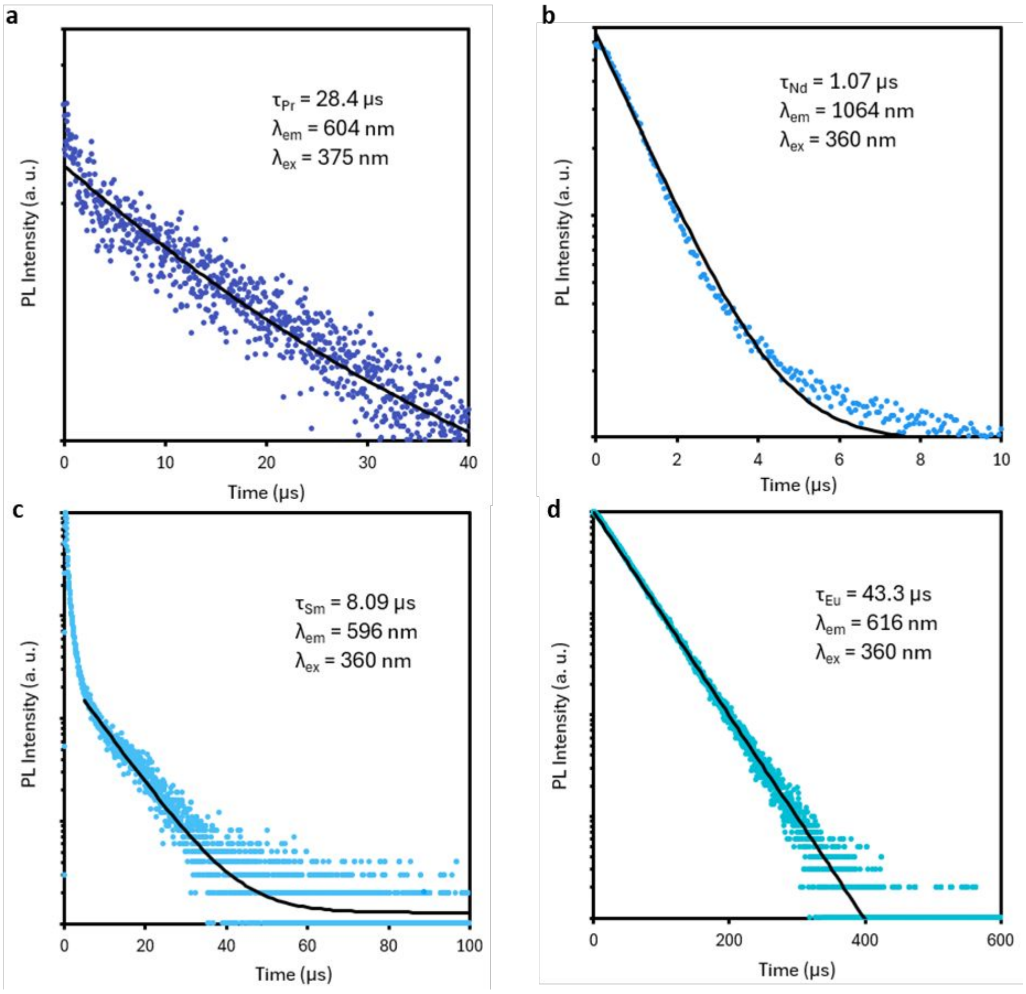

Figure S71. Time–resolved PL decay curves (dots) of crystals of a) **Pr–*E***, b) **Nd–*E***, c) **Sm–*E***, d) **Eu–*E*** reported on the logarithmic scale. Solid black lines represent the best monoexponential fit to data. Retrieved decay time constants (τ), emission and excitation wavelengths (λ) are reported in the inset. For the case of **Sm–*E*** in panel c) two decay components are clearly recognizable. The faster decay component, falling below instrumental temporal resolution, is attributed to the spectral tail of residual ligand emission and has not been included in the fitting.

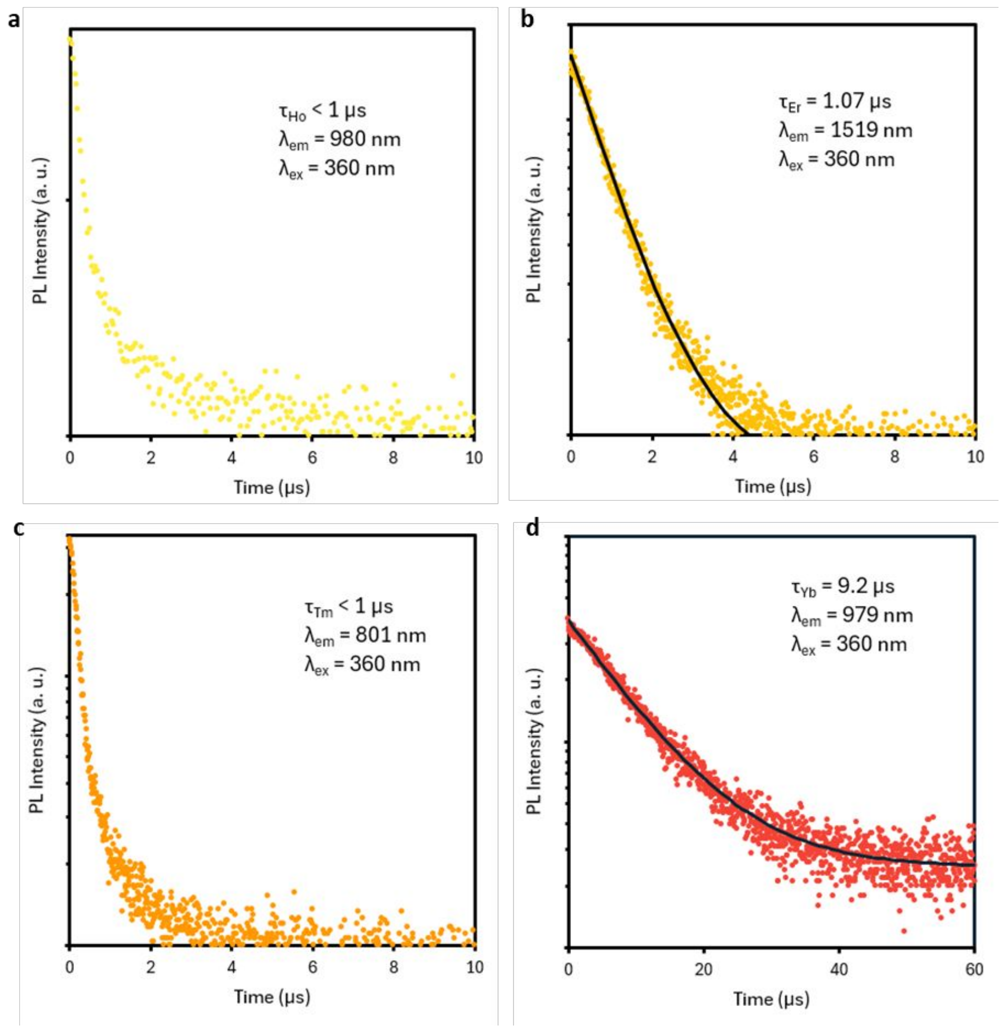

Figure S72. Time–resolved PL decay curves (dots) of crystals of a) **Ho–*E***, b) **Er–*E***, c) **Tm–*E***, d) **Yb–*E*** reported on the logarithmic scale. Solid black lines represent the best monoexponential fit to data. Retrieved decay time constants (τ), emission and excitation wavelengths (λ) are reported in the inset. The decay dynamics for **Ho–*E*** and **Tm–*E*** is in the submicrosecond range, at the limit of instrumental temporal resolution, and data have not been fitted.

Table S27. Fitting parameters for mono–exponential decays of RE–centered emission lines in RE–*E* complexes.

RE	λ_{em} (nm)	τ (μs)	Error	Adj. R–Square
**Pr–*E***	604	28.4	1.7	0.914
**Nd–*E***	1064	1.07	0.01	0.994
**Sm–*E***	596	8.09	0.06	0.973
**Eu–*E***	616	43.3	0.1	0.996
**Er–*E***	1519	1.072	0.003	0.991
**Yb–*E***	979	9.22	0.04	0.989

Ligand–to–metal energy transfer efficiency.

Residual ligand's fluorescence spectra on **RE–*E*** complexes in the solid state was acquired on tightly packed 0.2 mm–thick pellets of the pure crystalline samples ensuring the same illumination spot size and measurement conditions (excitation wavelength and power, slit size, temperature, sample orientation, filters) for each sample. Under these precautions, and in view of the isostructurality of the complexes, it is possible to semiquantitatively compare the emission spectral intensities. As described in the text, **Gd–*E*** was taken as suitable reference to estimate the ligand–to–metal energy transfer efficiency. Although the sub–nanosecond decay dynamics of the ligand, below instrumental temporal resolution, hampered a rigorous analysis through time–resolved data, ligand–to–metal energy transfer efficiency (η_{ET}) was reasonably estimated through steady state data using the formula:

$$\eta_{ET} = 1 - \frac{I_{RE}}{I_{Gd}} \qquad \textbf{(S1)}$$

Where I_{Gd} and I_{RE} are the integrated fluorescence spectral intensities of the reference **Gd–*E*** and the **RE–*E*** complexes, respectively.

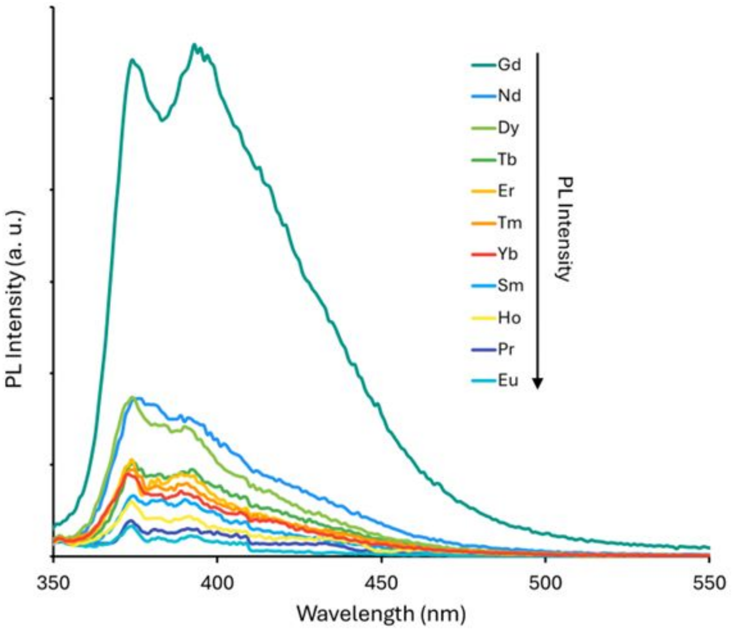

Figure S73. PL spectra of the residual ligand fluorescence of **RE–*E*** complexes with RE–centered emission compared with the optically silent **Gd–*E*** complex as reference. Color coding for the different RE complexes is reported in the legend. Excitation wavelength was 300 nm.

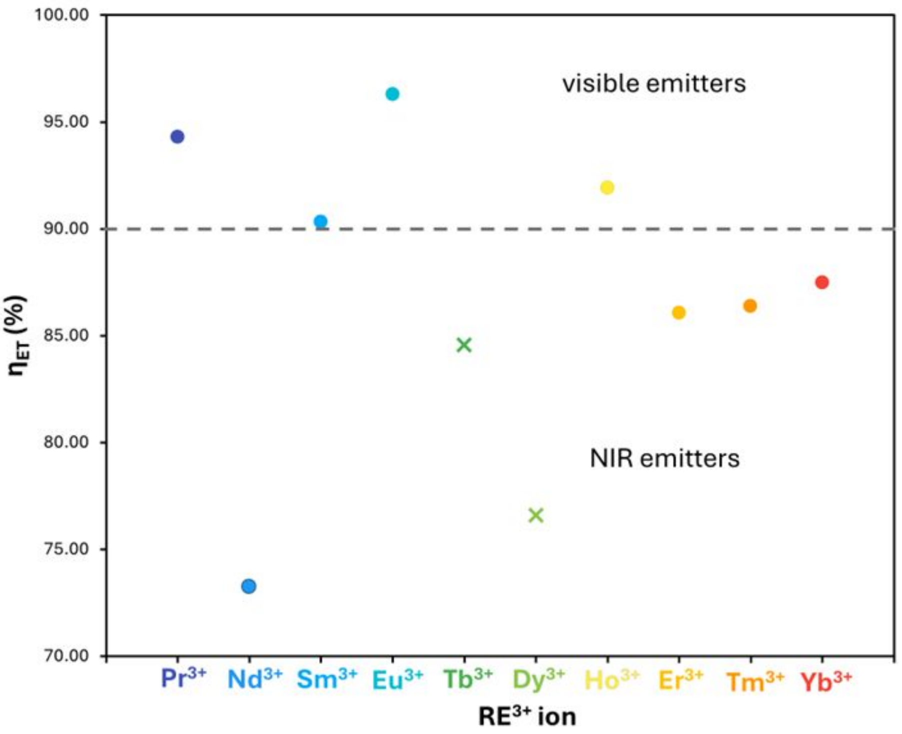

Figure S74. Graphical summary of estimated energy transfer efficiencies in **RE–*E*** complexes. Complexes not giving rise to observable RE–centered emission (**Tb–*E***, **Dy–*E***) are represented with a cross instead of the dot. The horizontal dashed line marks the limit of 90% efficiency above which **RE–*E*** visible emitters fall.

Table S28. Estimated ligand–to–metal energy transfer efficiencies in luminescent **RE–*E*** complexes.

Complex	η_{ET} (%)
**Pr–*E***	94
**Nd–*E***	73
**Sm–*E***	90
**Eu–*E***	96
**Tb–*E***	85
**Dy–*E***	77
**Ho–*E***	92
**Er–*E***	86
**Tm–*E***	86
**Yb–*E***	87

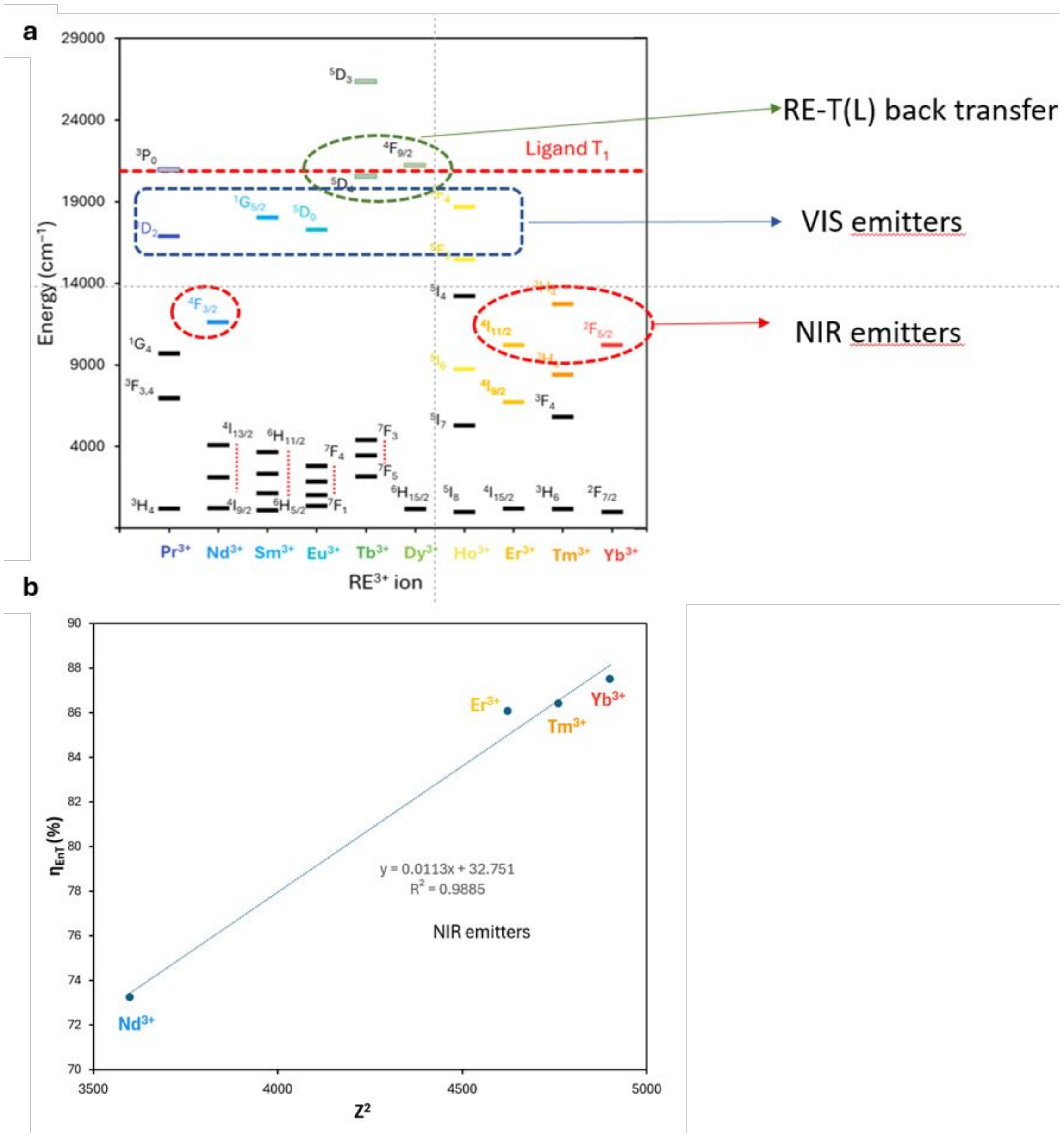

Figure S75. Linear regression of energy transfer efficiency (ηEnT) on dependence of Z^2 for NIR RE ions emitters.

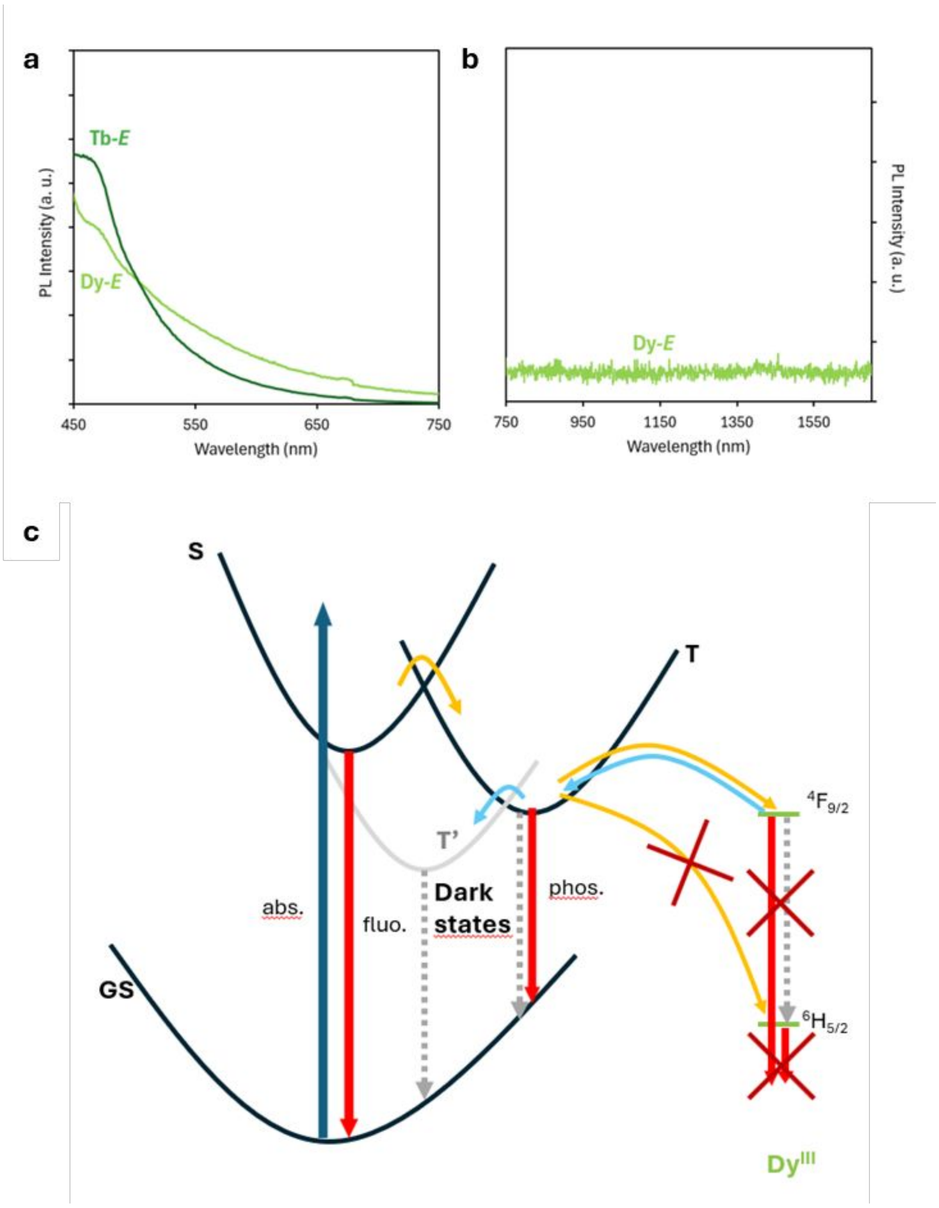

Figure S76. PL spectra of Tb-E and Dy-E acquired under Ar atmosphere a) in the visible and b) in the NIR range. Excitation wavelength was 360 nm. c) Scheme of the possible photocycle taking place in the Dy^{III} complex. Thick solid arrows represent radiative processes (abs. = absorption, fluo. = fluorescence, phos. = phosphorescence), dashed grey arrows represent non-radiative processes. Curved yellow arrows indicate the ligand-to-metal EnT while curved blue arrows refer to back EnT pathways. The efficiency of this latter mechanism prevents the low-lying $^6H_{5/2}$ level of Dy(III), potentially giving rise to NIR emission, to be populated by either cascade decay from the upper $^4F_{9/2}$ level or directly from the ligand T state. Ligand's states: GS = ground state, S = singlet state; T and T' = triplet states.

DFT Computations

In addition to configurational isomerism, hydrazones exhibit also conformational isomerism upon rotation around the C–C single bond between the iminic and aryl groups. As a matter of fact, in the solid state the *E–anti* isomer was observed. Therefore, considering the combination of the two isomerism equilibria (*E–Z* and *syn–anti*), a total of four possible isomers were possible (**Scheme S3**). Dissolution of the solid product in MeOH–d_4 confirmed the presence of only one isomer (**anti–(*E*)–L^{py}**).

The crystals structure of the ligand was used as input geometry for the generation of all four possible isomers (*E–anti, E–syn, Z–syn* and *Z–anti*) by rotation around the C=N and C–C bonds. We calculated the relative stability of the isomers trough DFT computations in MeOH using the following equations:

$$\Delta E^\circ = E^\circ\,(i - isomer) - E^\circ\,(E - anti - isomer) \quad \textbf{(S2)}$$

$$\Delta G^\circ = G^\circ\,(i - isomer) - G^\circ\,(E - anti - isomer) \quad \textbf{(S3)}$$

where E° and G° were the calculated Electronic Energies and Electronic Energies + Thermal Free Energy Correction, respectively.

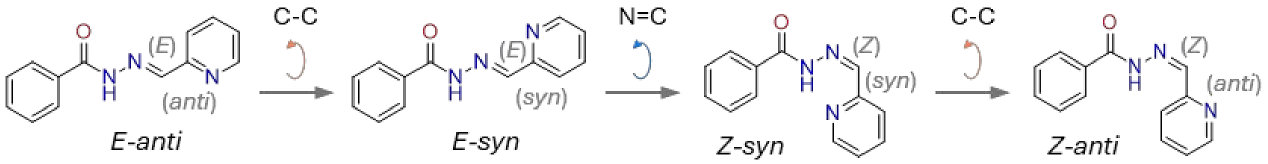

Scheme S3. Four possible isomers of **L^{py}** obtained by rotation around the N=C (*E–Z* isomerism, in light blue) and C–C (*syn–anti* isomerism, in orange) bonds.

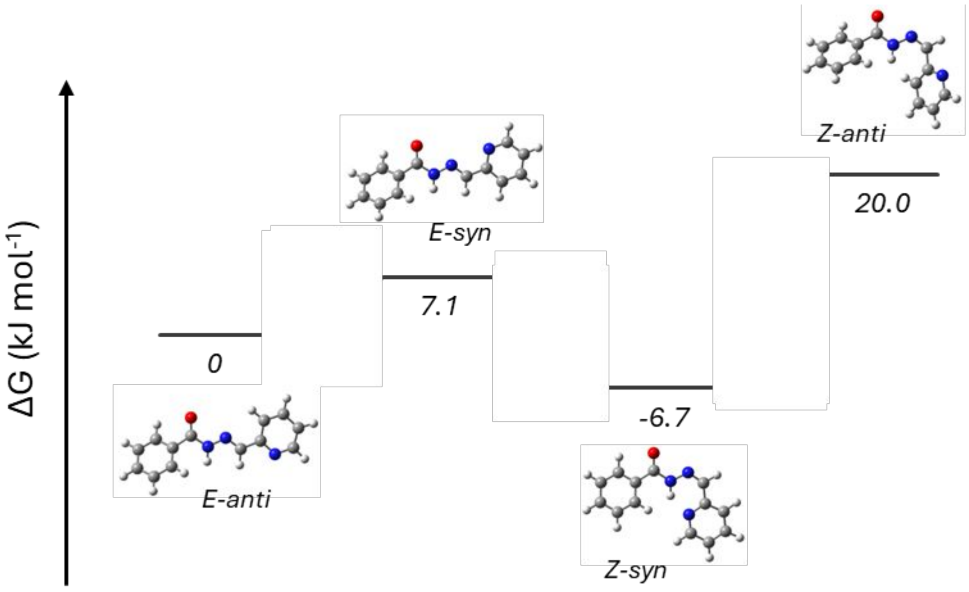

Figure S77. Energy diagram reporting the ΔG° values (in kJ mol^{-1}) for the isomers of **L^{py}** compared to that of ***(E)–anti*–L^{py}**.

Table S29. Energy and Free Energy differences for the isomers of L^{Ar} (compared to that of the *E–anti* isomers) and selected geometrical descriptors.

Ligand	Isomer	ΔE (kJ mol^{-1})	ΔG (kJ mol^{-1})	[a]φ (N=C–C–X) (°)	[b]d (D–A) (Å)	[b]ϑ (D–H–A) (°)
L^{py}	***E–anti***	0.0	0.0	179.8	–	–
	E–syn	7.0	7.1	0.8	–	–
	Z–syn	-10.6	-6.7	0.3	2.67	136.1
	Z–anti	20.4	20.0	143.0	–	–

[a]Angle reported as absolute value. Reference value for planar *syn*– and *anti*–isomers were 0 and 180°, respectively. [b]geometrical parameters (distance and angle) for intramolecular hydrogen bonds.

Time Dependent (TD–DFT) calculations were performed of the ligands, for both the most stable *E*– and *Z*–isomers, using the same level of theory. Analysis of the orbitals involved in the transitions showed that the S_1 transition was always a π–π* transition. The simulation of the absorption spectra of the ligands agreed with the experimental results as both isomeric forms can be quantitatively obtained.

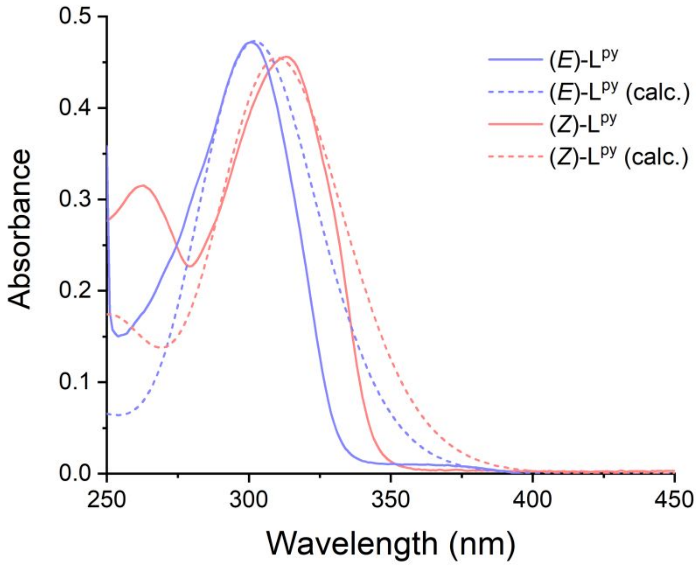

Figure S78. Experimental absorption spectra of a 30 μM MeOH solution of **(*E*)–L^{py}** before (solid light blue line) and after (solid pink line) illumination using a 395 nm wavelength. Calculated absorption spectra of **(*E*)–L^{py}** (dashed light blue line) and **(*Z*)–L^{py}** (dashed pink line) using PBE0/df2–TZVP, CPCP (methanol) computing 25 singlet states.

Table S30. Computed singlet and triplet transitions for ***(E)–anti*–L**^{py}

***(E)–anti*–L**^{py} (HOMO: 59; LUMO: 60): Total Energy, E(TD–HF/TD–DFT) = –740.753528156

Excited State 1:	Singlet–A	4.1290 eV	300.28 nm	f=1.0674	
59 –> 60		0.70074			
Excited State 2:	Singlet–A	4.4464 eV	278.84 nm	f=0.0020	
55 –> 60		–0.11806	56 –> 61		0.15573
56 –> 60		0.61962	58 –> 60		–0.24103
Excited State 3:	Singlet–A	4.5374 eV	273.25 nm	f=0.0050	
55 –> 60		0.44795	57 –> 60		–0.10539
55 –> 61		–0.11036	58 –> 60		0.41098
56 –> 60		0.24825	58 –> 61		–0.10717
56 –> 61		0.11622			
Excited State 4:	Singlet–A	4.8999 eV	253.04 nm	f=0.0170	
55 –> 60		–0.16876	58 –> 60		0.27437
57 –> 60		0.54473	58 –> 61		–0.10984
57 –> 61		–0.13700	58 –> 63		–0.14049
57 –> 63		0.12123			
Excited State 5:	Singlet–A	4.9634 eV	249.80 nm	f=0.0034	
53 –> 60		0.15403	59 –> 62		0.60234
54 –> 60		0.27179			
Excited State 6:	Singlet–A	5.0761 eV	244.25 nm	f=0.0433	
52 –> 60		0.30350	58 –> 60		–0.35575
55 –> 60		0.39280	59 –> 61		0.12293
57 –> 60		0.28897			

***(E)–anti*–L**^{py} (HOMO: 59; LUMO: 60): Total Energy, E(TD–HF/TD–DFT) = –740.810373587

Excited State 1:	Triplet–A	2.5821 eV	480.17 nm	20826 cm^{-1}	f=0.0000
59 –> 60		0.65052	59 <– 60		0.10150
59 –> 61		0.17781			
Excited State 2:	Triplet–A	3.4584 eV	358.50 nm	f=0.0000	
55 –> 61		0.11360	58 –> 60		0.30745
57 –> 60		–0.21191	58 –> 61		–0.29886
57 –> 61		0.26991	58 –> 63		0.25358
57 –> 63		0.26967	59 –> 61		0.15245
Excited State 3:	Triplet–A	3.9468 eV	314.14 nm	f=0.0000	
53 –> 60		0.26092	59 –> 60		0.12769
53 –> 62		0.10395	59 –> 61	–0.21376	
54 –> 60		–0.24539	59 –> 62	0.28581	
54 –> 62		0.33379	59 –> 64	–0.21718	
58 –> 60		0.11700			
Excited State 4:	Triplet–A	4.0013 eV	309.86 nm	f=0.0000	
56 –> 60		0.57824	56 –> 62		0.28328
56 –> 61		0.20009			

Table S31. Computed singlet transitions for ***(Z)*–*syn*–L^py^**

***(Z)*–*syn*–L^py^** (HOMO: 59; LUMO: 60): Total Energy, E(TD–HF/TD–DFT) = –740.762446492

Excited State 1:	Singlet–A	3.9956 eV	310.30 nm	f=0.6455	
59 –> 60	0.69887				
Excited State 2:	Singlet–A	4.0927 eV	302.94 nm	f=0.0004	
56 –> 60	0.38840		58 –> 60		–0.27701
57 –> 60	0.49874				
Excited State 3:	Singlet–A	4.3841 eV	282.80 nm	f=0.0048	
55 –> 60	0.52585		58 –> 60		–0.38595
55 –> 61	–0.13800		58 –> 61		0.10607
56 –> 60	–0.12291				
Excited State 4:	Singlet–A	4.7591 eV	260.52 nm	f=0.0250	
55 –> 60	0.23711		57 –> 60		0.43626
56 –> 60	–0.26271		58 –> 60		0.36053
Excited State 5:	Singlet–A	4.9078 eV	252.63 nm	f=0.1449	
53 –> 60	0.13153		58 –> 60		–0.24899
55 –> 60	–0.23210		59 –> 61		0.35576
56 –> 60	–0.32952		59 –> 62		–0.31658
Excited State 6:	Singlet–A	4.9380 eV	251.08 nm	f=0.0341	
53 –> 60	0.10591		58 –> 60		0.23940
55 –> 60	0.21858		59 –> 61		0.42994
56 –> 60	0.34872		59 –> 62		–0.21291
57 –> 60	–0.11519				
Excited State 7:	Singlet–A	5.1228 eV	242.02 nm	f=0.0460	
54 –> 60	0.17153		59 –> 62		0.53456
59 –> 61	0.40240				
Excited State 8:	Singlet–A	5.1648 eV	240.06 nm	f=0.0030	
52 –> 60	0.20347		57 –> 61		–0.28445
52 –> 62	–0.10390		57 –> 62		0.35255
55 –> 62	–0.10051		58 –> 61		0.19944
56 –> 61	–0.25205		58 –> 62		–0.14706
56 –> 62	0.29851				

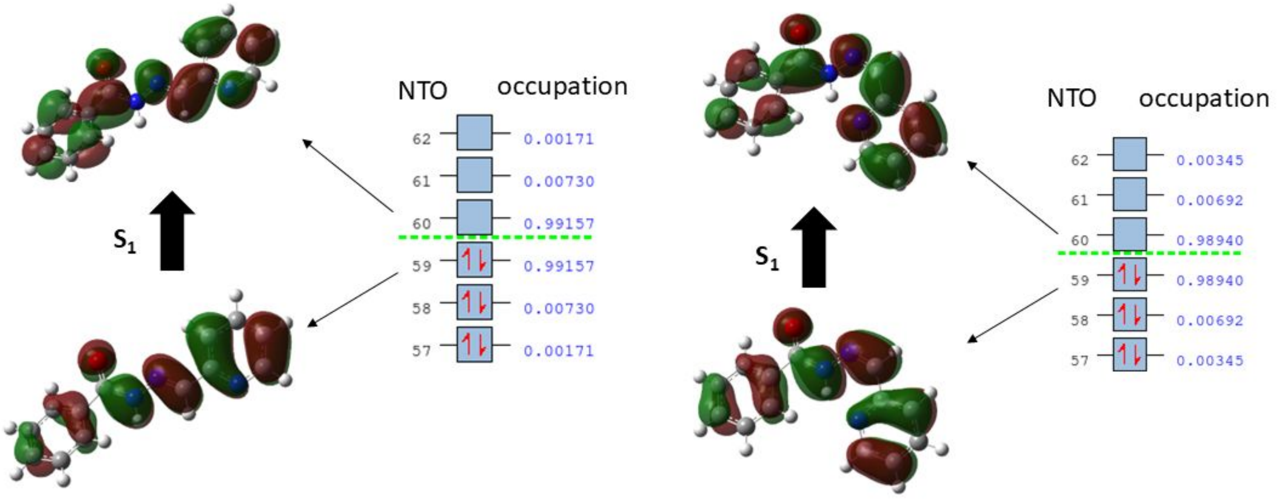

Figure S79. Natural Transition Orbitals (NTO) of ***(E)–anti*–L**^{py} (left) and ***(Z)–syn*–L**^{py} (right) using PBE0/def2–TZVP, CPCM (methanol) computing 25 singlet states. NTOs 59 and 60 exhibit the largest occupations for the first singlet excited state.

.

Table S32. Cartesian coordinates of the ligands isomers.

***(E)–anti*–L**^{py}				**(*E*)–syn–L**^{py}			
C	-2.9059	0.211465	0.047707	C	2.910872	0.215177	-0.0466
C	-3.9347	1.031595	-0.41018	C	3.946302	1.022361	0.419161
H	-3.69932	2.042836	-0.71683	H	3.71943	2.03304	0.734018
C	-5.23254	0.553981	-0.47481	C	5.23989	0.532933	0.481088
H	-6.02536	1.193833	-0.84243	H	6.03786	1.162737	0.854896
C	-5.51669	-0.74344	-0.0665	C	5.51328	-0.76341	0.062087
H	-6.53286	-1.11607	-0.11195	H	6.526158	-1.14515	0.105245
C	-4.49924	-1.55987	0.407301	C	4.48927	-1.56694	-0.41951
H	-4.71985	-2.56582	0.742293	H	4.701493	-2.57187	-0.76293
C	-3.19663	-1.0873	0.462154	C	3.190838	-1.08256	-0.47155
H	-2.42072	-1.72899	0.862934	H	2.409634	-1.71386	-0.87848
C	-1.53517	0.794726	0.101468	C	1.545003	0.810918	-0.09768
O	-1.33679	1.987388	0.236217	O	1.358156	2.006086	-0.22649
N	-0.52429	-0.11838	-0.02841	N	0.526669	-0.09426	0.027239
H	-0.74817	-1.07933	-0.25564	H	0.74564	-1.05757	0.250173
N	0.760928	0.258576	0.048954	N	-0.75648	0.291937	-0.05237
C	1.644	-0.63896	-0.14831	C	-1.63973	-0.60619	0.141817
H	1.391463	-1.67761	-0.37736	H	-1.36547	-1.64035	0.369005
C	3.070824	-0.33084	-0.07816	C	-3.07235	-0.3179	0.074389
N	3.882297	-1.3683	-0.30906	N	-3.47592	0.924472	-0.21528
C	5.192708	-1.15515	-0.25998	C	-4.77932	1.152596	-0.26843
H	5.826216	-2.01559	-0.45168	H	-5.07868	2.16923	-0.50572
C	5.758122	0.08051	0.017108	C	-5.74749	0.179528	-0.04161

Table S32 continued.

H	6.833928	0.194854	0.044234	H	-6.79825	0.431675	-0.10042
C	4.910401	1.153634	0.255845	C	-5.32697	-1.10451	0.257247
H	5.310882	2.135636	0.476003	H	-6.04315	-1.89567	0.441565
C	3.545062	0.949629	0.208503	C	-3.96689	-1.35955	0.316336
H	2.844136	1.753901	0.38741	H	-3.5959	-2.35069	0.546989
**(*Z*)–*anti*–L^{py}**				**(*Z*)–syn–L^{py}**			
C	-2.35968	0.249203	0.08721	C	-2.32289	0.271189	0.047539
C	-3.59536	0.344036	-0.54889	C	-3.63166	0.459624	-0.39094
H	-3.84479	1.258691	-1.07174	H	-3.93764	1.449984	-0.70338
C	-4.48168	-0.71896	-0.5151	C	-4.51848	-0.60314	-0.42925
H	-5.4358	-0.64236	-1.02197	H	-5.5314	-0.44862	-0.78054
C	-4.14741	-1.88115	0.169401	C	-4.10877	-1.86478	-0.01638
H	-4.84259	-2.71138	0.200332	H	-4.80266	-2.69637	-0.04165
C	-2.92543	-1.97538	0.820541	C	-2.81006	-2.05784	0.434378
H	-2.66864	-2.87283	1.369573	H	-2.49019	-3.03692	0.769272
C	-2.0313	-0.91616	0.77803	C	-1.91817	-0.99671	0.46451
H	-1.09515	-0.99705	1.318076	H	-0.91587	-1.16693	0.839767
C	-1.45733	1.434124	0.017479	C	-1.42549	1.464178	0.0655
O	-1.8768	2.56896	-0.10545	O	-1.85769	2.602898	0.06819
N	-0.12399	1.130464	0.067918	N	-0.09342	1.163347	0.063394
H	0.155781	0.159947	0.101877	H	0.257133	0.20461	-0.01751
N	0.799435	2.106537	0.145025	N	0.824766	2.139801	0.119766
C	2.039918	1.804627	0.113439	C	2.065566	1.81231	0.089323
H	2.715738	2.640933	0.26488	H	2.751961	2.651466	0.141358
C	2.692895	0.491334	-0.04742	C	2.684208	0.489374	-0.00208
N	3.805643	0.342492	0.681175	N	1.903557	-0.6016	-0.06257
C	4.462804	-0.80561	0.590269	C	2.481037	-1.79331	-0.14309
H	5.356278	-0.89695	1.199915	H	1.81293	-2.64667	-0.18953
C	4.070501	-1.85204	-0.23526	C	3.856265	-1.97097	-0.16848
H	4.649874	-2.76521	-0.2716	H	4.274865	-2.96634	-0.23487
C	2.940895	-1.68214	-1.01605	C	4.667519	-0.84808	-0.1072
H	2.612018	-2.45931	-1.69455	H	5.745979	-0.94391	-0.12477
C	2.236523	-0.49226	-0.92332	C	4.076763	0.399262	-0.02291
H	1.372012	-0.32048	-1.55201	H	4.676942	1.298969	0.026722

References

(1) Gans, P.; Sabatini, A.; Vacca, A. Investigation of Equilibria in Solution. Determination of Equilibrium Constants with the HYPERQUAD Suite of Programs. *Talanta* **1996**, 43 (10), 1739–1753.

(2) Van Dijken, D. J.; Kovaříček, P.; Ihrig, S. P.; Hecht, S. Acylhydrazones as Widely Tunable Photoswitches. *J. Am. Chem. Soc.* **2015**, *137* (47), 14982–14991.

(3) Kuhn, H. J; Braslaysky, S. E.; Schmidt, R. Chemical Actinometry (IUPAC Technical Report). *Pure Appl. Chem.* **2004**, *76* (12), 2105-2146.

(4) Sheldrick, G. M. SADABS-2008/1 - Bruker AXS Area Detector Scaling and Absorption Correction. - *Madison, Wisconsin, USA*, **2008**.

(5) Sheldrick, G. M. SHELXT - Integrated Space-Group and Crystal-Structure Determination. *Acta Crystallogr. A* **2015**, 71 (1), 3–8.

(6) Dolomanov, O. V.; Bourhis, L. J.; Gildea, R. J.; Howard, J. A. K.; Puschmann, H. OLEX2: A Complete Structure Solution, Refinement and Analysis Program. *J. Appl. Crystallogr.* **2009**, 42 (2), 339–341.

(7) Poater, A.; Cosenza, B.; Correa, A.; Giudice, S.; Ragone, F.; Scarano, V.; Cavallo, L. SambVca: A Web Application for the Calculation of the Buried Volume of N-Heterocyclic Carbene Ligands. *Eur. J. Inorg. Chem.* **2009**, No. 13 SPEC. ISS., 1759–1766.

(8) Alvarez, S. Distortion Pathways of Transition Metal Coordination Polyhedra Induced by Chelating Topology. *Chem. Rev.* **2015**, 115 (24), 13447–13483.

(9) Macrae, C. F.; Edgington, P. R.; McCabe, P.; Pidcock, E.; Shields, G. P.; Taylor, R.; Towler, M.; Van De Streek, J. Mercury: Visualization and Analysis of Crystal Structures. *J Appl. Crystallogr.* **2006**, 39 (3), 453–457.

(10) Frisch, M.J., Trucks, G.W., Schlegel, H.B., Scuseria, G.E., Robb, M.A., Cheeseman, J.R.; Scalmani, G.; Barone, V.; Petersson, G.A.; Nakatsuji, H.; Li, X.; Caricato, M.; Marenich, A.V.; Bloino, J., Janesko, B.G., Gomperts, R., Mennucci, B., Hratchian, H.P., Ortiz, J.V., Izmaylov, A.F., Sonnenberg, J.L., Williams-Young, D., Ding, F., Lipparini, F., Egidi, F., Goings, J., Peng, B., Petrone, A., Henderson, T., Ranasinghe, D., Zakrzewski, V.G., Gao, J., Rega, N., Zheng, G., Liang, W., Hada, M., Ehara, M., Toyota, K., Fukuda, R., Hasegawa, J., Ishida, M., Nakajima, T., Honda, Y., Kitao, O., Nakai, H., Vreven, T., Throssell, K., Montgomery Jr., J.A., Peralta, J.E., Ogliaro, F., Bearpark, M.J., Heyd, J.J., Brothers, E.N., Kudin, K.N., Staroverov, V.N., Keith, T.A., Kobayashi, R., Normand, J., Raghavachari, K., Rendell, A.P., Burant, J.C., Iyengar, S.S., Tomasi, J., Cossi, M., Millam, J.M., Klene, M., Adamo, C., Cammi, R., Ochterski, J.W., Martin, R.L., Morokuma, K., Farkas, O., Foresman, J.B., Fox, D.J., *Gaussian 16* (**2016**), Revision B.01, Gaussian, Inc., Wallingford CT.

(11) Weigend, F.; Ahlrichs, R. Balanced Basis Sets of Split Valence, Triple Zeta Valence and Quadruple Zeta Valence Quality for H to Rn: Design and Assessment of Accuracy. *Phys. Chem. Chem Phys.* **2005**, 7 (18), 3297–3305.

(12) Adamo, C.; Barone, V. Toward Reliable Density Functional Methods without Adjustable Parameters: The PBE0 Model. *J. Chem. Phys.* **1999**, 110 (13), 6158–6170.

(13) Barone, V.; Cossi, M. Conductor Solvent Model. *J. Phys. Chem. A* **1998**, 102 (97), 1995–2001.

(14) Paschalidis, D. G.; Tossidis, I. A.; Gdaniec, M. Synthesis, Characterization and Spectra of Lanthanide(III) Hydrazone Complexes: The X-Ray Molecular Structures of the Erbium(III) Complex and the Ligand. *Polyhedron* **2000**, 19 (26–27), 2629–2637.

(15) Christidis, P. C.; Tossidis, I. A.; Paschalidis, D. G. Bis[N -Benzoyl- N '-(2-Pyridylmethylene)Hydrazine]Trinitratocerium(III) Acetone Dihydrate. *Acta Crystallogr.* C **1999**, 55 (5), 707–710.

(16) Handbook of Near-Infrared Analysis; Burns, D. A., Ciurczak, E. W., Eds.; *CRC Press.*, **2007**.
